# Understanding the vicious cycle of myopic foresight and constrained technology deployment in transforming the European energy system

**DOI:** 10.1016/j.isci.2024.111369

**Published:** 2024-11-14

**Authors:** Jacob Mannhardt, Paolo Gabrielli, Giovanni Sansavini

**Affiliations:** 1 Institute of Energy and Process Engineering, ETH Zurich, 8092 Zurich, Switzerland

**Keywords:** Engineering, Energy sustainability, Energy systems

## Abstract

Short-term planning of myopic decision-makers jeopardizes the long-term energy transition, especially since constraints in deploying clean energy technologies further inhibit their rapid scale-up. Here, we show that the European energy transition followed myopic decision-making in the past and investigate how policy-based tools can secure the energy transition against myopic planning. Short-term decision-making in the European energy transition may fail to comply with climate goals and lead to substantial over-capacities. Carbon prices can only effectively internalize long-term climate goals if they account for constrained technology deployment, increasing to around 400 EUR/tCO_2_ in 2050. Idealized carbon prices, conversely, fail to incentivize the decarbonization of those sectors that stand at the beginning of their transition, such as renewable heating or carbon sequestration. Our exploration of myopic decision-making contributes to the understanding of the inhibiting barriers and bridges the gap between short-term decision-making and the long-term energy transition.

## Highlights

• Optimizing the European energy system under myopic foresight and system inertia
• Hindcasting highlights that in the past European emissions followed myopic behavior
• Myopic foresight and system inertia together may lead to carbon budget overshoot
• Carbon prices must consider system inertia to internalize long-term climate goals

## Graphical abstract

**Figure:**
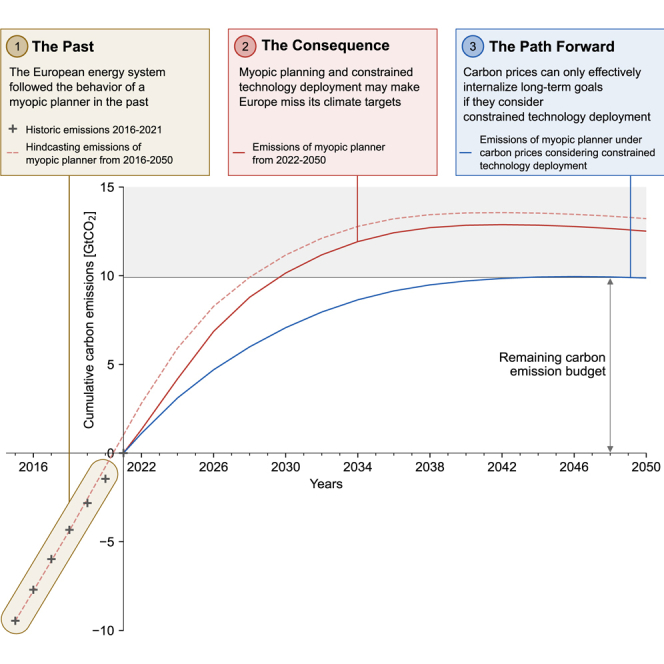

## Introduction

Current projections show that international climate actions and nationally determined contributions are insufficient to achieve the goal of limiting global warming to 1.5°C above pre-industrial levels.^1^^,^^2^^,^^3^ Europe has claimed its role as a global leader in the low-carbon energy transition,^4^ but has shown shortcomings in the necessary speed to transform the European energy system.^3^^,^^5^

Long-term climate goals stand in opposition to the short-term, myopic behavior of decision-makers.^6^^,^^7^ While the dynamics of climate change span multiple decades, political planning periods have been shown to often coincide with electoral cycles of four to five years.^8^^,^^9^ This focus on the short term perpetuates the delay of targeted climate policy.^10^^,^^11^ In the political discourse of climate change mitigation, it is often more viable to agree on ambitious long-term climate targets than to commit to specific short-term actions.^10^^,^^12^^,^^13^

The impact of myopic planning in the energy system is exacerbated by system inertia, i.e., constrained technology deployment, with past investments shaping future technology deployment.^14^^,^^15^ The deployment process entails constraints on the production of devices, global supply chains, and the local installation.^16^^,^^17^ Additionally, myopic planning may cause high carbon emissions and, therefore, contributes to a fast depletion of the remaining carbon budget that is available to restrict global warming to a desired limit.^18^ The interplay of myopic foresight and system inertia can start a vicious cycle in which the early depletion of the remaining carbon budget due to delayed investment in low-carbon technologies, in turn, demands a fast deployment of these technologies, which is inhibited by inadequate previous investments.

An abundant body of energy system optimization models (ESOMs) suggests that the transition in Europe is technically feasible (STAR Methods). To derive this conclusion, however, most ESOMs explicitly take the desired long-term outcome into account and determine the optimal sequence of investment steps that must be taken to achieve the energy transition.^19^ Basing policy recommendations on the implicit assumption that farsighted transition trajectories are inherently followed may prevent policymakers from applying effective policy instruments to enable the energy transition.^20^

Many studies acknowledge the unprecedented technology expansion rates that their results imply,^21^^,^^35^^,^^40^ but the explicit consideration of endogenous technology expansion constraints is often neglected (STAR Methods). Some works model constrained technology deployment either exogenously, i.e., assuming a constant limit on capacity additions,^25^^,^^26^^,^^27^^,^^31^ or endogenously, i.e., limiting capacity additions as a share of the existing capacity stock.^14^^,^^28^^,^^29^^,^^30^ However, only a few works emphasize the combined effect of myopic foresight and constrained technology expansion.^28^^,^^30^

The observation of heterogeneous actors has prompted the deployment of modeling tools such as agent-based models^15^ or integrated assessment models.^41^ Even though this class of models encompasses a wide array of different approaches, these models often lack transparency in how decisions are made.^42^^,^^43^ Furthermore, the low level of spatial, temporal, and technological detail misses crucial information on technological interaction in space and time.^18^^,^^44^ Centrally planned, linear optimization models may approximate the decision-making but allow the investigation of the inherent logic of transition pathways in detail. The properties of mathematical optimality, particularly in linear optimization models, allow for the direct analysis of cause and effect.^45^

This study attempts to complement the understanding of imperfect, myopically planned energy transitions by investigating the coupled impacts of myopic foresight and system inertia on the transition of the European energy system. The combination of myopic foresight and system inertia allows us to investigate the path dependencies of the European energy system in detail.

We contribute to the ongoing discussion around myopic decision-making in three main ways. First, we present evidence through hindcasting that the carbon emission trajectory of the European energy system in recent years has aligned with the behavior of a myopic planner. Our numerical results affirm the observations from political science^6^^,^^7^ and serve as the premise for our following analyses.

Second, we show how myopic foresight may fail to achieve our climate goals by delaying the investment in renewable electricity and heating technologies. We highlight that a myopic decision-maker managing a cumulative carbon budget may initially overspend and then attempt to correct its actions by rapidly increasing renewable capacity, resulting in high costs and renewable overcapacities.

Third, we extend current literature by investigating how myopic decision-makers can be nudged to internalize long-term climate goals through policy instruments. Here, we apply farsighted carbon prices as one market-based tool to a myopic decision-maker. We show that planning for carbon prices is only sufficient if they consider system inertia. Insufficient carbon prices delay the transition of those renewable sectors that stand at the beginning of their transition.

## Results

### Hindcasting post-Paris Agreement reveals myopic behavior of European energy system

We cross-combine the two dimensions of decision-making that inhibit the energy transition to quantify the coupled dynamics of myopic foresight and system inertia: (1) the level of foresight, from perfect to myopic foresight and (2) the level of technology deployment, from instantaneous to constrained deployment.

The European energy transition is modeled as a linear optimization problem minimizing net-present cost from 2022 until 2050 to describe the investment and operation in the electricity and heating sector under each decision-making scenario. Experimental Procedures and Supplemental Methods S2 and S3 report on the model and methodology in detail.

The remaining carbon emission budget for the European electricity and heating sector to limit the global temperature increase to 1.5°C with a probability of 50% is 9.9 GtCO_2_ when assuming an equal per-capita distribution (Experimental Procedures). The optimizer aims to adhere to the time-independent carbon budget by formulating it as a soft constraint, i.e., overshooting is permitted but heavily penalized. The remaining carbon budget is known to the optimizer every year; however, in the early years, a myopic planner may not anticipate reaching the budget.

We follow the observation that the foresight of political myopia relates to electoral cycles^9^ and assume a foresight horizon length of 4 years in the reference case (Experimental Procedures). The deployment constraint on capacity additions is expressed as a function of the capacity expansion knowledge.^14^ The knowledge stock describes the technical and industrial expertise to install a certain technology, which depreciates over time as industrial knowledge fades.

We consider the transition of the European energy system to be feasible if the system (1) stays within the carbon emission budget and (2) reaches carbon neutrality in 2050.^46^ Crucially, a feasible model outcome does not imply a feasible transition in the real world, i.e., model feasibility does not necessitate realism.

**Figure 1:**
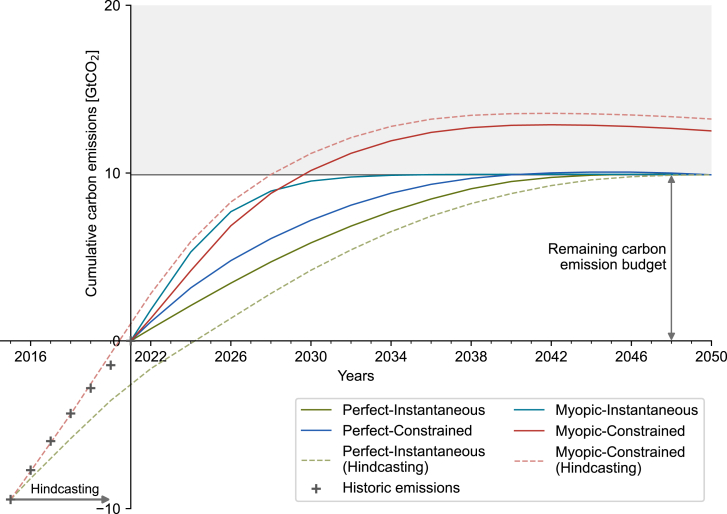
Cumulative carbon emissions from 2022 until 2050 for each scenario from Perfect-Instantaneous to Myopic-Constrained The historical carbon emissions of the electricity and heating sectors for 2016–2021 are shown as gray crosses,^47^^,^^48^ and the carbon emissions obtained from hindcasting Perfect-Instantaneous (green) and Myopic-Constrained (red) from 2016 until 2050 are shown as dashed lines. The optimizer is not informed of historical emissions or capacity additions after the beginning of the optimization. The gray area indicates an overshoot of the remaining carbon emission budget.

Since the adoption of the Paris Agreement in December 2015 until the end of 2021, the European electricity and heating sector caused 9.5 GtCO_2_ of carbon emissions (gray crosses in Figure 1), almost equivalent to the remaining carbon emission budget from 2022 until 2050.^47^^,^^48^ In other words, the system caused roughly the same quantity of emissions in 6 years as what it would equitably be allocated over the following 29 years.

We show by hindcasting that this behavior does not match the decision-making of a farsighted planner: In the time span from 2016 until 2021, an optimal planner with perfect foresight and instantaneous technology deployment (Perfect-Instantaneous, dashed green line in Figure 1) would have only emitted 6.8 GtCO_2_ by directly anticipating the carbon emission budget and hence transitioning to renewable technologies early on. In contrast, a myopic decision-maker facing technology deployment constraints (Myopic-Constrained, dashed red line in Figure 1) behaves similarly to the observed historic trend, causing 10.5 GtCO_2_ of carbon emissions.

### Acting myopically with system inertia may make us miss our climate goals

Figure 1 shows the cumulative emissions from 2022 until 2050 for each of the four modeling scenarios. Both pathways with perfect foresight (Perfect-Instantaneous and Perfect-Constrained) show a smooth evolution to reach the carbon emission budget in 2050. Because of the constrained technology deployment, Perfect-Constrained cannot install the same renewable capacity as Perfect-Instantaneous in the first years (Figure S1). Thus, the carbon emissions of Perfect-Constrained in early years exceed those of Perfect-Instantaneous and, therefore, require stronger reductions in annual carbon emissions in later years to remain within the budget. However, the foresight of 29 years is sufficient to take the necessary steps to comply with the carbon emission budget until 2050.

Myopic-Constrained, with myopic foresight and constrained technology deployment, is the only pathway that does not result in a feasible energy transition. In the beginning, the myopic pathways (Myopic-Instantaneous and Myopic-Constrained) do not anticipate the carbon emission budget and, therefore, cause high annual carbon emissions, which are similar to the historically observed emissions (around 1.3 GtCO_2_ per year).

Once the myopic pathways anticipate the impending overshoot of the emission budget, Myopic-Constrained cannot radically install new capacities because of system inertia. Thus, Myopic-Constrained overshoots the carbon emission budget around the year 2030 (gray area in Figure 1).

Overshooting the carbon emission budget is heavily penalized, i.e., we assume that Europe will strongly adapt its behavior and take all necessary and possible measures to limit the overshoot after failing to achieve the 1.5°C target. Assuming a response of decision-makers to carbon overshoot, it then takes Myopic-Constrained until the 2040s to stabilize the carbon emissions, which results in a cumulative carbon emission overshoot of 26% (2.6 GtCO_2_).

### Delaying initial investments results in renewable over-capacities and high costs later

**Figure 2:**
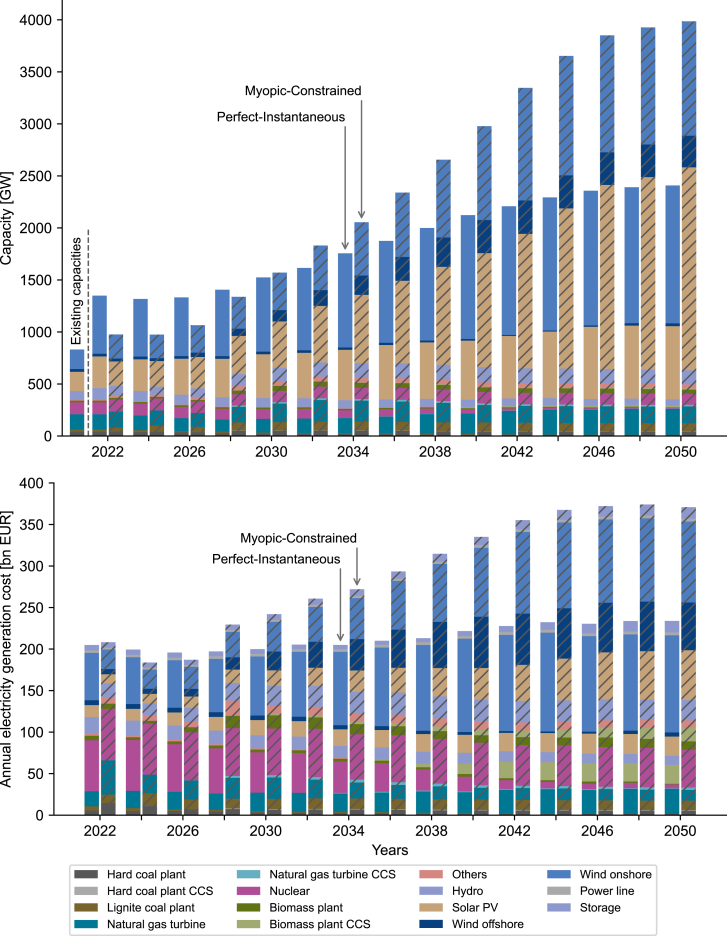
Electricity generation capacities (top plot) from 2022 until 2050 for Perfect-Instantaneous (left bars) and Myopic-Constrained (right bar, hatched) scenarios Existing capacities in 2022 added as reference. Annual electricity generation costs (bottom plot) for Perfect-Instantaneous and Myopic-Constrained, including all upstream and downstream costs (Supplemental Procedure S4). Oil and waste power plants are aggregated to “Others”. Heat generation capacities are shown in Figure S2.

The top plot of Figure 2 shows the electricity generation capacity of the Perfect-Instantaneous and Myopic-Constrained scenarios from 2022 until 2050. For the sake of clarity, we omit to show the capacities of the other two scenarios here (see Figure S1). All pathways show a common transition to renewable electricity technologies, with onshore (light blue bars in Figure 2) and offshore wind turbines (dark blue), and solar photovoltaics (PV, yellow) contributing the largest share in 2050, with capacity shares around 85% for all scenarios.

Perfect-Instantaneous shows a smooth transition with early adoption of renewable technologies to achieve the energy transition, despite the high investment costs of renewable technologies. In particular, Perfect-Instantaneous adds more than 500 GW of electricity generation capacity in 2022, the first year of the optimization, most of which are onshore wind and solar photovoltaics (see existing capacities in the top plot of Figure 2).

Without anticipation of the carbon emission budget, Myopic-Constrained delays the investment in renewable technologies and instead continues to operate existing fossil fuel technologies. In the last year before anticipating the carbon emission budget, wind turbines and solar PV only contribute to the electricity generation capacities by 51% in Myopic-Constrained (in 2024), in comparison to more than 60% in Perfect-Instantaneous and Perfect-Constrained.

When the carbon budget is then reached, and the emissions must be drastically reduced, the low initial investments in renewable technologies are insufficient to speed up the transition to a low-carbon energy system. Myopic-Constrained must then continue to operate existing fossil technologies in the transition phase between 2030 and 2040 to supply the electricity and heat demand and thus overshoots the carbon budget.

Myopic-Constrained must invest in significant over-capacities of renewable technologies to drastically reduce the emissions after overshooting the budget, which results in a total renewable capacity of around 3,350 TW in 2050 (more than 60% higher than Perfect-Instantaneous). The renewable over-capacities lead to low utilization of renewables and stranded conventional assets (Figure S3). Furthermore, myopic decision-making necessitates investment in second-best technologies, such as offshore wind (Figure S1).

The decarbonization of the heating sector is highly dependent on electrifying heat production (Figure S2). With limited biomass availability, the decarbonized heating sector will be mainly supplied by heat pumps and direct electric heating. Early carbon emission reductions in the electricity sector have a double impact by subsequently reducing emissions in the heating sector as well. In contrast, if the electricity sector is not decarbonized early on, the heating sector will also remain carbon-intensive.

Not only does myopic planning lead to an overshoot of the carbon budget, but the investment in over-capacities and stranded assets furthermore raises the cost for electricity generation costs in the long run (bottom plot of Figure 2). Delaying investments lowers the annual electricity generation costs of Myopic-Constrained by 16 bn. EUR (−8%) in 2024 in comparison to Perfect-Instantaneous. However, in 2050, the Myopic-Constrained pathway shows 137 bn. EUR higher generation costs (+59%). The cumulative net present cost in 2050 of Myopic-Constrained is 23% increased in comparison to Perfect-Instantaneous (7,495 bn. EUR and 6,106 bn. EUR, respectively). Perfect-Instantaneous can achieve lower costs in 2022 than Myopic-Constrained by directly installing renewable technologies to counteract the high costs of the energy crisis in 2022^49^ (see high natural gas cost for Myopic-Constrained in 2022 in Figure 2).

### Projected carbon prices must consider constrained technology deployment to be effective

Achieving a successful energy transition of the European energy system requires overcoming the barriers of myopic foresight and constrained technology deployment. Therefore, we investigate how a myopic planner can be nudged to consider and adapt to long-term climate goals.

Ideally, a myopic decision-maker could overcome the barriers by (1) planning for a longer foresight horizon and (2) incentivizing the industry to expedite technology deployment. We find that both levers can, in fact, enable a successful transition (Figure S4). A foresight horizon of 10 years is sufficient for a wide range of technology expansion rates to stay within the remaining carbon budget. On the other hand, faster deployment of technologies enables a more flexible change in strategy and thus allows for shorter foresight horizons. If all technologies could be expanded as fast as solar PV has been in the past (see STAR Methods), a foresight horizon of four years is sufficient (Figure S4).

However, given the unlikeliness that myopic decision-makers will suddenly start expanding renewable technologies with longer foresight and low system inertia, policy tools must be designed to internalize long-term climate goals into short-term decision-making. Research shows that effective climate policy consists of a mix of different policies, including subsidies, tariffs, efficiency mandates, and carbon pricing.^50^ The most common market-based tool to account for the future cost of climate change is to apply a carbon pricing mechanism, i.e., a carbon tax or a carbon cap scheme.^51^ Here, we only focus on carbon pricing since optimization models allow us to extract optimal carbon prices from the dual variable of the carbon emission constraint.

The rationale is that the bounded foresight of real-world decision-makers can be overcome by enforcing an optimal carbon price so that the energy transition and the climate goals can be achieved. However, carbon pricing has previously been criticized for not capturing the barriers to the energy transition.^52^ To plan policies and investments appropriately, myopic planners in governments and firms alike must project carbon prices that can effectively achieve a successful transition.^53^

To investigate if perfectly farsighted carbon prices can enable a successful transition under myopic foresight, we apply them to Myopic-Constrained, namely:

(1) Perfect-Instantaneous and Perfect-Constrained are optimized (Figure 1); Perfect-Constrained is optimized for three different technology expansion rates (10%, 13%, and 29%, which correspond to the maximum historic expansion rates of offshore wind, onshore wind, and solar PV; see Experimental Procedures).
(2) The marginal carbon abatement costs are obtained as the dual variable, i.e., the shadow price, of the carbon emission constraint in Perfect-Instantaneous and Perfect-Constrained.
(3) The carbon abatement cost curves are applied as an exogenous carbon price to Myopic-Constrained under the three technology expansion rates without any further carbon emission constraint.

**Figure 3:**
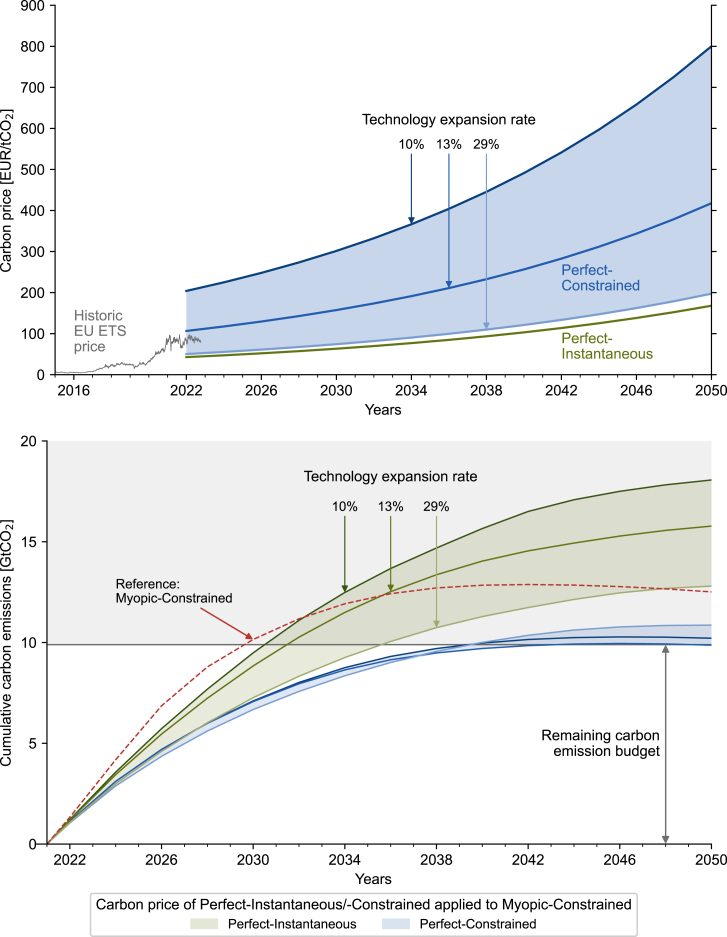
Impact of applying the marginal carbon abatement cost (upper panel) of Perfect-Instantaneous (green) and Perfect-Constrained (blue) as an exogenous carbon price on the cumulative carbon emissions of Myopic-Constrained (lower panel) The carbon prices of Perfect-Instantaneous and Perfect-Constrained are extracted as the dual variable of the total annual carbon emission constraint (Equation S11) and applied to Myopic-Constrained without any additional carbon emission constraints. Importantly, the model is not calibrated with historic carbon prices^54^ and thus has no knowledge thereof. The technology expansion rates are varied (10%, 13%, 29%). The gray area indicates an overshoot of the carbon emission budget. Reference curve for Myopic-Constrained from Figure 1 is reported for the sake of comparison (dashed red line in lower panel). The discount rate is set to 5% (Supplemental Procedure S1.4).

In Perfect-Instantaneous, the carbon price increases from around 43 EUR/tCO_2_ in 2022 to 168 EUR/tCO_2_ in 2050 (upper panel in Figure 3). Conversely, the constrained technology deployment in Perfect-Constrained necessitates (1) the use of more expensive technologies and (2) stronger emission reductions in later years. Hence, the carbon price in Perfect-Constrained for the reference technology expansion rate (13%, the maximum historic expansion rate of onshore wind, Experimental Procedures) is more than double that of Perfect-Instantaneous, with an initial carbon price of 107 EUR/tCO_2_ in 2022, which increases to 418 EUR/tCO_2_ in 2050. A more constrained technology expansion leads to a further increased carbon price, more than quadruple that of Perfect-Instantaneous, for an expansion rate of 10%. As a point of reference, the historically observed carbon price in the EU ETS (from 2016 until October 2023^54^) is currently in the range of the carbon price obtained under Perfect-Constrained (Figure 3). Note that the EU ETS and the energy system modeled in this study do not fully overlap in the considered sectors. The carbon price of Myopic-Constrained is not shown in Figure 3, because it reaches the artificial overshoot penalty cost once the budget overshoot is anticipated, which has limited practical relevance beyond the methodological function of treating the budget as a soft constraint (STAR Methods).

Applying the carbon price of the idealized pathway, Perfect-Instantaneous, to the imperfect pathway of Myopic-Constrained leads to a budget overshoot of between 29% and 83%, depending on the technology expansion rate (Figure 3). Furthermore, the carbon price does not ensure a carbon-neutral energy system in 2050.^52^

On the other hand, accounting for constrained technology deployment in carbon prices can be an effective measure to ensure compliance with the climate goals. In 2050, the myopic transition pathways under the carbon prices of Perfect-Constrained show cumulative carbon emissions close to the carbon budget (overshoot below 10%).

**Figure 4:**
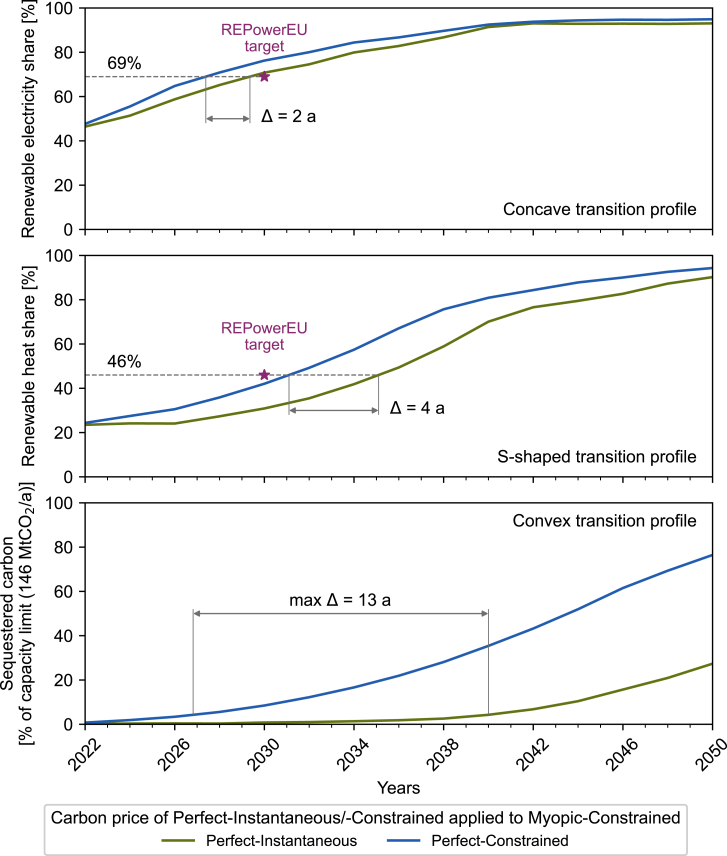
Impact of applying the marginal carbon abatement cost of Perfect-Instantaneous (green) and Perfect-Constrained (blue) as an exogenous carbon price on the renewable electricity share (top), renewable heating share (center), and sequestered carbon (bottom, as a percentage of capacity limit) of Myopic-Constrained from 2022 to 2050 for reference technology expansion rates (13%) The targets of the REPowerEU plan^55^ for renewable electricity (69%) and heating production (46%) in 2030 are shown as purple stars. The delay Δ describes the time interval between reaching the same share under the two carbon prices.

Figure 3 highlights that carbon price projections that do not account for system inertia are insufficient to incentivize the necessary transformation. This becomes even more evident when analyzing the evolution of the renewable electricity share (top row in Figure 4), renewable heating share (middle row), and sequestered carbon (bottom row) of Myopic-Constrained under the exogenous carbon price of Perfect-Instantaneous and Perfect-Constrained. Under the carbon price of Perfect-Instantaneous, the transition of each sector is delayed in comparison to the scenario with the carbon price of Perfect-Constrained. However, the sectors show significant differences in how the insufficient carbon price impacts the transition.

The electricity sector, which is already strongly renewable today, transitions swiftly to a final renewable electricity share of more than 90%. The REPowerEU target^55^ of 69% renewable electricity production by 2030 is achievable, with the myopic scenario under the carbon price of Perfect-Instantaneous being at most 2 years delayed to achieve the goal in comparison to the Perfect-Constrained carbon price.

The European heating demand is still mostly supplied by fossil fuels, but renewable heating technologies such as heat pumps have received increasing attention and investments over the past years. With the widespread deployment of renewable heating technologies waiting in the wings, the heating sector requires high policy support now for a fast transition.^56^ Here, the drawbacks of the underestimated carbon price become apparent. The REPowerEU target^55^ of 46% renewable heat generation by 2030 is almost achieved under the carbon price of Perfect-Constrained. The myopic scenario under the Perfect-Instantaneous carbon price delays the heating transition considerably and reaches the REPowerEU target 4 years later than under the Perfect-Constrained carbon price.

If the decarbonization of both the electricity and the heating sectors is delayed, the only remaining option to reduce carbon emissions is the uptake of carbon removal technologies. However, Figure 4 shows that carbon sequestration is delayed the most by insufficient carbon price projections (up to 13 years). In particular, carbon prices must be high enough to stimulate investments even when more cost-effective measures exist in the short term. Figure 4 highlights that the delay occurs at the beginning of the deployment, where the model under the Perfect-Instantaneous carbon price shows no significant uptake until the mid-2030s.

In summary, projecting carbon prices that neglect the impact of system inertia fails to incentivize the transformation of those sectors that stand at the beginning of their transition (convex or S-shaped transition curve in Figure 4). Consequently, only sectors that are already strongly renewable (concave transition curve in Figure 4) are incentivized to transition even under low carbon price projections.

## Discussion

Myopic planning and system inertia put the European energy transition at risk. We show by hindcasting that the European energy system did not follow perfectly farsighted emissions trajectories over the past years but caused significantly higher carbon emissions than a decision-maker with perfect foresight would have done to comply with the carbon budget. A myopic planner under system inertia planning with a cumulative carbon budget, however, closely replicates the observation of real-world emissions. Perfectly farsighted pathways may provide valuable insights into cost-optimal pathways to reach carbon neutrality, however, it is imperative not to confuse farsighted pathways with explorative assessments of the feasibility of the energy transition.

Our analysis shows that staying within the remaining carbon emission budget in Europe may become infeasible for a real-world planner under myopic foresight and constrained technology deployment. First, the transition to renewable technologies is delayed because myopic planners do not anticipate long-term climate goals in the form of the carbon emission budget.^26^^,^^31^ Instead, the optimizer continues to operate existing fossil fuel plants, such as coal power plants, which leads to high carbon emissions. Second, once the budget is anticipated, the system inertia inhibits the radical deployment of renewable technologies. As a result, the carbon emission budget is overshot by around 26% when considering reference values for technology expansion rates and assuming that the decarbonization strategy is radically adapted once the budget is reached.

We confirm existing literature^31^ that myopic foresight leads to stranded conventional assets. Furthermore, we highlight that myopic foresight can lead to significant renewable over-capacities. In times of lower electricity demand, large quantities of wind and solar power must then be curtailed, which hampers their economic competitiveness and is, therefore, avoided by private investors.^57^ Since primarily private citizens carry the financial burden of heating technologies, the prospect of stranded heating assets jeopardizes the affordability and justice of the energy transition.^58^ The ramifications of delaying the heating transition and installing new fossil heating systems should be clearly communicated in the currently ongoing debates around the phase-out of fossil fuel heating in Europe.^58^^,^^59^

After observing a delayed transition under myopic foresight, we raise the question of how to enable a feasible transition considering myopic foresight and system inertia. Longer foresight horizons and higher technology expansion rates can both help to reduce the carbon budget overshoot.^30^ Climate goals can be achieved by planning the investment and operation of the European energy system with a foresight of at least 10 years, independent of the technology expansion rate. If the political planning foresight continues to coincide with electoral cycles shorter than 5 years,^9^^,^^60^ the foresight will most likely be insufficient to comply with the climate goals. While desirable, increasing the foresight horizons of myopic decision-makers is highly challenging, since the reasons for myopic planning are multifaceted and go beyond purely technical considerations.^61^

Effective climate policy internalizes the future cost of climate change to nudge myopic planners to transition. A wide array of climate policies exist, with a varying degree of efficacy.^50^ In this work, we investigate carbon pricing as one possible climate policy tool; mainly because our model allows us to extract optimal carbon prices from the dual variable of the carbon emission constraint. A feasible energy transition could only be enabled by carbon prices that account for the complexities and imperfections of decision-making and effectively internalize the long-term climate goals. We find that a carbon price from an idealized pathway (Perfect-Instantaneous) results in a budget overshoot between 30% and 85%. The necessary carbon price to comply with the climate goals must be higher to account for system inertia: from around 100 EUR/tCO_2_ in 2022 to 400 EUR/tCO_2_ in 2050 for the reference case (more than double that of Perfect-Instantaneous). The reform of the EU ETS in 2023 has tightened the ETS emissions cap,^62^ which may signal higher policy credibility and farsighted planning.^63^ On the other hand, some researchers attribute the currently dropping carbon prices to market myopia.^64^

This study shows that projecting carbon prices that account for system inertia is essential to incentivize the decarbonization of the energy system, especially for those sectors that are at the beginning of their transition, such as the heating and the carbon removal sectors. Myopic decision-making under insufficient carbon prices may delay the heating transition so much that the REPowerEU target of 46% renewable heat generation by 2030^55^ would be missed by up to 5 years. Since carbon sequestration does not yet exist at a large scale, policy tools such as carbon pricing must incentivize deployment today, if a relevant contribution to the energy transition is desired.

Understanding the impact of carbon pricing on the decarbonization pathway and its limitations is important for policymakers to develop complementary policies such as subsidies or renewable generation targets to existing carbon pricing schemes, i.e., mainly the EU ETS and the national carbon taxing schemes.^65^ This study shows that myopic decision-making delays the necessary investments in technologies before they reach economic competitiveness but that these barriers can be overcome by stronger and better-informed price and policy signals. A better understanding of the economic incentives can accelerate the energy transition across all sectors to achieve the climate goals.

### Limitations of the study

We conduct a thorough sensitivity analysis of relevant parameters in Supplemental Method S1 to assess the robustness of the observed trends for a wide range of input data assumptions. Our claims are robust against the uncertainty of the tested input data; in particular, we find that myopic decision-making under constrained technology deployment misses the climate goals across all sets of input parameters.

Our investigation of the impact of myopic foresight and constrained technology deployment on the European energy transition opens up several research avenues that simultaneously highlight some limitations of this work.

First, we assume an equal foresight horizon for the investment planning and the operation of the energy system. This implies that a decision-maker will plan the operation of its assets, including fossil fuel plants, with the same foresight as it plans the investment in new capacities. In reality, however, investment planning shows significantly longer foresight^66^ than operation planning.^4^^,^^49^ In this study, we neglect lead times in construction since technologies with a longer lead time than the myopic foresight horizon would never be built. This underestimates the existing challenges of installing large power plants such as nuclear power plants.^67^ The actual foresight length for real myopic planners remains an open question in the literature.

Second, while the presented linear optimization problem allows for a thorough analysis of the expansion and transition dynamics with high technological and temporal detail, it neglects the diverse decision-making structure of the heterogeneous actors in the European energy system. These more complex interconnections of myopic decision-makers certainly impact the description of myopic foresight. Energy system modelers and political scientists should cooperate to derive a better description of the foresight dynamics. We exclude the transport sector from our centralized cost minimization because investment decisions in the transport sector are highly decentralized and do not only follow cost-optimal decisions.^68^ However, we acknowledge that the transport sector causes high emissions, and electrified transport may add a substantial electricity demand.

Third, the presented study is deliberately agnostic of any particular policy in order to explore the range of decarbonization dynamics rather than a projection of how the energy system will evolve under a certain policy. The investigation of carbon prices in Figure 3 is chosen as an exemplary market-based policy item to internalize long-term goals, but we do not intend to claim that carbon pricing is the most effective, let alone the only climate policy. Certainly, the most effective climate policy is a mix of different tools.^50^ We refrain from exogenously imposing further existing European policies such as the EU Emission Trading System (EU ETS),^69^ national carbon taxing schemes,^65^ coal and nuclear phase-outs,^70^ or a ban of new fossil-based heating systems.^56^ Nevertheless, it should be noted that the EU ETS provides an annual emission trajectory that will begin to include the residential heating sector from 2027 onwards (EU ETS II^62^). However, some researchers have raised questions about the viability of the current EU ETS II setup.^71^ In many European countries, the EU ETS is complemented by national carbon taxing schemes.^65^

Finally, this study explicitly models endogenous technology deployment constraints due to historic installations and spillover effects and can, therefore, capture the dynamics of technology uptake well. Our selection of investigated expansion rates, obtained from the historic capacity additions in Europe, indicates how future pathways might compare against the past deployment but does not attempt to predict how specific technologies will be expanded in the future.^72^^,^^73^ The presented approach does not replace an in-depth analysis of the complex real-life technology expansion dynamics. In this work, we focus on the European energy system and thereby assume that the technology deployment is mainly constrained by the local and continental technology environment, i.e., local public acceptance,^74^ regulation,^73^ and installation capacities.^75^ The impact of global supply chains is only captured implicitly in this simplified deployment constraint. Global supply chains have certainly impacted past deployment and, therefore also our obtained expansion rates, but we neglect the direct contribution of global generation capacities on future expansion. Future research could investigate the impact of international supply chains on the technology deployment constraints in detail.

## Resource availability

### Lead contact

Further information and requests for resources should be directed to and will be fulfilled by the lead contact, G.S. (sansavig@ethz.ch).

### Materials availability

No materials were used in this study.

### Data and code availability

• The data needed to reproduce all figures and results have been deposited at Zenodo: https://zenodo.org/records/11074289 and are publicly available as of the date of publication. Accession numbers are listed in the key resources table.
• All original code has been deposited at Zenodo: https://zenodo.org/records/11074289 and is publicly available as of the date of publication. Accession numbers are listed in the key resources table.
• Any additional information required to reanalyze the data reported in this paper is available from the lead contact upon request.

## Acknowledgments

The research published in this report was carried out with the support of the 10.13039/501100005380Swiss Federal Office of Energy (10.13039/501100005380SFOE) as part of the SWEET PATHFNDR project. The authors bear sole responsibility for the conclusions and the results.

## Author contributions

Conceptualization, J.M., P.G., and G.S.; Methodology, J.M., P.G., and G.S.; Software, J.M.; Validation, J.M.; Formal Analysis, J.M., P.G., and G.S.; Data Curation, J.M.; Writing – Original Draft: J.M.; Writing – Review and Editing: J.M., P.G., and G.S.; Visualization: J.M., P.G., and G.S.; Supervision: P.G. and G.S.; Project Administration: G.S.; Funding Acquisition: P.G. and G.S.

## Declaration of interests

The authors declare no competing interests.

## STAR★Methods

### Key resources table

**Table:** 

REAGENT or RESOURCE	SOURCE	IDENTIFIER
**Deposited data**		

Original data to reproduce figures and results	This paper	https://doi.org/10.5281/zenodo.11074289

**Software and algorithms**		

Original code to reproduce figures and results	This paper	https://doi.org/10.5281/zenodo.11074289

### Method details

#### System representation and complexity

We model the transition of the European electricity and heating sector of 28 European countries (EU27 + CH, NO, UK - CY, MT) in the open-source energy transition optimization framework ZEN-garden (Zero-emission Energy Networks), developed at the Reliability and Risk Engineering Laboratory (RRE) at ETH Zurich. ZEN-garden optimizes the design and operation of energy system models to investigate transition pathways toward decarbonization. The optimization problem is solved using the commercial solver Gurobi 9.5.1.^76^ All input data, the source code of the optimization framework, and raw result files are accessible in the Zenodo Repository https://zenodo.org/records/11074289. The Supplemental information is available in the separate document “Supplemental information - Understanding the vicious cycle of myopic foresight and constrained technology deployment in transforming the European energy system”.

The model is formulated as a linear cost-minimization problem, minimizing the total net-present cost for all planning periods of the foresight horizon for all countries. The goal of the optimizer is to supply the final energy demands of all carriers in each time step at the lowest cost by installing and operating conversion technologies, transport technologies, and storage technologies. The detailed mathematical formulation and the parametrization of the model are described in Supplemental Methods S2 and S3.

The transition of the European energy system is investigated from 2022 to 2050. To model the incremental capacity additions in detail while still allowing for computational feasibility, we optimize the investment and operation of the European energy system in 2-year intervals. Thus, the transition pathway outlined in this study is more highly inter-yearly time-resolved than most long-term transition pathway models, which generally use 5-year increments.^18^^,^^26^^,^^38^

We develop a novel intra-yearly time series representation approach of the hourly-resolved input data to enable this high inter-yearly time resolution. Namely, we aggregate the input time series (8760 hours per year) to 100 representative time steps through hierarchical clustering.^77^ Then, we resolve the storage level variable with more time steps to enable short-term and long-term storage. In detail, for each change in the sequence of representative time steps, we add an additional time step in the sequence of storage level time steps. The time series representation approach is detailed in^49^ and in Supplemental Method 2.6, and the error caused by the approximation is assessed in Supplemental Method 1.11.

#### Remaining carbon emission budget

We impose a cumulative carbon emission budget from 2022 until 2050 but do not constrain the annual carbon emissions (except for intermediate targets in 2030 and 2050), since such an exogenous yearly emission trajectory is an internalization of a perfectly farsighted transition strategy (Table 1). To limit the global temperature increase to 1.5°C with a 50% likelihood, the IPCC projects that global cumulative carbon emissions must remain below 500 GtCO_2_ between 2020 and 2050.^1^ In 2020 and 2021, 34.2 GtCO_2_ and 36.3 GtCO_2_ were emitted globally,^78^ respectively. Hence, the remaining carbon emission budget from 2022 until 2050 is 429.5 GtCO_2_. Note that we follow the IPCC methodology in that we only consider direct carbon dioxide emissions for the cumulative emissions.^1^

**Table 1:** Relevant studies of energy system transition models

Case	Transition pathway	Level of foresight	Carbon emission constraint	Deployment constraint	Exemplary references
1	No	–	–	–	Pickering et al.^21^; Trondle^22^; Jacobson et al.^23^
2	Yes	Perfect foresight	Trajectory	None	Pietzcker et al.^24^
				Exogenous	Howells et al.^25^; Heuberger et al.^26^; Gerbaulet et al.^27^
				Endogenous	Keppo and Strubegger^28^; Loulou et al.^29^; Fuso Nerini et al.^30^
			Price	None	Howells et al.^25^; Löffler et al.^31^; Pietzcker et al.^24^
				Exogenous	Babrowski et al.^32^
				Endogenous	Leibowicz et al.^14^
			Budget	None	Pietzcker et al.^24^
				Exogenous	Howells et al.^25^; Gerbaulet et al.^27^; Löffler et al.^31^
				Endogenous	Fais et al.^33^; Pye et al.^34^
3a	–	Myopic foresight	Trajectory	None	Victoria et al.^18^; Pedersen^35^; Victoria et al.^36^
				Exogenous	Heuberger et al.^26^; Gerbaulet et al.^27^; Abuzayed and Hartmann^37^
				Endogenous	Keppo and Strubegger^28^; Fuso Nerini et al.^30^
3b			Price	None	–
				Exogenous	Heuberger et al.^26^; Bogdanov et al.^38^; Babrowski et al.^32^; Abuzayed and Hartmann^37^
				Endogenous	Fuso Nerini et al.^30^
3c			Budget	None	–
				Exogenous	Löffler et al.^31^
				Endogenous	–

An endogenous technology deployment constraint describes that capacity additions are limited by past investments, whereas an exogenous technology deployment constraint is an external, absolute capacity addition limit. For an extensive review of studies without transition pathways, see.^39^

The optimal allocation of the remaining global budget to the 28 European countries included in this study is not agreed upon by the scientific community.^79^^,^^80^^,^^81^ We assume an equal per-capita distribution of the remaining carbon emission budget. Some scholars argue that Europe has overused the carbon budget in the past and, therefore, has a historic responsibility to emit less in the future to allow other regions their equitable share of cumulative emissions.^81^^,^^82^ However, the discussion of the fair allocation of the remaining budget goes beyond the scope of this study. We note that an investigation of the feasibility of the energy transition under different definitions of just budgets could contribute to the literature.

Accounting for the projected population development of all countries,^83^ the share of the remaining carbon emission budget for the considered countries from 2022 until 2050 is 5.9%, or 25.4 GtCO_2_. We include the emissions of the three IPCC sectors: i) energy industries (1.A.1), ii) commercial/institutional (1.A.4.a), and iii) residential (1.A.4.b) (obtained from the European Environment Agency^47^). The carbon emissions of the corresponding sectors of the UK are obtained from.^48^ In 2021, the electricity and the residential and commercial building sectors accounted for 39% of total emissions.^47^^,^^48^ The remaining budget for the electricity and building sectors is then 9.9 GtCO_2_ for all 28 countries. We note that these sectors are among the easiest sectors to decarbonize; hence, one could argue that their share of the remaining budget should be lower than the current share of emissions. However, we follow existing studies in this allocation approach.^36^

The optimizer is allowed to overshoot the carbon emission budget, but the excess emissions are penalized at a high cost of 5000 EUR/tCO_2_. It is important to note that this overshoot price is not comparable to a carbon price, e.g., in Figure 3, but it penalizes the cumulative carbon budget overshoot. A pathway that does not overshoot at the end of its foresight horizon does not have to pay the overshoot cost. The overshoot price is chosen to be more expensive than any available mitigation strategy; hence, overshooting the budget is the last option the optimizer will resort to. We show in Supplemental Procedure 1.9 that the value of this parameter does not impact the numerical results for a wide range of values (-50% to + 50%). The optimizer attempts to limit the overshoot as much as possible; however, the ambition with which society reduces emissions will depend on the political and societal will to comply with our climate goals.^13^

We set annual carbon emissions limits for 2030 and 2050, as they are defined as legally binding emissions targets in the European Climate Law.^46^ In particular, in 2050, carbon net-neutrality must be reached (0 GtCO_2_). The carbon emission limit in 2030 of the electricity and building sector for all 28 considered EU and non-EU countries is based on the projections of the EU to comply with the “Fit for 55” target.^84^ Using the historic emissions in 1990 and 2019, the carbon emission limit for all 28 countries in 2030 is calculated as 1.06 GtCO_2_ per annum in 2030.^47^^,^^48^ We do not include the annual emission cap of the EU ETS I or II.

#### Optimization under perfect and myopic foresight

The figure below schematically shows the optimization under perfect and myopic foresight. Under perfect foresight, the energy system is optimized for the entire time horizon. Hence, the optimizer has perfect knowledge of all future parameters, can anticipate long-term goals, and plan accordingly. On the contrary, under myopic foresight, the foresight horizon is reduced. The optimizer has no knowledge of any parameter beyond the foresight horizon. In this study, the carbon emission budget is imposed as a time-independent constraint on cumulative emissions. Thus, the optimizer knows the remaining budget at all times.

**Figure:**
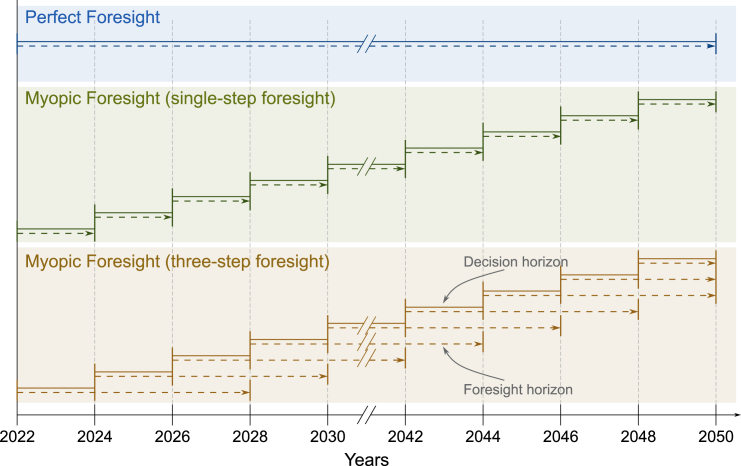
Schematic depiction of optimization under perfect (blue) and myopic foresight (green and brown) Myopic foresight with single-step (green) and exemplary three-step foresight (brown). The investment and operation are projected for the foresight horizon (dashed lines) and then irreversibly decided for the decision horizon (solid line).

After obtaining the solution for the first myopic optimization, the capacity additions and cumulative carbon emissions of the first planning period, i.e., the decision horizon, are saved and used as additional parameters in the following optimization. The foresight horizon is shifted by one planning period and optimized again.

In all presented pathways, the foresight horizon length is two planning periods, i.e., four years, unless indicated differently (Figure S2). This is shorter than the current momentum of investments in renewable technologies suggests.^85^ However, the foresight horizon also encapsulates the operation of the energy system, which is generally planned with less consideration of the future, especially during energy crises.^49^^,^^86^

#### Constrained technology deployment

The technological landscape must be ramped up to diffuse a technology, i.e., setting up global supply chains for renewable devices, domestic production, streamlining standardization and licensing, and training skilled workers to install devices.^16^^,^^17^ The constraint on technology deployment is expressed as a function of past installations, based on the formulation by Leibowicz et al.^14^ The potential capacity additions in each country are determined by:

1. The existing capacity knowledge stock, multiplied by a constant technology expansion rate.
2. Knowledge spillover effects from other countries, represented by a constant knowledge spillover rate. Thus, countries can profit from existing capacities in other countries, which is especially relevant for the common market in the EU.^87^
3. Unbounded market shares and capacity additions to represent the initial unbounded entry into a niche market, after which the first two effects gain importance.

The knowledge stock describes the technical and industrial expertise to install a technology corresponding to past capacity additions. Historic evidence suggests that the knowledge of energy technologies is depreciated by 10-40% per year as industrial knowledge fades over time.^14^ We assume a conservative knowledge depreciation rate of 10% each year, following Leibowicz et al.^14^ As an example, a capacity addition of 1 GW in the year 2000 contributes to the knowledge stock in 2020 by $0.12\;\text{GW}={\left(1-0.1\right)}^{\left(2020-2000\right)}\;\text{GW}$.

To avoid a feedback loop where country A profits from the knowledge in country B and simultaneously country B profits from the knowledge in country A, we introduce an additional constraint, which limits the capacity additions in the entire continent without external spillover effects. Thus, countries can profit from spillover effects from other countries, however, we assume that the industrial capacities in Europe are limited and can only be distributed across the continent.

The future expansion behavior of specific technologies is highly uncertain and will be influenced by a multitude of effects that did not manifest in the past.^72^^,^^73^ Hence, the extrapolation of historic dynamics to foresee the evolution of a specific technology in the future is prone to lead to false accuracy and potentially biased results. To avoid producing model artifacts, we select a constant expansion rate for all technologies, i.e., assume that all technologies can show the same expansion dynamics.

To this end, we investigate the historical deployment of all relevant electricity generation technologies in Europe and select the observed expansion rates of the most promising technologies for the European energy transition, namely solar PV and onshore and offshore wind. The highest capacity additions for all years and countries define the maximum expansion rate of each technology. We obtain technology expansion rates of 10%, 13%, and 29% for offshore wind, onshore wind, and solar PV, respectively, and a knowledge spillover rate of 7% ($R^{2}$ = 0.754). The detailed procedure is described in Supplemental Procedure S3.11.

#### Scientific literature on energy system transition pathways

There are generally three ways in which the long-term goals are considered in the literature (Table 1):

Case 1: No transition pathway is modeled, i.e., the energy system is optimized for a snapshot year in which carbon neutrality is achieved.
Case 2: The transition is optimized under perfect foresight, i.e., the optimizer has perfect knowledge of the future climate goals.
Case 3: The optimizer has myopic foresight, i.e., does not have knowledge of all future states, but the climate goals are exogenously imposed on the decision-maker by a perfectly farsighted annual emission trajectory (3a) or carbon price (3b) to ensure compliance with climate goals. Hence, the myopic optimizer is relieved of planning ahead.

## Supplemental information

Document S1. Figures S1–S45 and Tables S1–S8

Table S9. Cost assumptions for all technologies, related to STAR Methods

## Footnotes

Supplemental information can be found online at https://doi.org/10.1016/j.isci.2024.111369.
