# Supplementary material for: Understanding the vicious cycle of myopic foresight and constrained technology deployment in transforming the European energy system
Source: iScience. 2024 Nov 14;27(12):111369. doi: 10.1016/j.isci.2024.111369 (PMC11665420; doi:10.1016/j.isci.2024.111369)
Supplement: Document S1. Figures S1–S45 and Tables S1–S8 [file mmc1.pdf]

**iScience, Volume *27***

## Supplemental information

# Understanding the vicious cycle of myopic foresight and constrained technology deployment in transforming the European energy system

**Jacob Mannhardt, Paolo Gabrielli, and Giovanni Sansavini**

# Contents


S1 Sensitivity analysis . . . . . . . . . . . . . . . . . . . . . . . . . . . . S7
S1.1 Cost parameter assumption . . . . . . . . . . . . . . . . . . . S8
S1.2 Electricity and heat demand assumption . . . . . . . . . S14
S1.3 Fuel price assumptions . . . . . . . . . . . . . . . . . . . . . . . . S18
S1.4 Discount rate . . . . . . . . . . . . . . . . . . . . . . . . . . . . . . S19
S1.5 Variable technology expansion rates . . . . . . . . . . . . S23
S1.6 Knowledge spillover rate . . . . . . . . . . . . . . . . . . . . . . S26
S1.7 Hydropower capacity limit . . . . . . . . . . . . . . . . . . . . S29
S1.8 Carbon storage capacity limit . . . . . . . . . . . . . . . . . S33
S1.9 Carbon budget overshoot cost . . . . . . . . . . . . . . . . . S36
S1.10 Heat supply substitution . . . . . . . . . . . . . . . . . . . . . S38
S1.11 Temporal resolution . . . . . . . . . . . . . . . . . . . . . . . . S41
S2 Nomenclature and formulation of the optimization problem in ZEN-garden . . . . . . . . . . . . . . . . . . . . . . . . . . . . . . S43
S2.1 Nomenclature of the optimization problem . . . . . . . S43
S2.2 Objective function . . . . . . . . . . . . . . . . . . . . . . . . . . S47
S2.3 Energy balance and carbon emission constraints . . . . S49
S2.4 Operational constraints . . . . . . . . . . . . . . . . . . . . . . S51
S2.5 Investment constraints . . . . . . . . . . . . . . . . . . . . . . . S53
S2.6 Time series aggregation and representation . . . . . . . S55
S2.7 Proof of storage level monotonicity . . . . . . . . . . . . . S56
S3 Input data of case study . . . . . . . . . . . . . . . . . . . . . . . S58
S3.1 Scope of case study . . . . . . . . . . . . . . . . . . . . . . . . . S58
S3.2 Existing capacity - electricity sector . . . . . . . . . . . . S61
S3.3 Capacity limit - electricity sector . . . . . . . . . . . . . . S62
S3.4 Capacity factors - electricity sector . . . . . . . . . . . . . S63
S3.5 Existing capacity - heating sector . . . . . . . . . . . . . . S64
S3.6 Capacity limit - heating sector . . . . . . . . . . . . . . . . S66
S3.7 Supply substitution - heating sector . . . . . . . . . . . . S68
S3.8 Energy carriers - import availability, prices, carbon intensity . . . . . . . . . . . . . . . . . . . . . . . . . . . . . . . . . . S70
S3.9 Carbon supply chain . . . . . . . . . . . . . . . . . . . . . . . . . S72
S3.10 Cost parameters . . . . . . . . . . . . . . . . . . . . . . . . . . . . S73
S3.11 Technology expansion rates . . . . . . . . . . . . . . . . . . . S77
S4 Calculation of stranded assets and total annual technology cost S80
S5 Comment on the use of rolling horizon and myopic foresight to reduce computational burden . . . . . . . . . . . . . . . . . . . . . . S81

## Supplemental figures

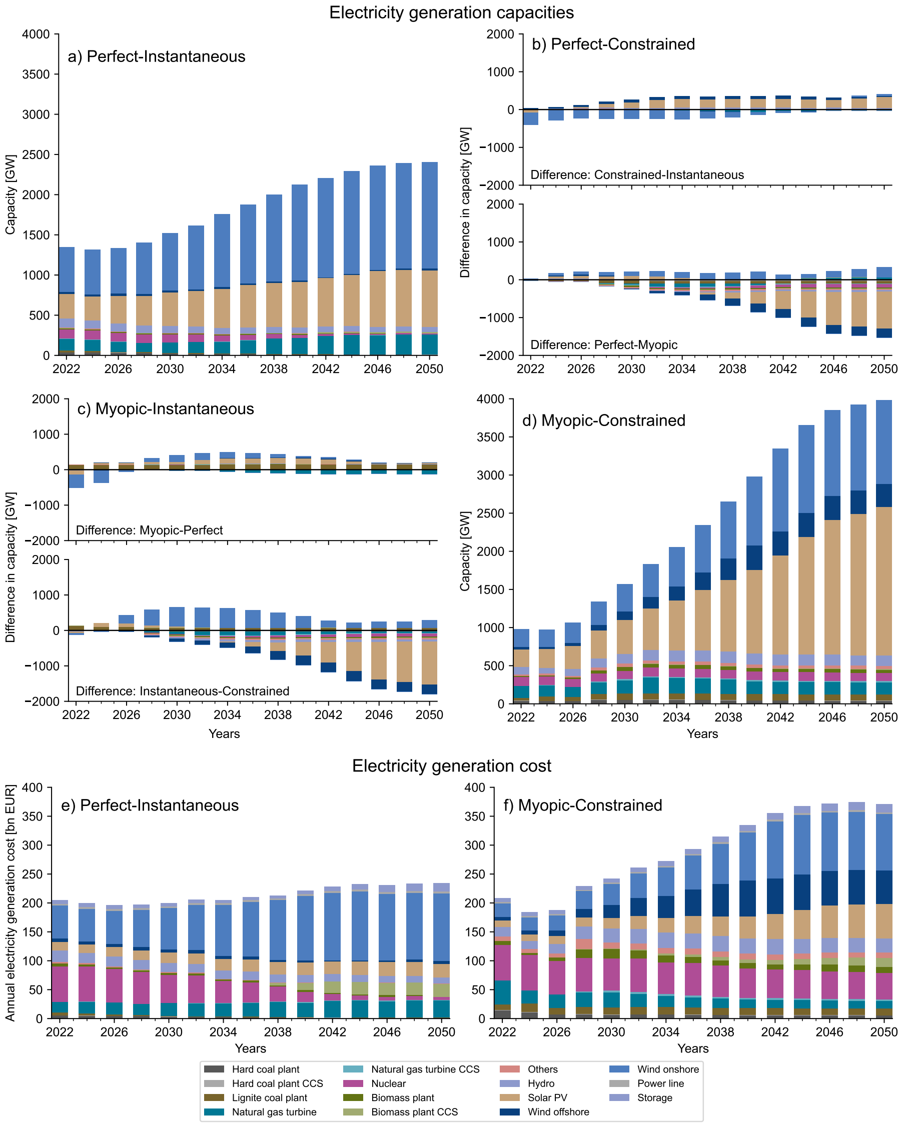


**Fig. S1** Electricity generation capacities (a-d) from 2022 until 2050 for each scenario in the complexity matrix. Perfect-Constrained (b) and Myopic-Instantaneous (c) are shown as the difference in capacity to Perfect-Instantaneous (a, top row) and Myopic-Constrained (d, bottom row). Negative values indicate higher capacities in Perfect-Instantaneous or Myopic-Constrained. Annual electricity generation costs for Perfect-Instantaneous (e) and Myopic-Constrained (f), including all upstream and downstream costs (see S4). Oil and waste power plants are aggregated to “Others”. Related to Figure 2.

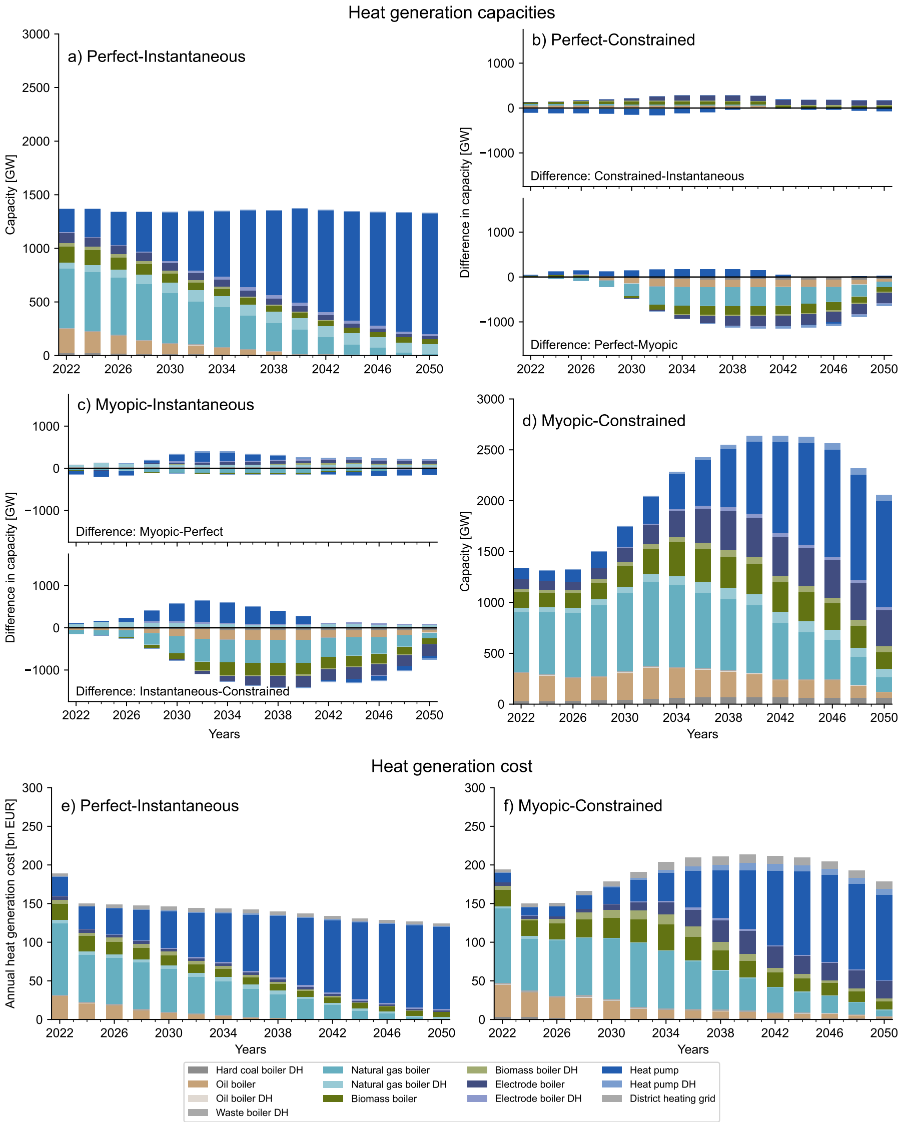


**Fig. S2** Heat generation capacities (a-d) from 2022 until 2050 for each scenario in the complexity matrix. Perfect-Constrained (b) and Myopic-Instantaneous (c) are shown as the difference in capacity to Perfect-Instantaneous (a, top row) and Myopic-Constrained (d, bottom row). Negative values indicate higher capacities in Perfect-Instantaneous or Myopic-Constrained. Annual heat generation costs for Perfect-Instantaneous (e) and Myopic-Constrained (f), including upstream and downstream costs. District heating technologies are abbreviated "DH". Related to Figure 2.

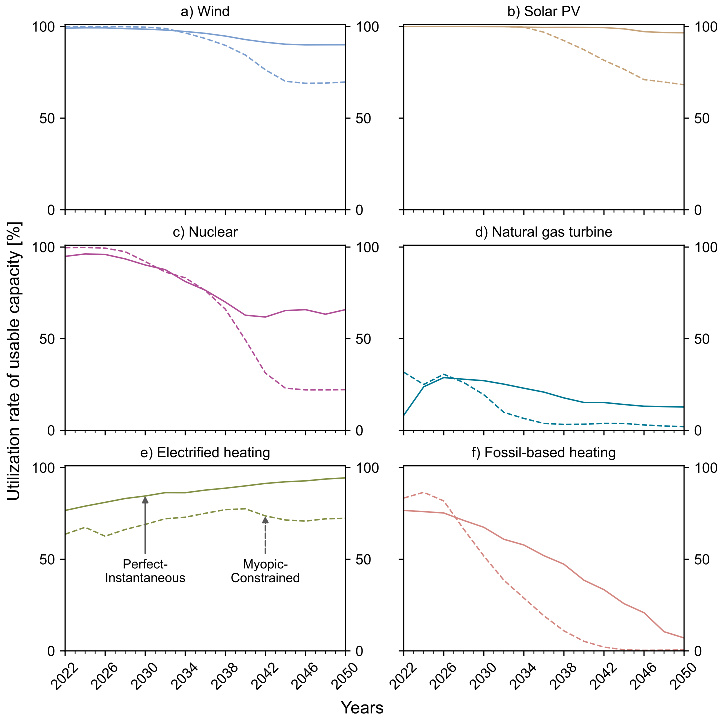


**Fig. S3** Utilization rate of wind, solar PV, nuclear, natural gas, and electrified and fossil-based heating capacities for Perfect-Instantaneous (solid line) and Myopic-Constrained (dashed line) from 2022 to 2050. 100% utilization is achieved when the technology dispatches the maximum possible energy, taking the capacity factor into account. Onshore and offshore wind are aggregated to "Wind". Natural gas turbines with and without carbon capture and storage (CCS) are aggregated to "Natural gas turbine". "Electrified heating" is composed of heat pumps and direct electric heating. "Fossil-based heating" encompasses natural gas boilers, hard coal boilers, and oil boilers. Due to the drastic reduction of emissions and higher penetration of renewables in Myopic-Constrained after anticipating the emission budget, the utilization of conventional technologies is strongly reduced, and the assets become stranded. Fossil-based heating technologies are especially vulnerable to becoming stranded since the decarbonization of the heating sector will go hand in hand with deploying electrified heating. Related to Figure 2.

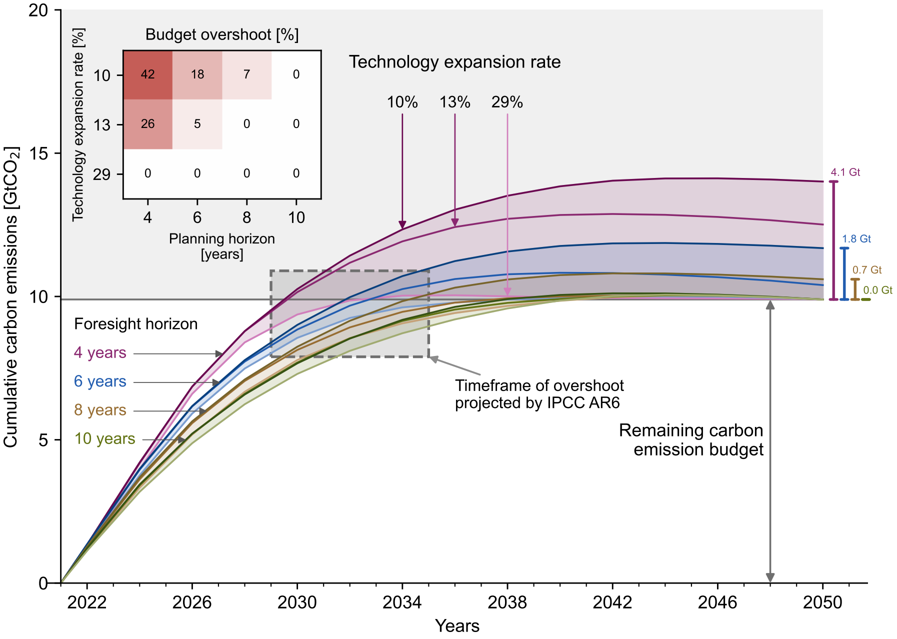


**Fig. S4** Cumulative carbon emissions from 2022 to 2050 for varying technology expansion rates and foresight horizons for Myopic-Constrained decision-making. The grey area indicates an overshoot of the carbon emission budget. The technology expansion rates are varied from 10% to 13% to 29%. The foresight horizon is set to 4, 6, 8, and 10 years. The IPCC AR6 projects that the 1.5°C target will be missed between 2030 and 2035 (50% likelihood) [1]. With an increasing planning horizon, the carbon budget starts being anticipated earlier and thus depleted at a later point, which reduces the overshoot of the budget in the long term. the spread of the overshoot between the highest and the lowest technology expansion rate decreases for longer planning horizons. Planning ahead and sticking to the deployment plans are the most important tools for reducing the impact of system inertia, i.e., an uncertain future industrial landscape or limitations on imported technologies. Vice versa, an energy system with low system inertia due to high technology expansion rates is less affected by myopic foresight [2]. Related to Figure 1.

# Method details

## S1 Sensitivity analysis

We investigate the impact of various parameters and modeling assumptions on the results by conducting a series of sensitivity analyses. They target the following classes of assumptions:

1. Cost parameter assumptions (S1.1, related to S3.10)
2. Electricity and heat demand assumption (S1.2, related to S3.8)
3. Fuel price assumptions (S1.3, related to Table S7)
4. Discount rate (S1.4, related to Eq. (S2))
5. Variable technology expansion rates (S1.5, related to S3.11)
6. Knowledge spillover rate (S1.6, related to S3.11)
7. Hydropower capacity limit (S1.7, related to S3.3)
8. Carbon storage capacity limit (S1.8, related to Table S8)
9. Carbon budget overshoot cost (S1.9, related to Eq. (S9))
10. Heat supply substitution (S1.10, related to S3.7)
11. Temporal resolution and number of operational time steps (S1.11, related to S2.6)

While all assumptions impact the numerical value of the results to varying degrees, the general observations in this study are robust against all investigated uncertainties. In particular, all variations of Myopic-Constrained overshoot the carbon emission budget and all variations of all other models achieve to remain within the budget in 2050.

## S1.1 Cost parameter assumption

The range of cost parameter assumptions from S3.10 impacts the numeric value of the cumulative carbon emissions (Fig. S5). For Perfect-Instantaneous, lower cost parameters ("min") lead to lower annual carbon emissions in early years, whereas higher cost parameters ("max") slightly increase carbon emissions in early years. However, Perfect-Instantaneous still remains within the carbon emission budget. Lower cost parameters lead to lower cumulative carbon emissions in Myopic-Constrained; analogously, higher cost parameters lead to higher emissions. However, Myopic-Constrained continues to overshoot the budget significantly. Cost assumptions show no significant impact on the carbon emissions of Perfect-Constrained. Neglecting the cost learning rates has a negligible impact on carbon emissions.

Intuitively, higher cost parameters lead to a significantly increased net present cost (around +40% in 2050 across models, Fig. S6). Conversely, lower cost parameters decrease the net present cost by around 30% in 2050. Neglecting learning effects results in a small cost increase (around +3% in 2050).

Minimum cost parameters increase the deployment of offshore wind and solar PV (Fig. S7). In the case of Myopic-Instantaneous, the optimizer installs a large number of waste power plants (aggregated with oil power plants in "others"), because of its low minimum cost assumption in Table S9. Maximum cost parameters generally increase the deployment of onshore wind and solar PV (Perfect-Instantaneous, Myopic-Instantaneous, Perfect-Constrained) and slightly decrease deployment in Myopic-Constrained (Fig. S8). Neglecting the cost reduction because of technology learning reduces the deployment of solar PV and slightly impacts onshore wind deployment (Fig. S9).

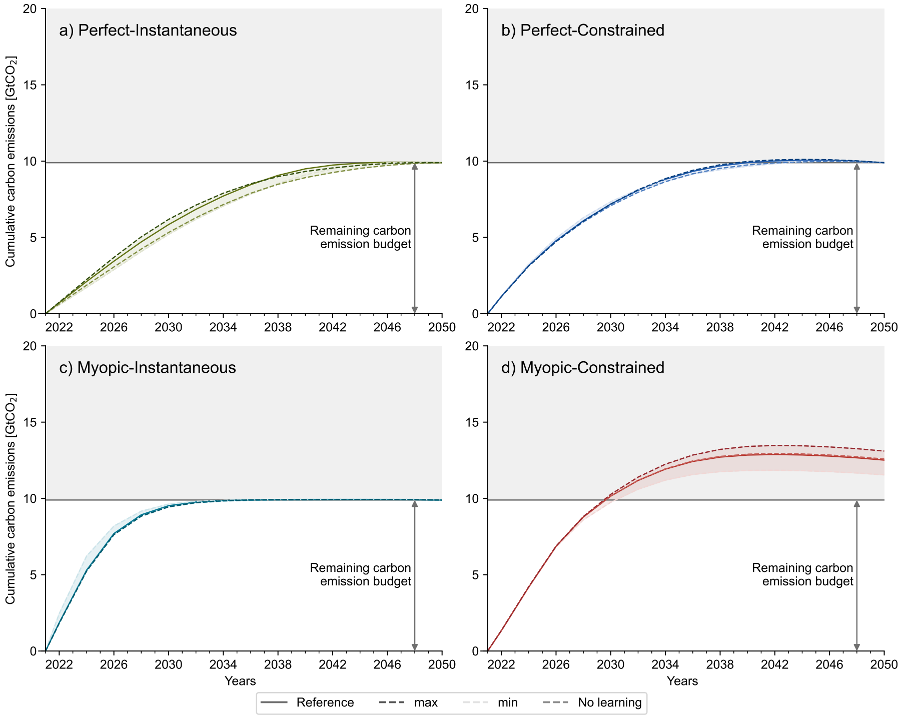


**Fig. S5** Cumulative carbon emissions for different cost parameter assumptions (S3.10): Average (solid line), minimum (dashed light line), maximum (dashed dark line), average without learning rates (dashed medium line). Related to Figure 1.

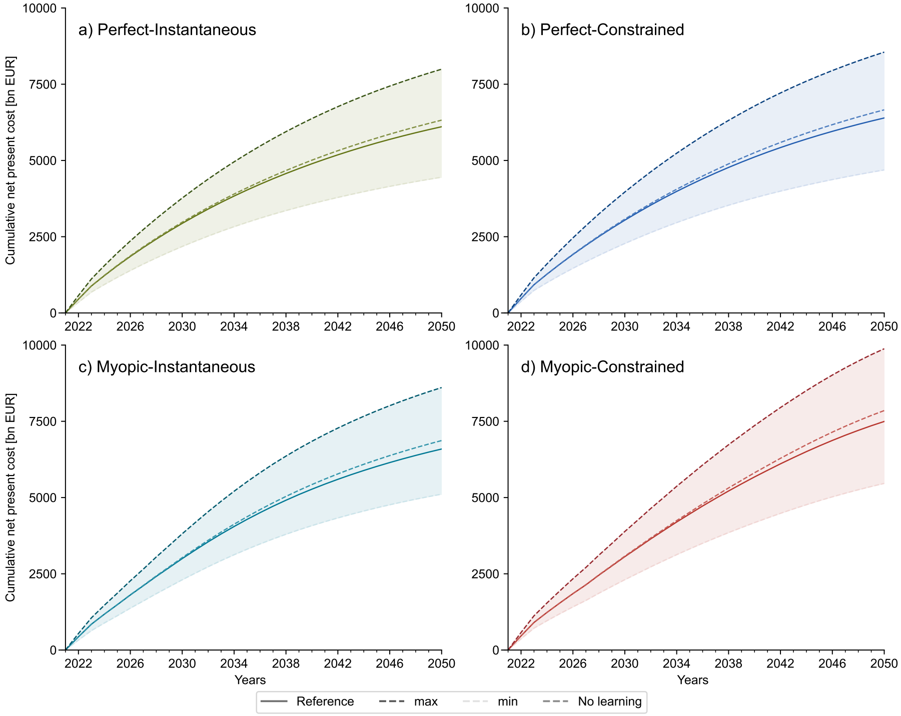


**Fig. S6** Cumulative net present cost (NPC) for different cost parameter assumptions (S3.10): Average (solid line), minimum (dashed light line), maximum (dashed dark line), average without learning rates (dashed medium line). Carbon overshoot cost and cost for shed demand are excluded from NPC calculations. Related to Figure 1.

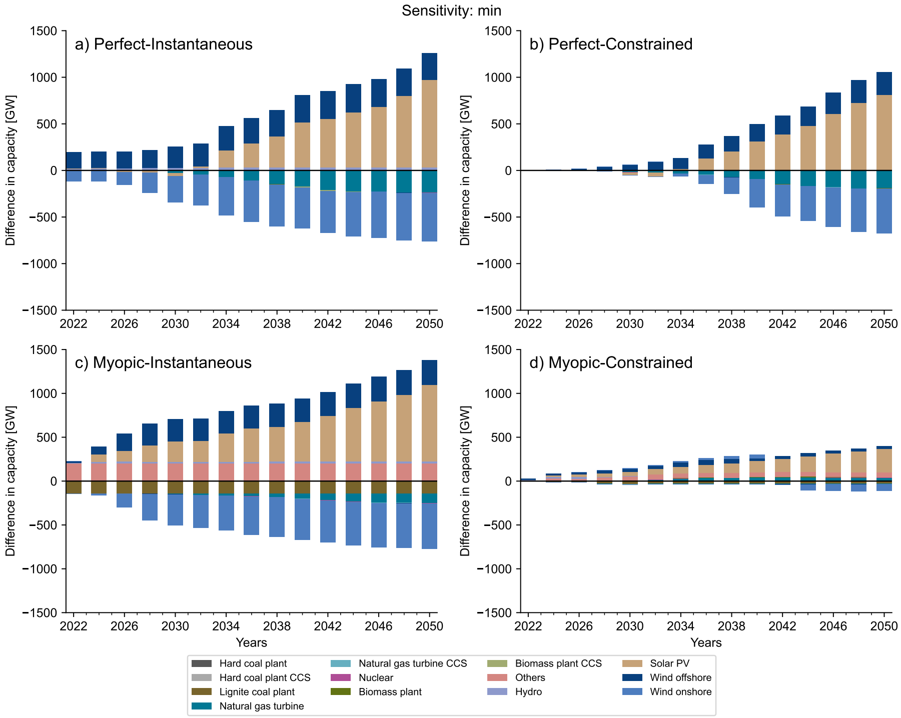


**Fig. S7** Difference in electricity generation capacities from 2022 to 2050 between minimum cost parameters and reference scenario. Comparison: Maximum total capacity in reference scenario is around 4,000 GW. Related to Figure 2.

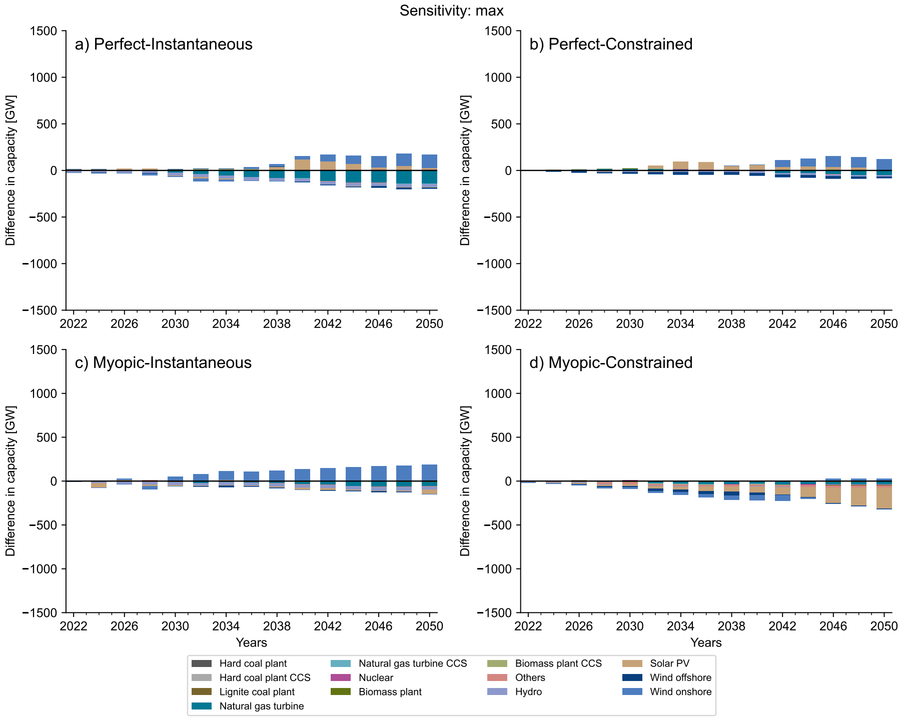


**Fig. S8** Difference in electricity generation capacities from 2022 to 2050 between maximum cost parameters and reference scenario. Comparison: Maximum total capacity in reference scenario is around 4,000 GW. Related to Figure 2.

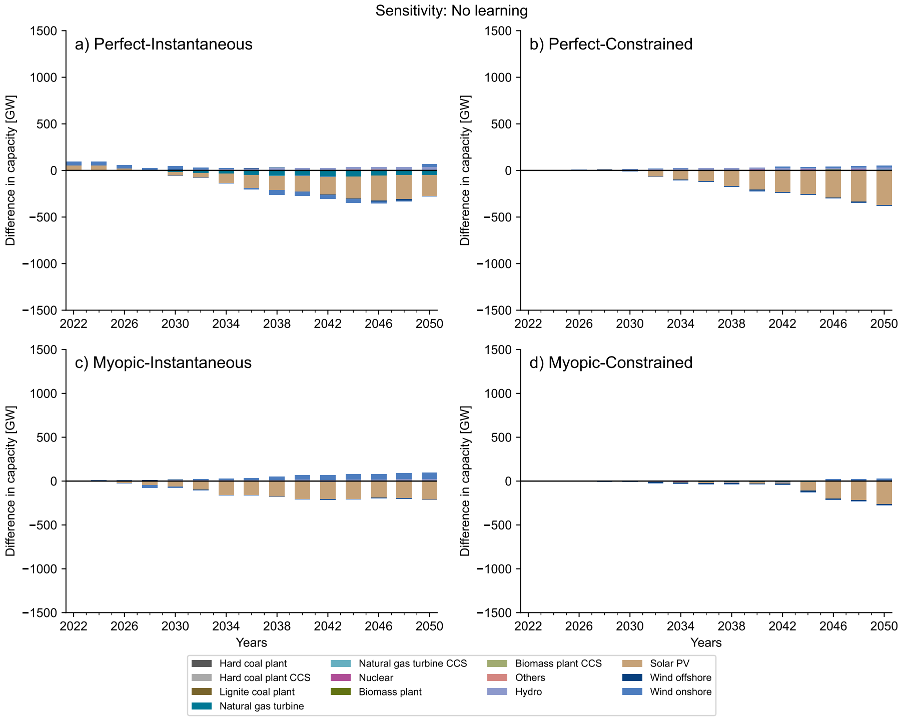


**Fig. S9** Difference in electricity generation capacities from 2022 to 2050 between cost parameters without learning and reference scenario. Comparison: Maximum total capacity in reference scenario is around 4,000 GW. Related to Figure 2.

## S1.2 Electricity and heat demand assumption

The variation in the electricity and heat demand has a small impact on cumulative emissions and electricity and heat generation capacities. In the reference scenario, we assume that the electricity and heat demand remains constant over the years (S1.2). To test this assumption, we first decrease the heat demand due to energy savings by 40% until 2050 [3] and, then, increase the electricity demand due to electrification of the transport sector by 10% until 2050 [4]. We assume linear increase or decrease over the years, starting with the reference demand in 2022. The same yearly demand time series is repeated every year but multiplied with the increase or decrease factor.

The findings of this paper are not impacted by this assumption, but the numerical values change. In detail, a decrease in heat demand results in less heat pump capacity (Fig. S12) and, therefore, lower electricity capacities (Fig. S11). The reduced stress on the energy system allows for a lower carbon budget overshoot in Myopic-Constrained. The increase in electricity demand slightly increases renewable electricity generation capacities.

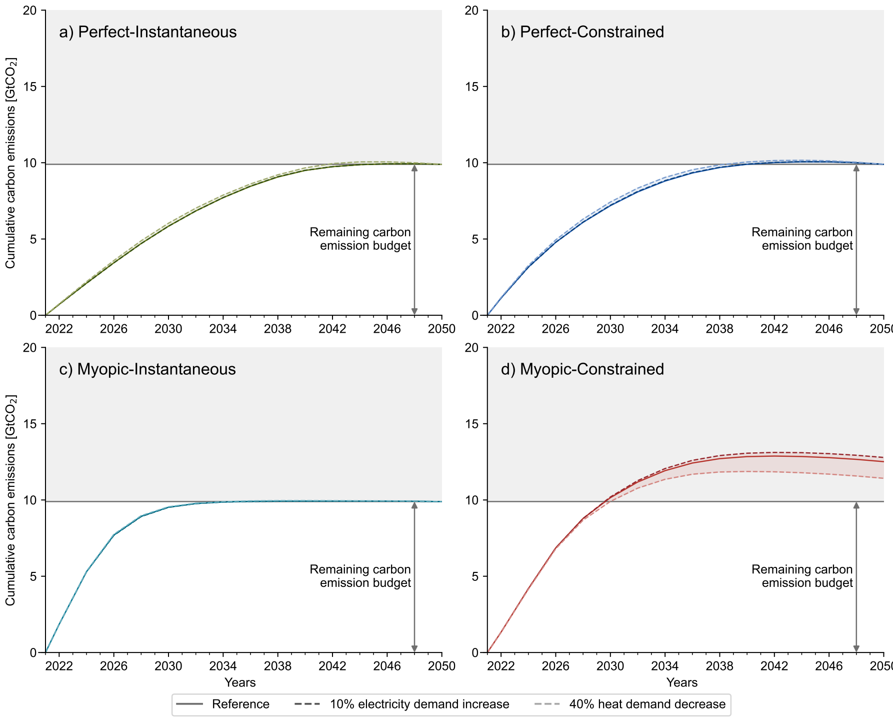


**Fig. S10** Cumulative carbon emissions for different heat demand assumptions: Reference (solid line), -40% heat demand in 2050 (dashed light line), +10% electricity demand in 2050 (dashed dark line). Related to Figure 1.

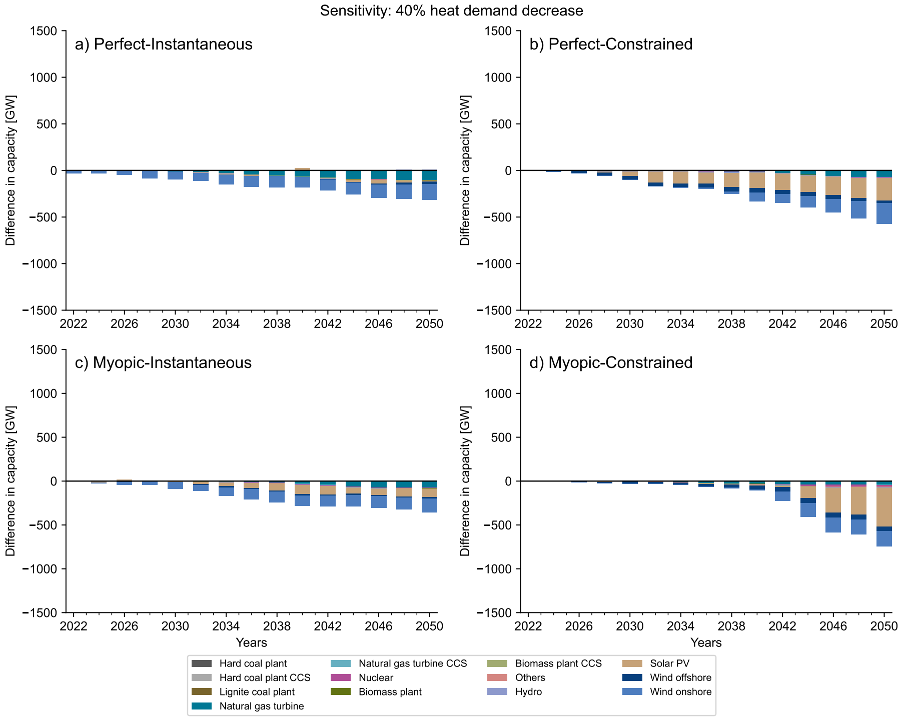


**Fig. S11** Difference in electricity generation capacities from 2022 to 2050 between low demand scenario (heat demand -40% in 2050) and reference scenario. For comparison, the maximum total capacity in reference scenario is around 4,000 GW. Related to Figure 2.

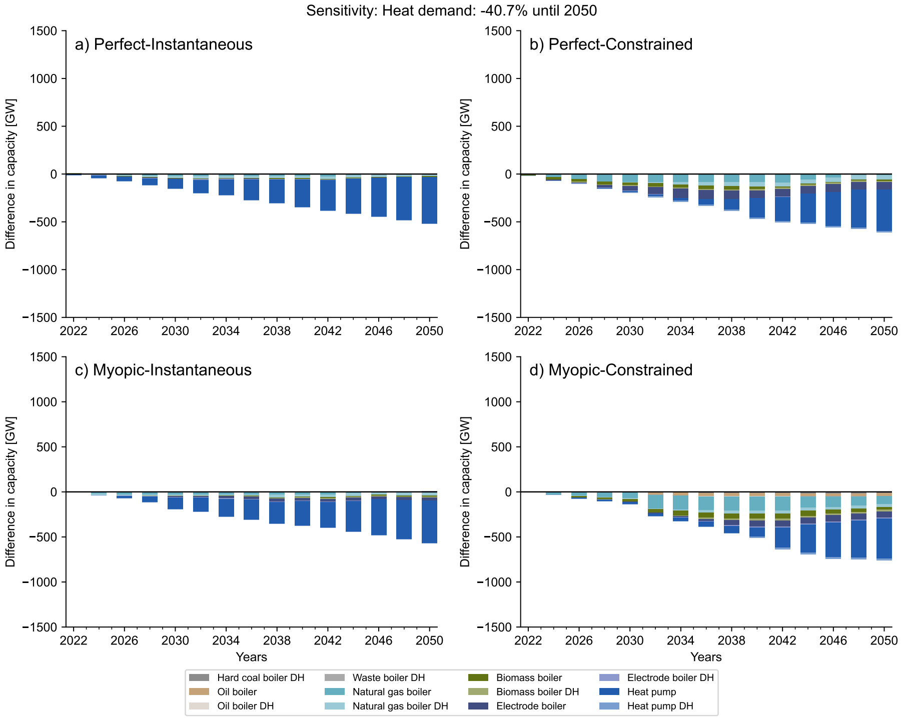


**Fig. S12** Difference in heat generation capacities from 2022 to 2050 between low demand scenario (heat demand -40% in 2050) and reference scenario. For comparison, the maximum total capacity in reference scenario is around 2,700 GW. Related to Figure 2.

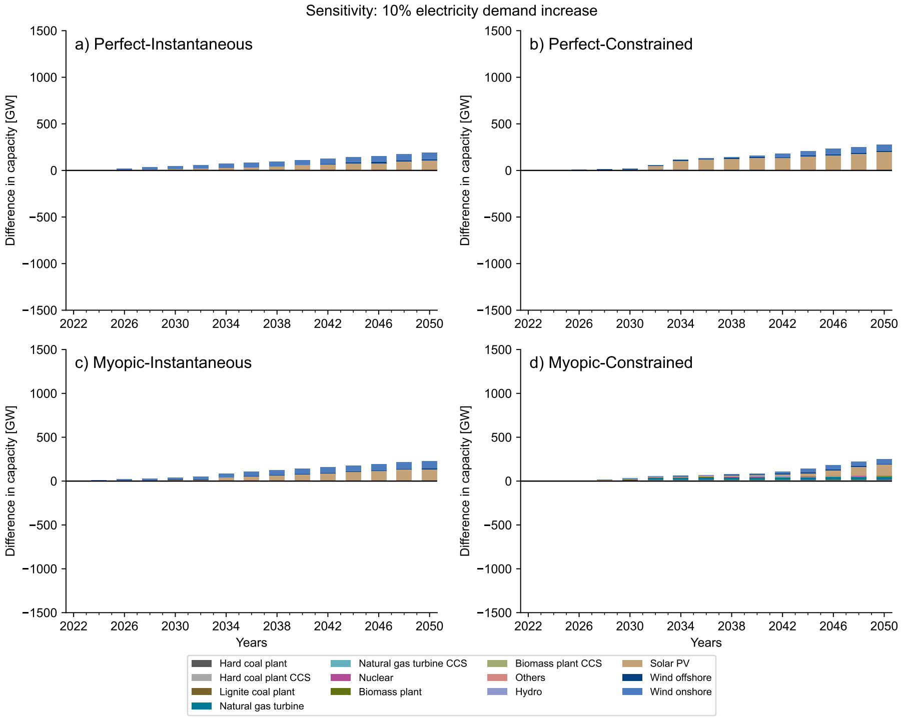


**Fig. S13** Difference in electricity generation capacities from 2022 to 2050 between high demand scenario (electricity demand +10% in 2050) and reference scenario. For comparison, the maximum total capacity in reference scenario is around 4,000 GW. Related to Figure 2.

## S1.3 Fuel price assumptions

Increasing or decreasing the fuel prices by 25% shows a limited impact on the cumulative carbon emissions, in particular only in Perfect-Instantaneous and Myopic-Constrained. Higher fuel prices incentivize early investment in renewable technologies, which reduces carbon emissions. Fuel price variations have a small impact on the cumulative net present cost (-7.8% to 7.3%).

Reduced fuel costs slightly decrease the deployment of onshore wind and solar PV, whereas increased fuel costs slightly favor the deployment of solar PV and onshore wind, albeit marginally.

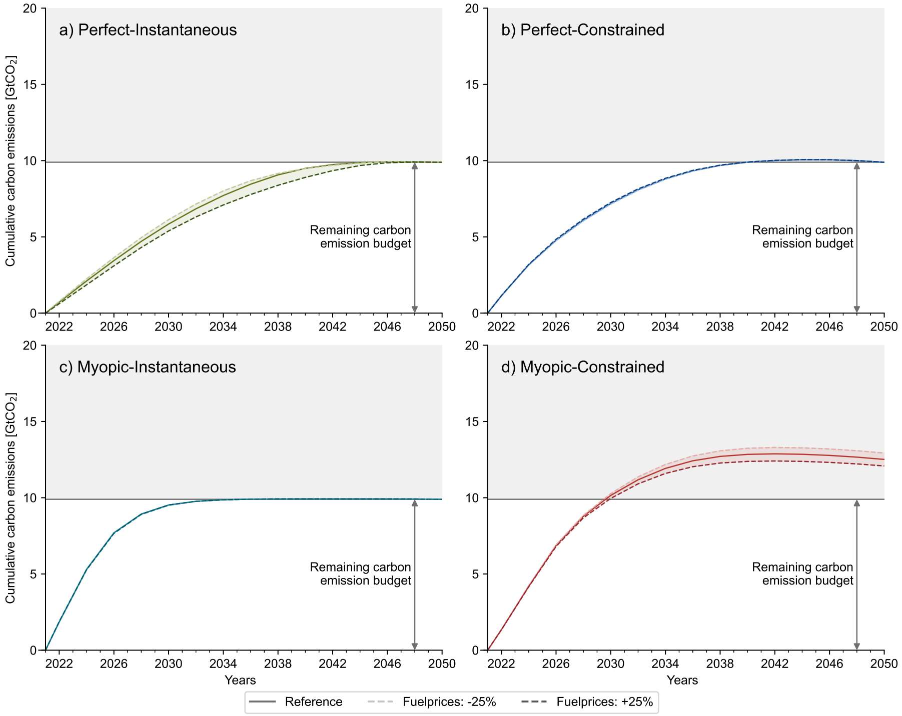


**Fig. S14** Cumulative carbon emissions for different fuel price assumptions: Reference (solid line), -25% (dashed light line), +25% (dashed dark line). Related to Figure 1.

## S1.4 Discount rate

Lower discount rates (0% or 3%) reduce the investment risk (reference value: 5%), which incentivizes an earlier and stronger deployment of renewable technologies (Figs. S16 and S17). As a result, Perfect-Instantaneous, Perfect-Constrained, and Myopic-Constrained show earlier and stronger carbon emission reductions (Fig. S15). Vice versa, a higher discount rate (9%) delays investments and leads to later carbon emission reductions (Fig. S18).

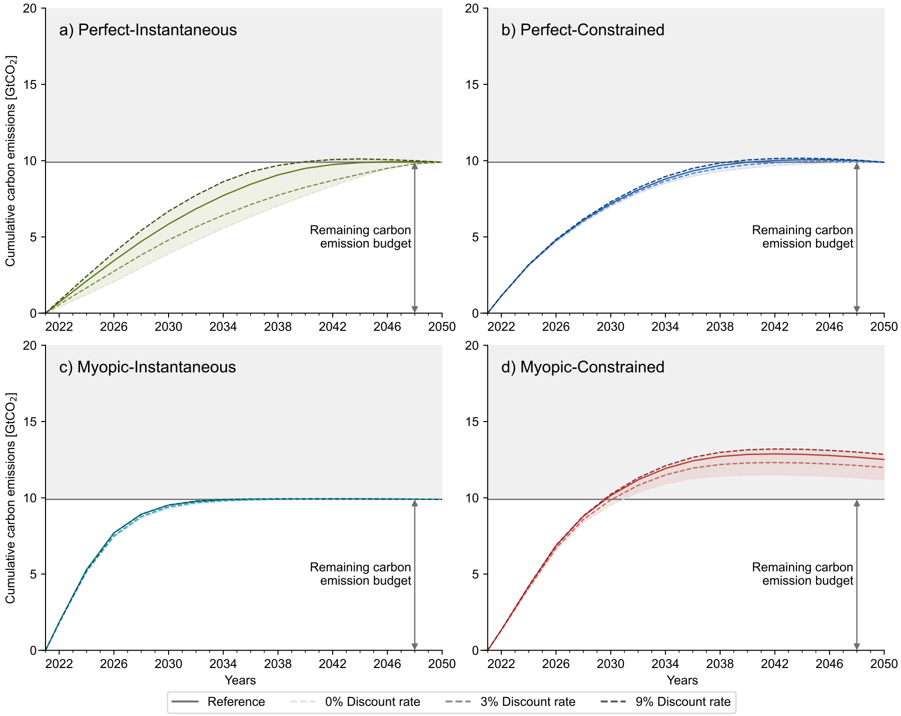


**Fig. S15** Cumulative carbon emissions for different discount rate assumptions: 0% (dashed light line), 3% (dashed medium line), 5% (reference, solid line), 9% (dashed dark line). Related to Figure 1.

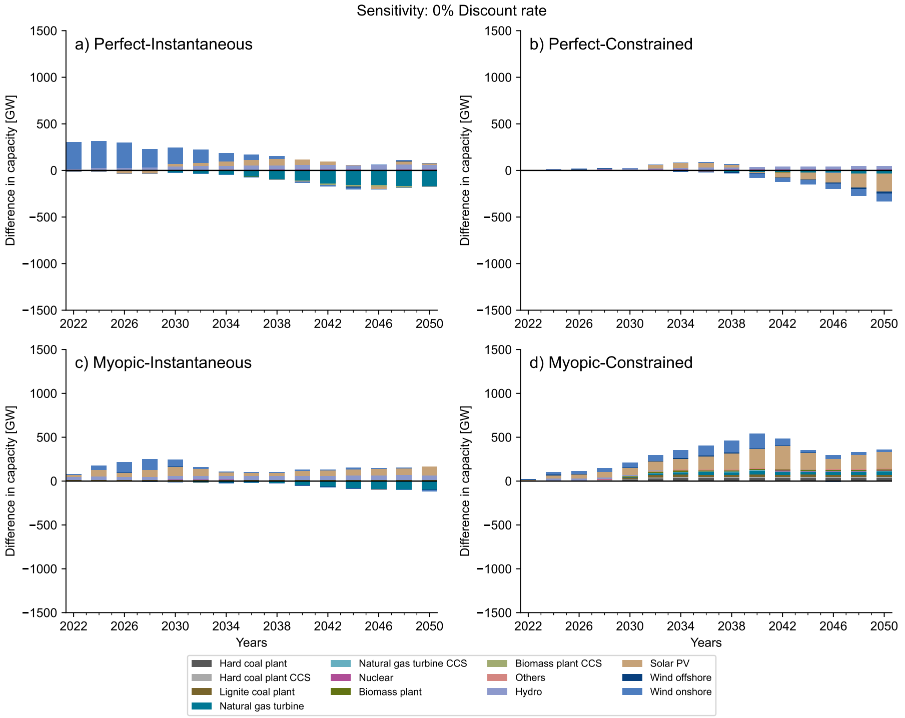


**Fig. S16** Difference in electricity generation capacities from 2022 to 2050 between 0% discount rate and reference scenario (5%). For comparison, the maximum total capacity in reference scenario is around 4,000 GW. Related to Figure 2.

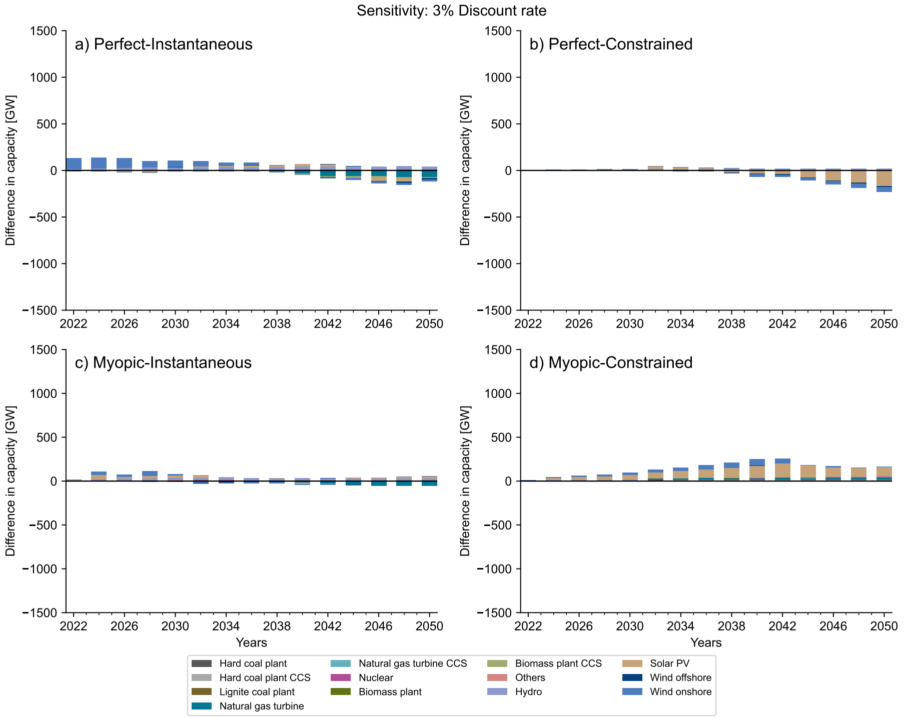


**Fig. S17** Difference in electricity generation capacities from 2022 to 2050 between 3% discount rate and reference scenario (5%). For comparison, the maximum total capacity in reference scenario is around 4,000 GW. Related to Figure 2.

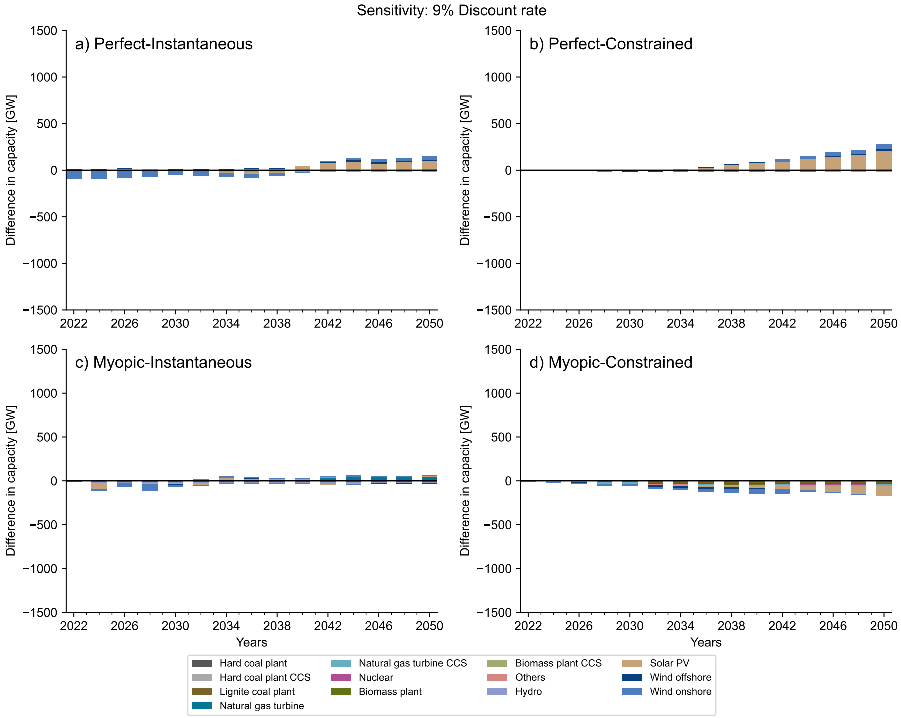


**Fig. S18** Difference in electricity generation capacities from 2022 to 2050 between 9% discount rate and reference scenario (5%). For comparison, the maximum total capacity in reference scenario is around 4,000 GW. Related to Figure 2.

## S1.5 Variable technology expansion rates

In the reference scenario, all technologies have the same technology expansion rate, namely, the maximum historical expansion rate of onshore wind (S3.11). The impact of a higher or lower expansion rate is shown in Fig. S4. Here, we characterize each technology with one of the three technology expansion rates 10% (offshore wind), 13% (onshore wind), and 29% (solar PV), based on their similarity in complexity ([5]):

1. **10%:**
   offshore wind, hard coal plant, hard coal plant with CCS, lignite coal plant, natural gas turbine, natural gas turbine with CCS, nuclear power plant, oil power plant, biomass power plant, biomass power plant with CCS, district heating grid, reservoir hydro, LNG terminal, carbon storage, power line, natural gas pipeline, carbon pipeline;
2. **13%:**
   onshore wind, run-of-river hydro, natural gas boiler DH, hard coal boiler DH, oil boiler DH, waste boiler DH, heat pump DH, electric boiler DH, biomass boiler DH;
3. **29%:**
   solar PV, natural gas boiler, heat pump, oil boiler, electric boiler, biomass boiler.

The characterization is based on perceived similarity and not on a quantitative metric. The higher expansion rate of solar PV leads to significantly higher capacities of solar PV (Fig. S20) in Myopic-Constrained. Perfect-Constrained shows similar capacities as in the reference case. As a result, Myopic-Constrained shows a significantly lower carbon budget overshoot (Fig. S19).

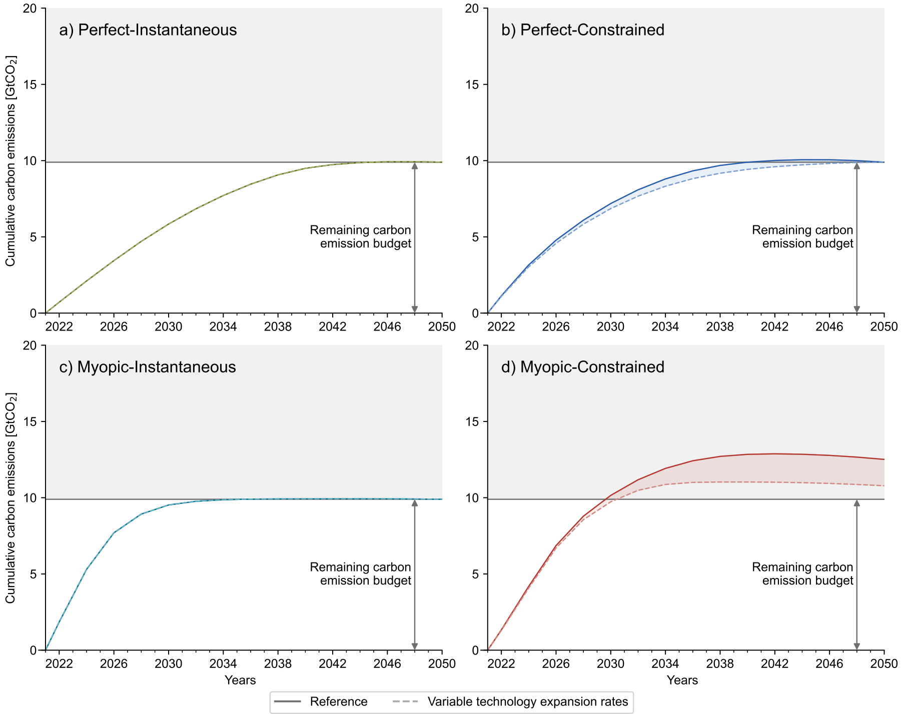


**Fig. S19** Cumulative carbon emissions for variable technology expansion rates (dashed light line). Related to Figure 1.

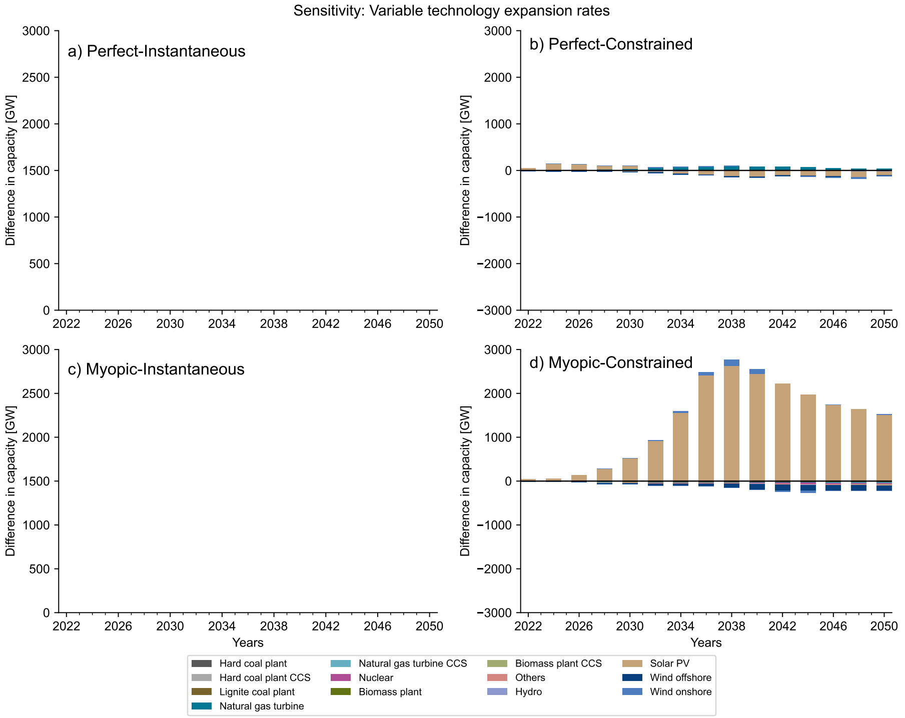


**Fig. S20** Difference in electricity generation capacities from 2022 to 2050 between variable technology expansion rates and reference scenario. For comparison, the maximum total capacity in reference scenario is around 4,000 GW. Related to Figure 2.

## S1.6 Knowledge spillover rate

Variations in the knowledge spillover rate do not impact Perfect-Instantaneous and Myopic-Instantaneous since instantaneous technology deployment is assumed. A higher knowledge spillover rate allows for increased deployment of renewable technologies in Myopic-Constrained and thus slightly decreases the carbon emission (Figs. S21 and S23). It follows vice versa for a decreased spillover rate.

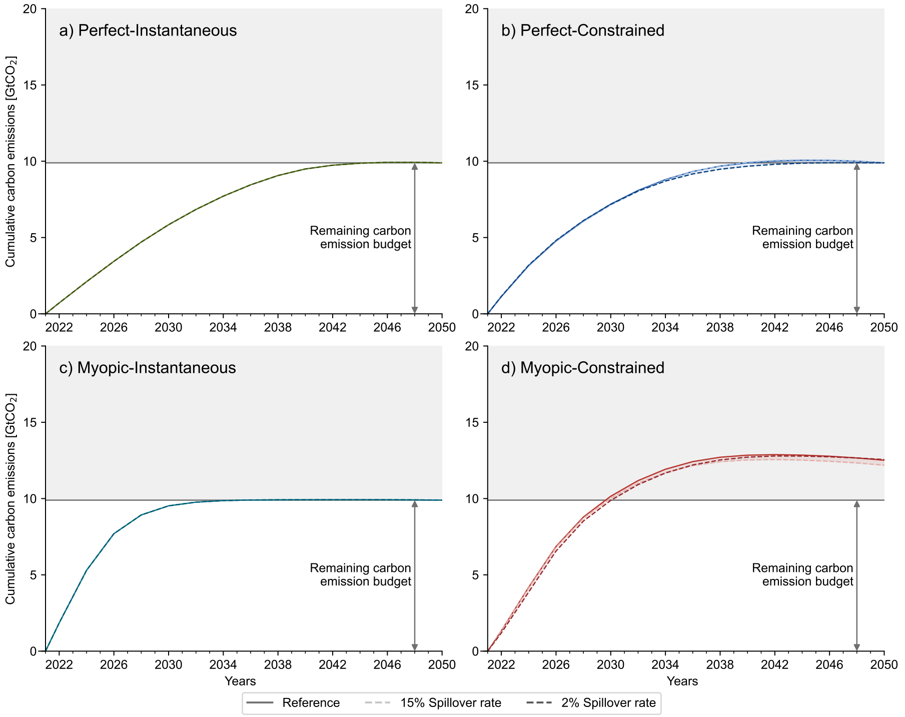


**Fig. S21** Cumulative carbon emissions for different knowledge spillover rate assumptions: Reference (7%, solid line), 2% (dashed dark line), 15% (dashed light line). Related to Figure 1.

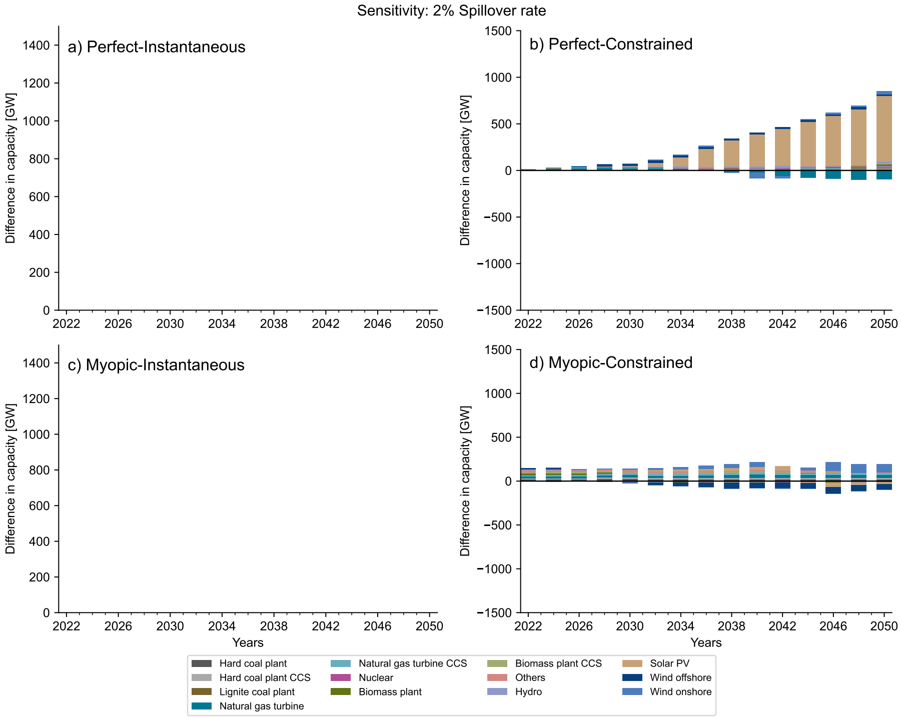


**Fig. S22** Difference in electricity generation capacities from 2022 to 2050 between reduced knowledge spillover rate (2%) and reference scenario (7%). For comparison, the maximum total capacity in reference scenario is around 4,000 GW. Related to Figure 2.

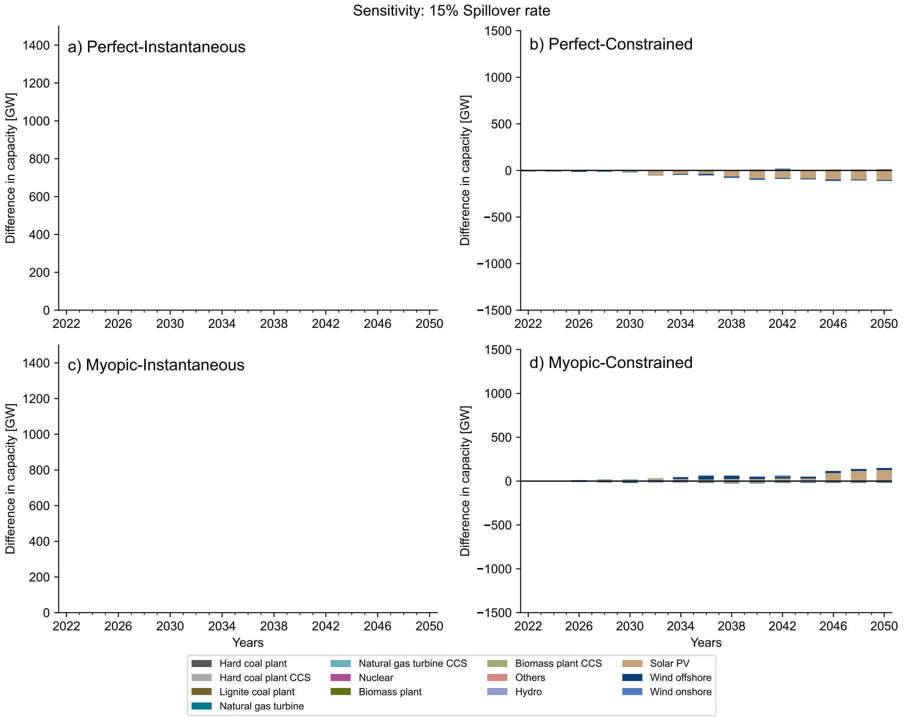


**Fig. S23** Difference in electricity generation capacities from 2022 to 2050 between increased knowledge spillover rate (15%) and reference scenario (7%). For comparison, the maximum total capacity in reference scenario is around 4,000 GW. Related to Figure 2.

## S1.7 Hydropower capacity limit

The IEA reports that the untapped economic potential of hydropower in Europe is 29% [6]. As a sensitivity analysis, we increase the capacity limit of all hydropower technologies by 29% (untapped economic potential for Europe from IEA [6], Fig. S25), 100% (Fig. S26), and 900% (Fig. S27). The assumption only has a noticeable impact on the carbon emission trajectory of Myopic-Constrained (Fig. S24). When the carbon budget is overshot, the optimizer expands all low-carbon technologies as strongly as possible, including all hydropower (Figs. S25 to S27). Myopic-Constrained can then reduce the necessary capacity additions of onshore wind and solar PV in later years. The impact on the other models is negligible.

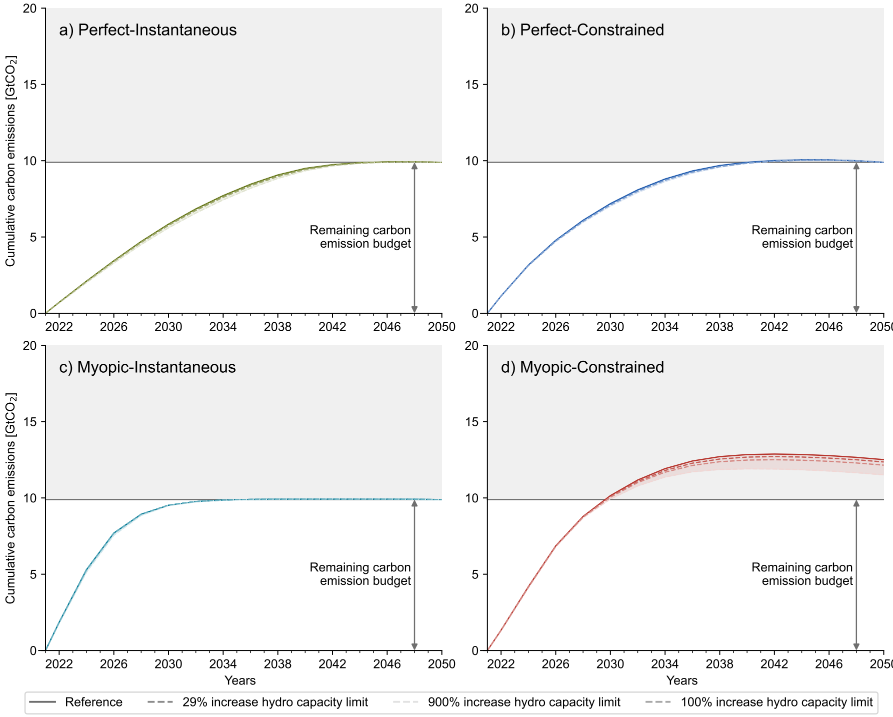


**Fig. S24** Cumulative carbon emissions for different capacity limits of hydropower: Reference (solid line), 29% increase (dashed medium light line), 100% increase (dashed light line), and 900% increase (dashed very light line). Related to Figure 1.

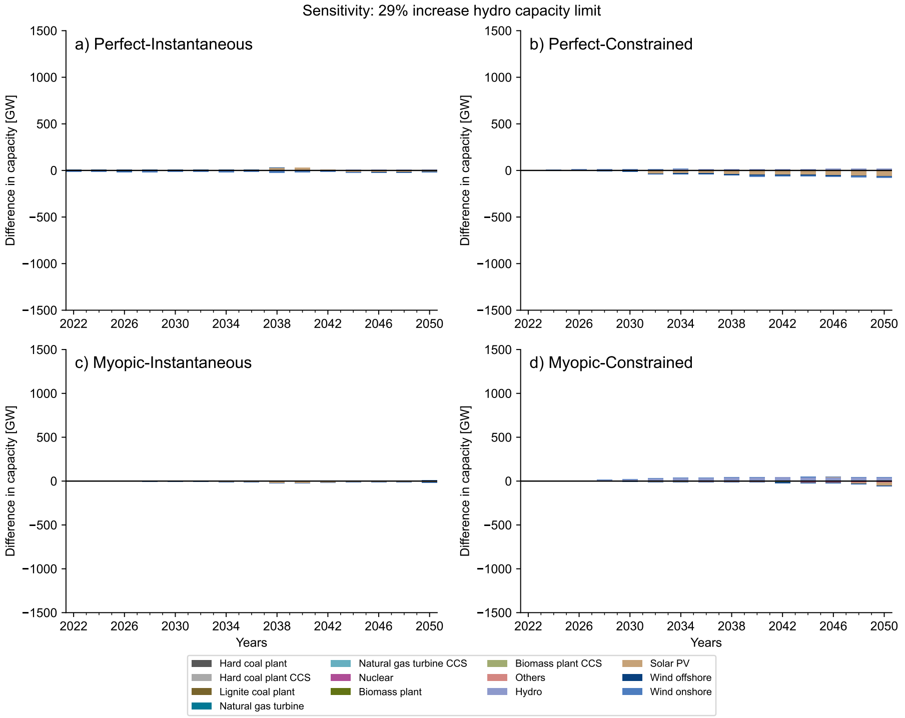


**Fig. S25** Difference in electricity generation capacities from 2022 to 2050 between 29% increase of hydropower capacity limit and reference scenario. For comparison, the maximum total capacity in reference scenario is around 4,000 GW. Related to Figure 2.

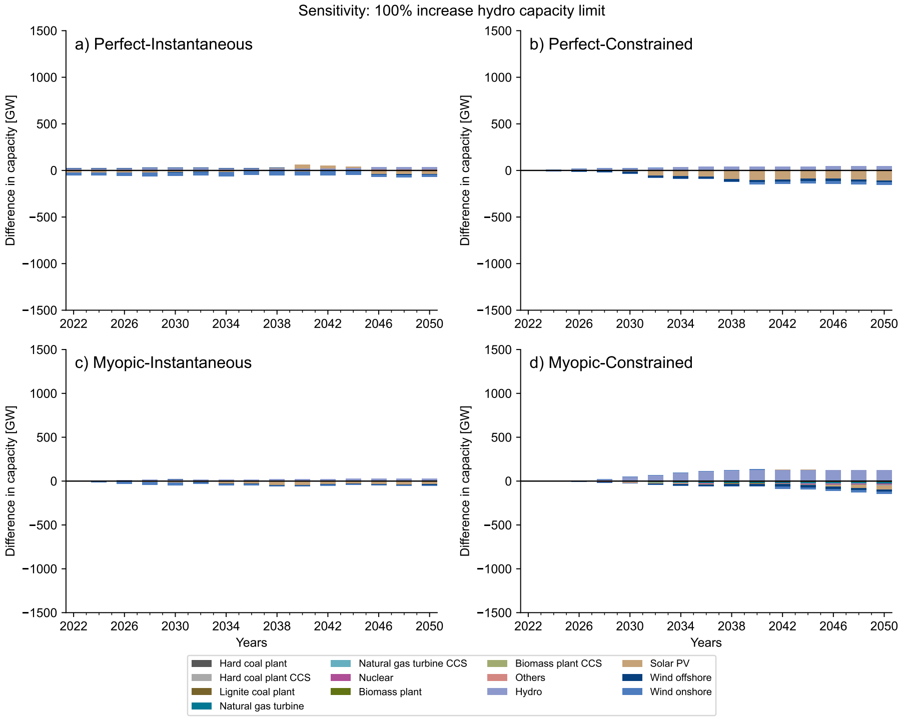


**Fig. S26** Difference in electricity generation capacities from 2022 to 2050 between 100% increase of hydropower capacity limit and reference scenario. For comparison, the mximum total capacity in reference scenario is around 4,000 GW. Related to Figure 2.

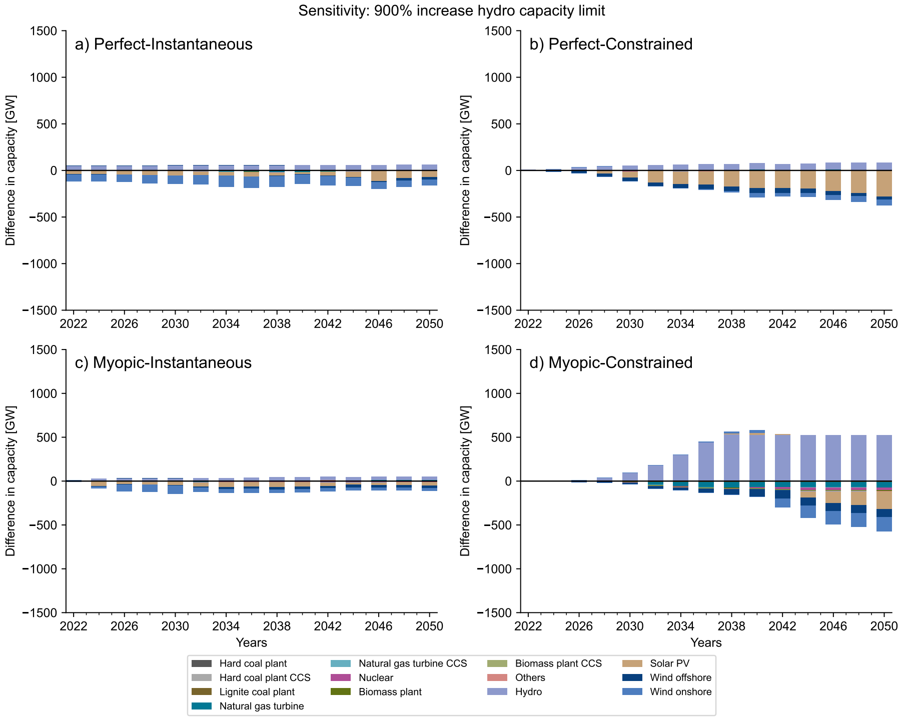


**Fig. S27** Difference in electricity generation capacities from 2022 to 2050 between 900% increase of hydropower capacity limit and reference scenario. For comparison, the maximum total capacity in reference scenario is around 4,000 GW. Related to Figure 2.

## S1.8 Carbon storage capacity limit

The assumption of the carbon storage capacity limit only has a significant impact on the carbon emission trajectory of Perfect-Instantaneous (Fig. S28). An infinite carbon storage potential allows for significant carbon emission removal, which is utilized by Perfect-Instantaneous and Myopic-Instantaneous (Table S1). Myopic-Constrained cannot expand the carbon supply chain fast enough to utilize the entire storage potential. Only Perfect-Instantaneous and Myopic-Instantaneous react strongly to an increased or decreased carbon storage capacity limit by installing less (Fig. S29) or more renewable capacity (Fig. S30). The impact of the variation in capacity limit on the electricity generation capacities in Myopic-Constrained is negligible, as the technology expansion constraint prohibits the full use of the carbon storage potential.

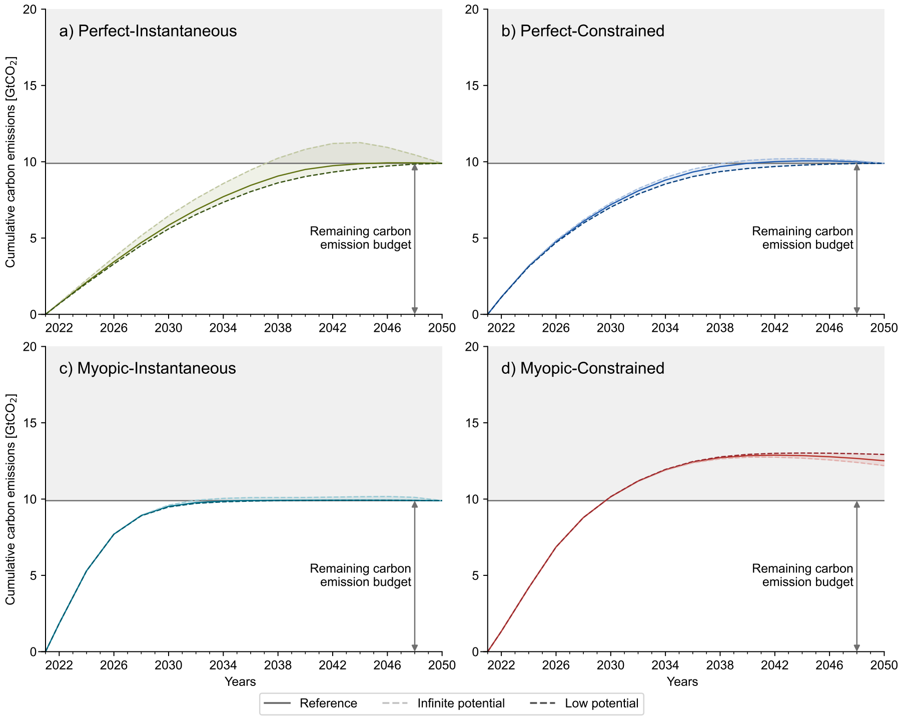


**Fig. S28** Cumulative carbon emissions for different capacity limits of carbon storage: i) reference case (solid line), ii) infinite potential (dashed light line), and iii) low potential (dashed dark line, Table S8). Related to Figure 1.

**Table S1** Maximum annual carbon removal flows for each model and carbon storage potential [$MtCO_2$] (Table S8). Related to STAR Methods.

| Model | Reduced potential max = 60.4 $MtCO_2$ | Reference potential max = 145.6 $MtCO_2$ | Infinite potential max = inf $MtCO_2$ |
|---|---|---|---|
| PI | 60.4 | 145.6 | 468.0 |
| MI | 60.4 | 145.6 | 401.6 |
| PC | 47.5 | 128.4 | 175.6 |
| MC | 42.0 | 100.1 | 132.7 |

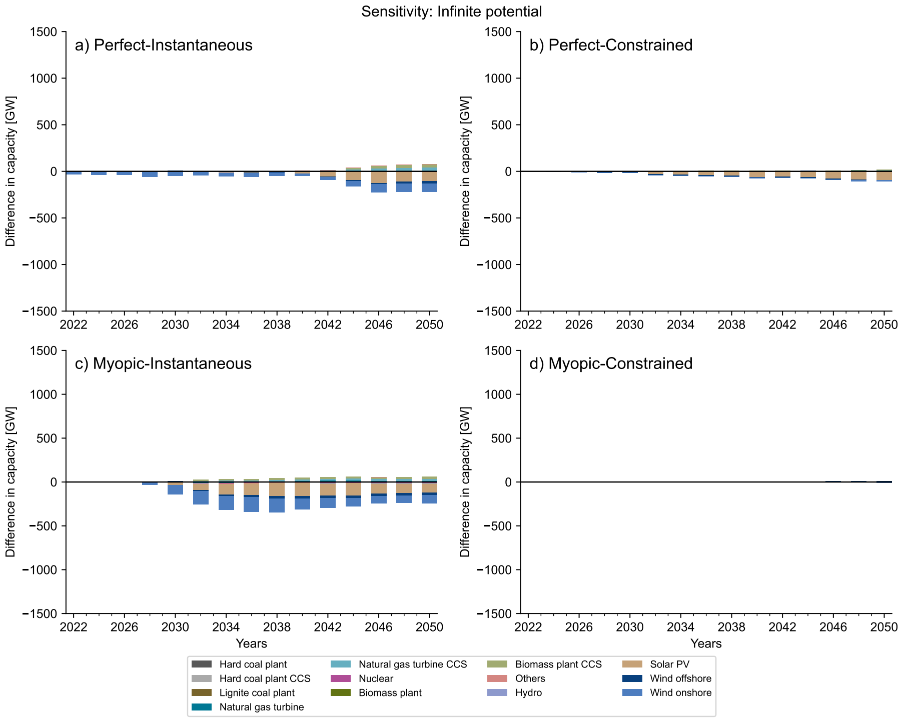


**Fig. S29** Difference in electricity generation capacities from 2022 to 2050 between infinite carbon storage potential and reference scenario. Comparison: Maximum total capacity in reference scenario is around 4,000 GW. Related to Figure 2.

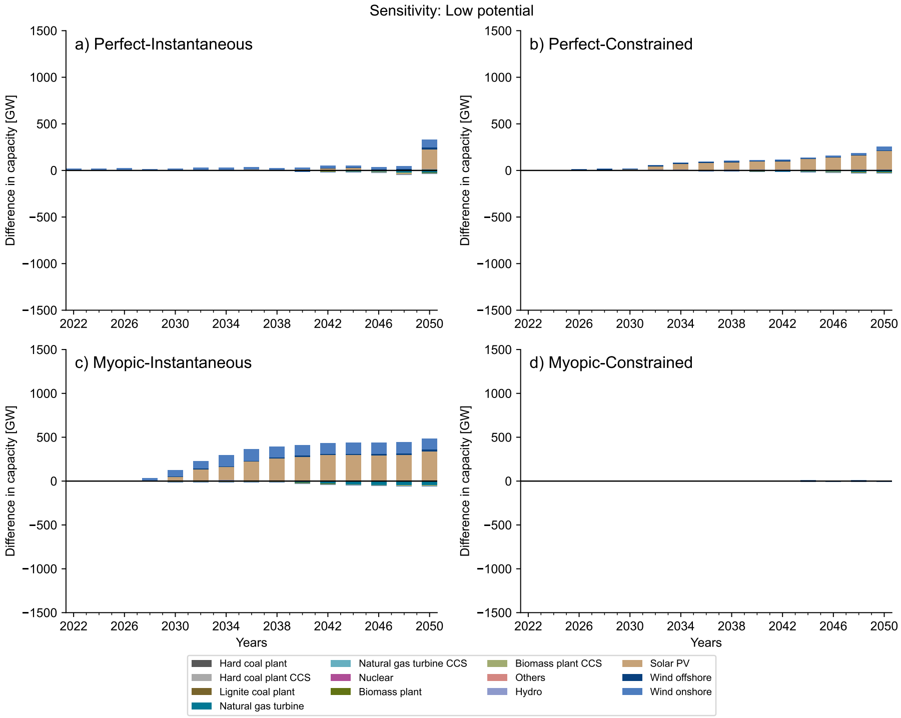


**Fig. S30** Difference in electricity generation capacities from 2022 to 2050 between low carbon storage potential and reference scenario. Comparison: Maximum total capacity in reference scenario is around 4,000 GW. Related to Figure 2.

## S1.9 Carbon budget overshoot cost

The cost of overshooting the carbon budget (overshoot cost of 5,000 EUR/t$CO_2$) is chosen to be more expensive than any technological decarbonization alternative but less expensive than electricity load shedding (load shedding cost of 100,000 EUR/MWh). Note that the units of overshoot cost and load shedding cost are not comparable; however, the emission factor of electricity would need to exceed 20 t$CO_2$/MWh to make electricity load shedding more economic than overshooting the carbon budget. The highest possible emission factor in this study is lignite-based electricity generation with an emission factor of 0.96 t$CO_2$/MWh (Tables S3 and S6). Varying the carbon budget overshoot cost in a wide range from 2,500 EUR/t$CO_2$ (-50%) to 7,500 EUR/t$CO_2$ (+50%) does not impact the numerical results in any way (Fig. S31).

Only when the overshoot cost is reduced by more than 50%, the pathways with constrained technology deployment, i.e., Myopic-Constrained and Perfect-Constrained, start showing significantly higher budget overshoots: Myopic-Constrained shows an increased overshoot from 500 EUR/t$CO_2$ (-90%) and below. Perfect-Constrained shows an increased overshoot from 250 EUR/t$CO_2$ (-95%) and below. For Myopic-Instantaneous and Perfect-Instantaneous, the carbon budget overshoot cost does not impact the results in the investigated range.

Note that the overshoot cost penalizes cumulative carbon emissions above the carbon budget and is thus not directly comparable to a carbon price (cost per unit emitted carbon) or social cost of carbon [7].

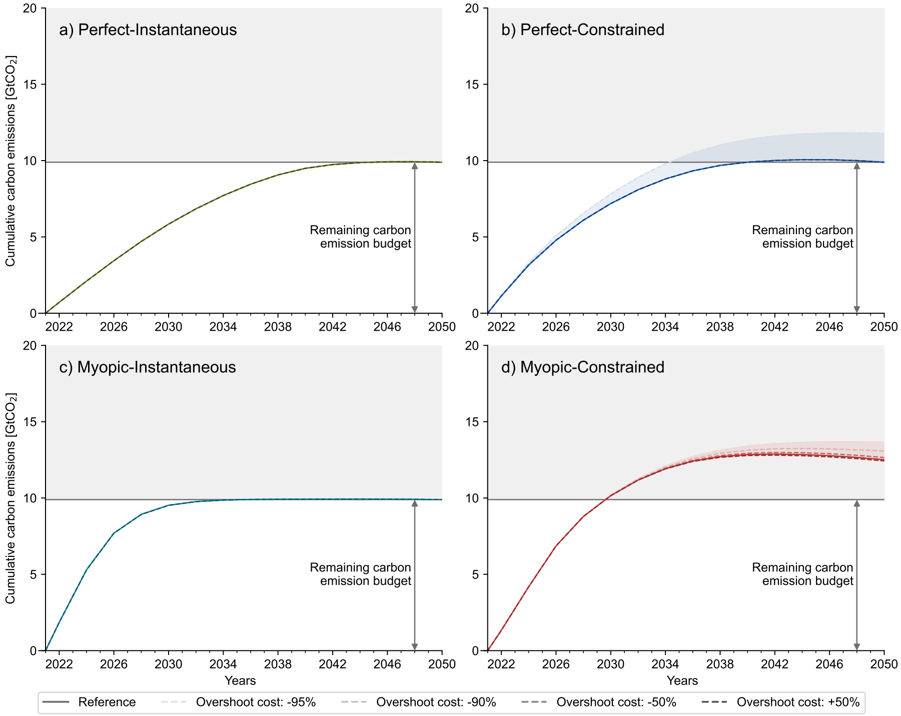


**Fig. S31** Cumulative carbon emissions for different carbon budget overshoot costs: 5000 EUR/t$CO_2$ (reference, solid line), 250 EUR/t$CO_2$ (-95%, very bright dashed line), 500 EUR/t$CO_2$ (-90%, bright dashed line), 2500 EUR/t$CO_2$ (-50%, medium dashed line), 7500 EUR/t$CO_2$ (+50%, dark dashed line). Related to Figure 1.

## S1.10 Heat supply substitution

Assuming that the entire heat supply can be substituted by other technologies (full substitution, S3.7) reduces the cumulative carbon emissions, especially in Myopic-Constrained (Fig. S32). The existing capacities of other low-carbon heating technologies, mainly heat pumps, could then be used to supply the heat demand in times of lower demand (top panel in Fig. S33), which reduces the need for utilizing fossil heating technologies. The remaining three panels in Fig. S33 show that the technologies can only supply the heat demand according to their share of total capacity. The assumption to which degree the heat supply in district heating (DH) grids can be substituted by other technologies has no discernible impact on the generated heat or cumulative carbon emissions of any model.

The assumption of full substitution of all heat supply strongly reduces the necessary heat generation capacities, especially heat pumps (Fig. S34). Full or no substitution of DH heat supply has a limited impact on heat generation capacities.

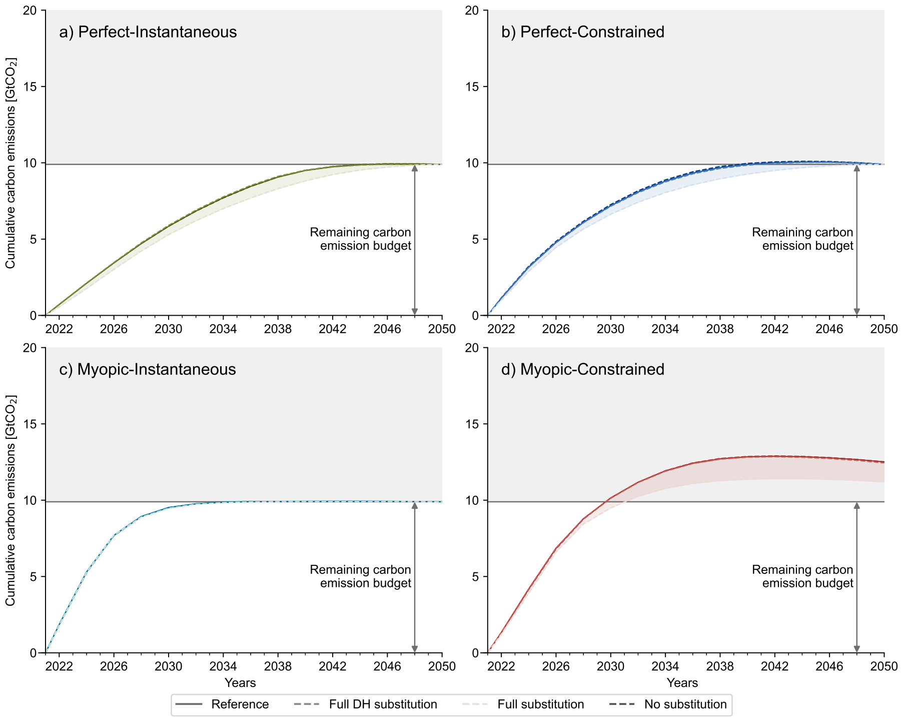


**Fig. S32** Cumulative carbon emissions for different heat supply substitution approach: Full substitution of all heat supply (dashed light line), full substitution of district heat (DH) supply (dashed medium line), mixed substitution of DH supply (reference, solid line, S3.7), no substitution (dashed dark line). Related to Figure 1.

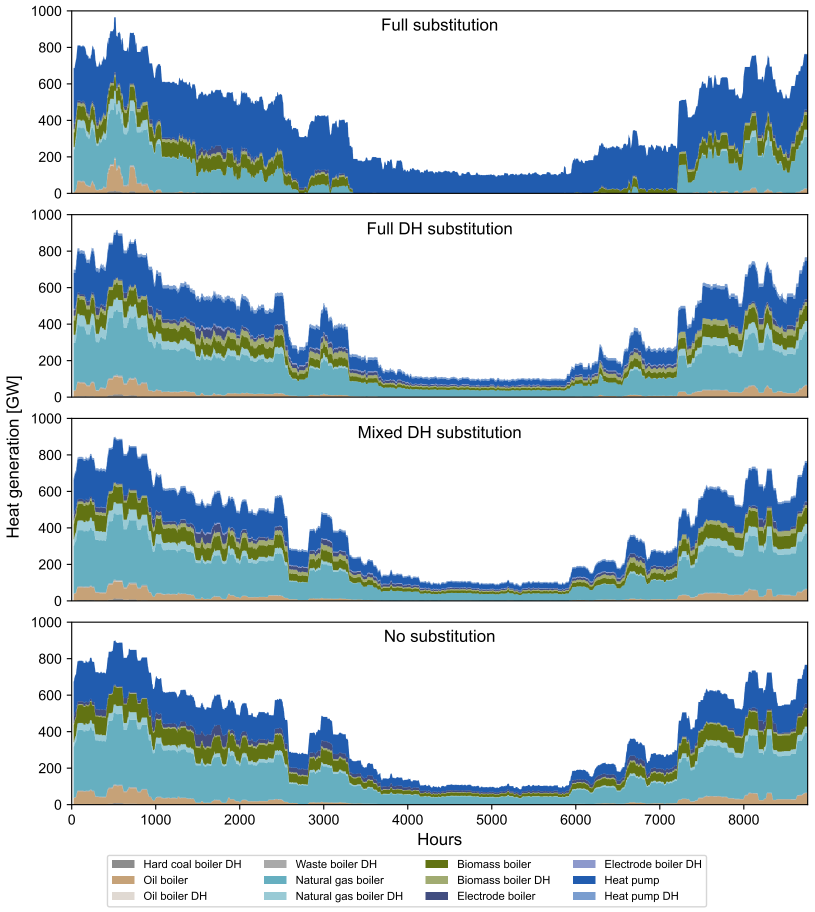


**Fig. S33** Heat supply by technology in DE in 2030 for Perfect-Constrained scenario (rolling average of 24h). Different heat supply substitution approach: Full substitution of all heat supply (top panel), full substitution of district heat (DH) supply (second panel), mixed substitution of DH supply (reference case, third panel, S3.7), no substitution (bottom panel). Related to STAR Methods.

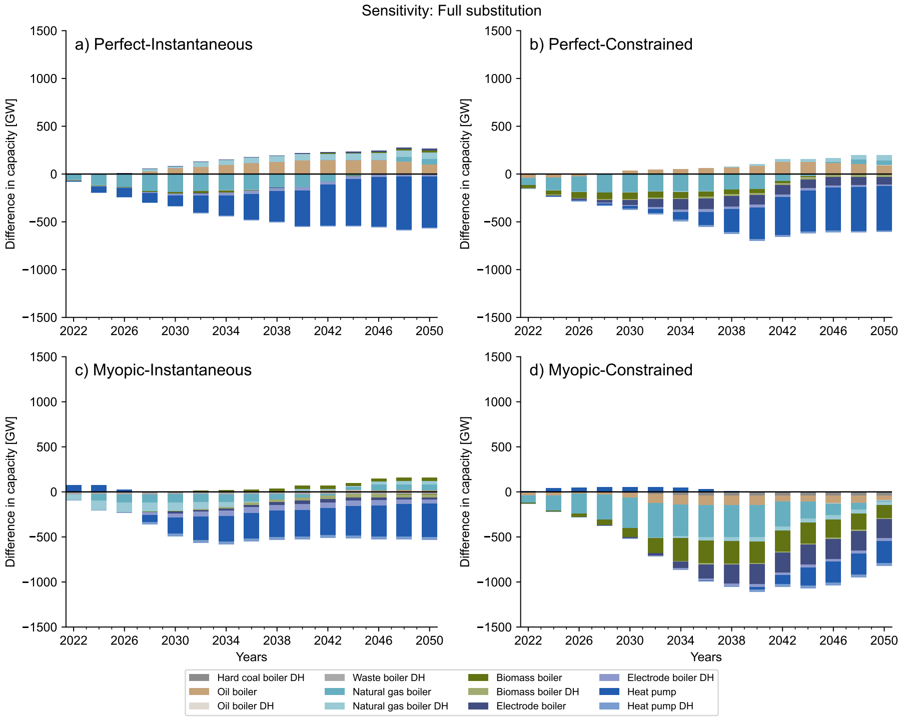


**Fig. S34** Difference in heat generation capacities from 2022 to 2050 between full substitution of all heat supply and reference scenario. Comparison: Maximum total capacity in reference scenario is around 2,700 GW. Related to Figure 2.

## S1.11 Temporal resolution

The number of operational, power-rated time steps (ts) per year is increased from 100 to 200, 500, 1000, and 2000 for the Myopic-Constrained scenario to assess the impact of the time series aggregation and representation (S2.6). The temporal resolution is not increased for the perfect foresight scenarios, Perfect-Instantaneous and Perfect-Constrained, due to limitations of computational resources. Table S2 summarizes the impact on the number of storage time steps, the cumulative carbon emissions (Fig. S35), cumulative net present cost (NPC), and solution time.

An increase of operational time steps from 100 ts to 2000 ts increases the cumulative carbon emissions by only 4.4% and the cumulative NPC by only 3.4%, while resulting in a 732% increase in solution time. The reason for the good temporal representation with only 100 representative time steps is our novel storage time step formulation (Fig. S38 [8]).

By definition, there is always a greater or equal number of storage time steps than operational time steps. A small number of operational time steps yields a large increase in storage time steps, whereas, for many operational time steps, there are almost equally as many storage time steps (Fig. S36). Thus, both the short-term (daily or weekly) and seasonal behavior of long-term storage is captured equally well by all temporal aggregation levels.

Furthermore, we can refrain from formulating typical days as is often done in energy system optimization models [9]. Thereby, the operational time steps can better capture the temporal variability of renewable generators as they are not bound by the sequence of hours within representative days.

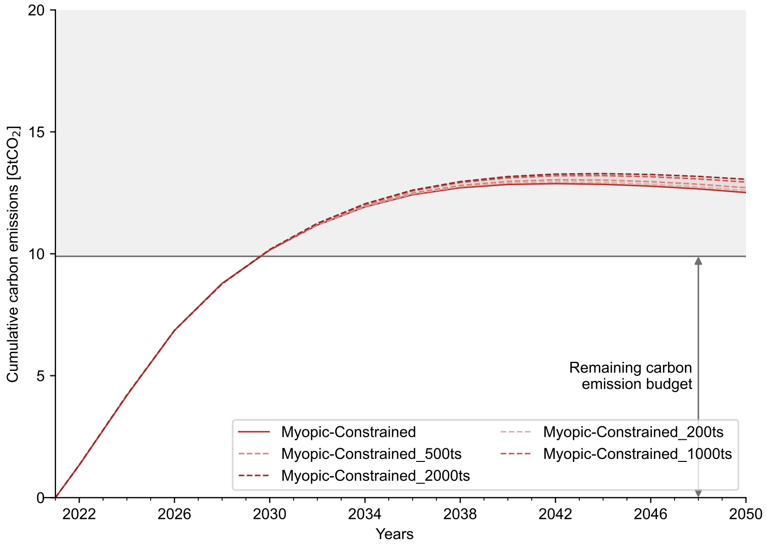


**Fig. S35** Cumulative carbon emissions of Myopic-Constrained for a varying number of time steps (ts): 100 (reference, solid line), 200 (very bright dashed line), 500 (bright dashed line), 1000 (medium bright dashed line), 2000 (dark dashed line). Related to Figure 1.

**Table S2** Impact of temporal resolution on number of storage time steps, cumulative carbon emissions, net present cost (NPC; excluding overshoot and load shedding costs), and solution time. Increase of operational time steps per year from 100 to 2000. Logarithmically extrapolating ($R^2$: 0.997) the NPC to a full time resolution (8760 time steps) indicates an error of 5.2% in the final cumulative NPC for 100 time steps. Related to STAR Methods.

| # operational time steps | # storage time steps | Final cumulative carbon emissions [$GtCO_2$] | [%] | Final Cumulative NPC [bn EUR] | [%] | Solution time spent in solver [min] | [%] |
|---|---|---|---|---|---|---|---|
| 100 | 1712 | 12.5 | 0 | 7,495 | 0 | 25.1 | 0 |
| 200 | 1819 | 12.6 | +0.6 | 7,555 | +0.8 | 34.2 | +37 |
| 500 | 1998 | 12.7 | +1.6 | 7,640 | +1.9 | 42.3 | +69 |
| 1000 | 2204 | 12.9 | +3.4 | 7,706 | +2.8 | 111.9 | +347 |
| 2000 | 2702 | 13.1 | +4.4 | 7,751 | +3.4 | 208.4 | +732 |

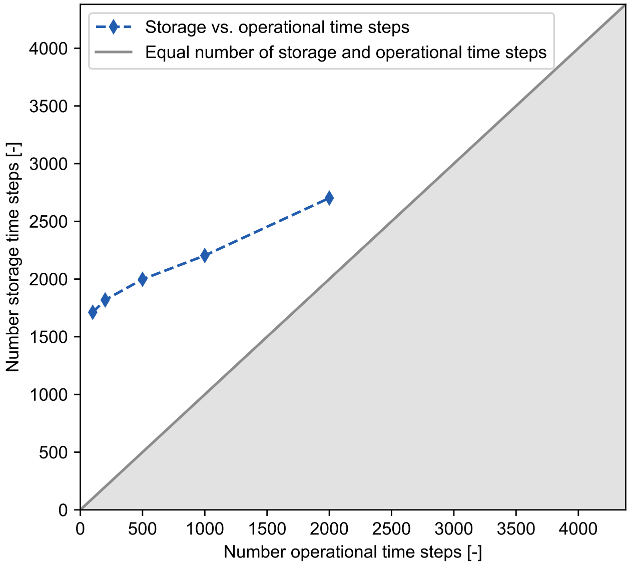


**Fig. S36** Number of storage time steps for a certain number of operational time steps (100, 200, 500, 1000, and 2000 per year). By definition of our approach, there is always a greater or equal number of storage time steps than operational time steps. Related to STAR Methods.

## S2 Nomenclature and formulation of the optimization problem in ZEN-garden

The optimization problem is implemented in the ZEN-garden (Zero-emissions Energy Networks) optimization framework, developed at the Reliability and Risk Engineering Lab at ETH Zurich. ZEN-garden optimizes the design and operation of energy system models to investigate transition pathways toward decarbonization. The optimization problem is formulated in Linopy 0.1.5 [10] and solved using the commercial solver Gurobi 9.5.1 [11]. The following chapter presents the nomenclature and formulation of the optimization model in ZEN-garden.

All input data, the source code of the optimization framework, and raw result files are accessible in the Zenodo Repository https://zenodo.org/records/11074289.

### S2.1 Nomenclature of the optimization problem

| **Sets** | **Description** |
|---|---|
| $\mathcal{T}$ | set of time steps |
| $\mathcal{T}^{\mathrm{k}}$ | set of storage level time steps |
| $\mathcal{Y}$ | set of yearly planning periods |
| $\mathcal{P}$ | set of positions (nodes and edges) |
| $\mathcal{N} \subseteq \mathcal{P}$ | set of nodes |
| $\mathcal{E} \subseteq \mathcal{P}$ | set of edges |
| $\mathcal{C}$ | set of carriers |
| $\mathcal{H}$ | set of technologies |
| $\mathcal{I} \subseteq \mathcal{H}$ | set of conversion technologies |
| $\mathcal{J} \subseteq \mathcal{H}$ | set of transport technologies |
| $\mathcal{K} \subseteq \mathcal{H}$ | set of storage technologies |

| **Indexed Sets** | **Description** |
|---|---|
| $\underline{\mathcal{E}}_n \subseteq \mathcal{E}$ | set of edges leading into a node $n \in \mathcal{N}$ |
| $\overline{\mathcal{E}}_n \subseteq \mathcal{E}$ | set of edges leading out of a node $n \in \mathcal{N}$ |
| $\mathcal{C}_h^r = \{c_h^{\mathrm{r}}\} \subseteq \mathcal{C}$ | set of reference carrier for technology $h \in \mathcal{H}$ |
| $\underline{\mathcal{C}}_i \subseteq \mathcal{C}$ | set of input carriers for conversion technology $i \in \mathcal{I}$ |
| $\overline{\mathcal{C}}_i \subseteq \mathcal{C}$ | set of output carriers for conversion technology $i \in \mathcal{I}$ |

| **Parameter (Technical)** | **Description** | **Domain** |
|---|---|---|
| $a_{c,n,t,y}$ | import availability of carrier $c$ at node $n$ and time $t$ in period $y$ | $\mathbb{R}_+$ |
| $d_{c,n,t,y}$ | demand of carrier $c$ at node $n$ and time $t$ in period $y$ | $\mathbb{R}_+$ |

| | | |
|---|---|---|
| $l_h$ | lifetime of technology $h$ | $\mathbb{N}$ |
| $m_{h,p,t,y}$ | maximum load of technology $h$ in position $p$ and time $t$ in period $y$ | [0,1] |
| $\Delta s^{\mathrm{ex}}_{h,p,y}$ | existing capacity addition of technology $h$ in position $p$ installed in period $y$ | $\mathbb{R}_+$ |
| $\Delta s^{\mathrm{ex,e}}_{k,n,y}$ | energy-rated (e) existing capacity addition of storage technology $k$ in node $n$ installed in period $y$ | $\mathbb{R}_+$ |
| $s^{\max}_{h,p,y}$ | capacity limit of technology $h$ in position $p$ in period $y$ | $\mathbb{R}_+$ |
| $s^{\max,\mathrm{e}}_{k,n,y}$ | energy-rated (e) capacity limit of storage technology $k$ in node $n$ in period $y$ | $\mathbb{R}_+$ |
| $\delta$ | knowledge depreciation rate | [0,1] |
| $\zeta_h$ | unbounded capacity addition of technology $h$ | $\mathbb{R}_+$ |
| $\eta_{i,c,t,y}$ | conversion factor between input/output carrier $c$ and reference carrier $c^{\mathrm{r}}_i$ of conversion technology $i$ and time $t$ in period $y$ | $\mathbb{R}_+$ |
| $\underline{\eta}_k, \overline{\eta}_k$ | charge and discharge efficiency of storage technology $k$ | [0,1] |
| $\vartheta_h$ | technology expansion rate of technology $h$ | $\mathbb{R}_+$ |
| $\lambda_{j,e}$ | distance of edge $e$ for transport technology $j$ | $\mathbb{R}_+$ |
| $\xi$ | unbounded market share | [0,1] |
| $\rho_j$ | loss coefficient for transport technology $j$ | $\mathbb{R}_+$ |
| $\varphi_k$ | self-discharge rate of storage technology $k$ | [0,1] |
| $\omega$ | knowledge spillover rate | [0,1] |

| **Parameter (Economic)** | **Description** | **Domain** |
|---|---|---|
| $r$ | discount rate | [0,1] |
| $u_{c,n,t,y}$ | import price of carrier $c$ at node $n$ and time $t$ in period $y$ | $\mathbb{R}_+$ |
| $\alpha_{h,y}$ | specific capital expenditure of technology $h$ in period $y$ | $\mathbb{R}_+$ |
| $\alpha^{\mathrm{e}}_{k,y}$ | specific energy-rated (e) capital expenditure of storage technology $k$ in period $y$ | $\mathbb{R}_+$ |
| $\beta_{h,y}$ | specific variable operational expenditure of technology $h$ in period $y$ | $\mathbb{R}_+$ |
| $\gamma_{h,y}$ | specific fixed operational expenditure of technology $h$ in period $y$ | $\mathbb{R}_+$ |
| $\gamma^{\mathrm{e}}_{k,y}$ | specific energy-rated (e) fixed operational expenditure of storage technology $k$ in period $y$ | $\mathbb{R}_+$ |
| $\mu$ | carbon price | $\mathbb{R}_+$ |
| $\mu^{\mathrm{o}}$ | carbon overshoot price | $\mathbb{R}_+$ |
| $\nu_c$ | demand shedding price of carrier $c$ | $\mathbb{R}_+$ |

| Parameter (General) | Description | Domain |
|---|---|---|
| $\Delta^{\mathrm{y}}$ | interval between periods $y$ and $y+1$ | $\mathbb{N}$ |
| $\epsilon_h$ | carbon intensity of technology $h$ | $\mathbb{R}_+$ |
| $\epsilon_c$ | carbon intensity of carrier $c$ | $\mathbb{R}_+$ |
| $e_y$ | annual carbon emission limit in period $y$ | $\mathbb{R}_+$ |
| $e^{\mathrm{b}}$ | cumulative carbon emission budget | $\mathbb{R}_+$ |
| $\tau_t$ | duration of time step $t$ | $\mathbb{N}$ |
| $\tau^{\mathrm{k}}_{t^{\mathrm{k}}}$ | duration of storage level time step $t^{\mathrm{k}}$ | $\mathbb{N}$ |

| Variable (Technical) | Description | Domain |
|---|---|---|
| $D_{c,n,t,y}$ | Shed demand of carrier $c$ at node $n$ and time $t$ in period $y$ | $\mathbb{R}_+$ |
| $F_{j,e,t,y}$ | flow of reference carrier $c^{\mathrm{r}}_j$ through transport technology $j$ at edge $e$ and time $t$ in period $y$ | $\mathbb{R}_+$ |
| $F^{\mathrm{l}}_{j,e,t,y}$ | carrier loss of reference carrier $c^{\mathrm{r}}_j$ in transport technology $k$ at edge $e$ and time $t$ in period $y$ | $\mathbb{R}_+$ |
| $G^{\mathrm{r}}_{i,n,t,y}$ | flow of reference carrier $c^{\mathrm{r}}_i$ from conversion technology $i$ at node $n$ and time $t$ in period $y$ | $\mathbb{R}_+$ |
| $\underline{G}_{c,i,n,t,y}$ | input flow of carrier $c \in \underline{\mathcal{C}}_i$ into conversion technology $i$ at node $n$ and time $t$ in period $y$ | $\mathbb{R}_+$ |
| $\overline{G}_{c,i,n,t,y}$ | output flow of carrier $c \in \overline{\mathcal{C}}_i$ from conversion technology $i$ at node $n$ and time $t$ in period $y$ | $\mathbb{R}_+$ |
| $\underline{H}_{k,n,t,y}$ | charge flow of reference carrier $c^{\mathrm{r}}_k$ into storage technology $k$ at node $n$ and time $t$ in period $y$ | $\mathbb{R}_+$ |
| $\overline{H}_{k,n,t,y}$ | discharge flow of reference carrier $c^{\mathrm{r}}_k$ from storage technology $k$ at node $n$ and time $t$ in period $y$ | $\mathbb{R}_+$ |
| $K_{h,p,y}$ | capacity knowledge of technology $h$ at position $p$ and period $y$ | $\mathbb{R}_+$ |
| $K^{\mathrm{e}}_{k,n,y}$ | energy-rated (e) capacity knowledge of storage technology $k$ at node $n$ and period $y$ | $\mathbb{R}_+$ |
| $L_{k,n,t^{\mathrm{k}},y}$ | charge level of storage technology $k$ at node $n$ and storage level time $t^{\mathrm{k}}$ in period $y$ | $\mathbb{R}_+$ |
| $S_{h,p,y}$ | capacity of technology $h$ at position $p$ and period $y$ | $\mathbb{R}_+$ |
| $S^{\mathrm{e}}_{k,n,y}$ | energy-rated (e) capacity of storage technology $k$ at node $n$ and period $y$ | $\mathbb{R}_+$ |
| $\Delta S_{h,p,y}$ | capacity addition of technology $h$ at position $p$ and period $y$ | $\mathbb{R}_+$ |
| $\Delta S^{\mathrm{e}}_{k,n,y}$ | energy-rated (e) capacity addition of storage technology $k$ at node $n$ and period $y$ | $\mathbb{R}_+$ |

| | | |
|---|---|---|
| $U_{c,n,t,y}$ | import of carrier $c$ at node $n$ and time $t$ in period $y$ | $\mathbb{R}_+$ |

| **Variable (Economic)** | **Description** | **Domain** |
|---|---|---|
| $A_{h,p,y}$ | annual capital expenditure for technology $h$ in position $p$ in period $y$ | $\mathbb{R}_+$ |
| $CAPEX_y$ | total system capital expenditure in period $y$ | $\mathbb{R}_+$ |
| $OPEX_y$ | total system operational expenditure in period $y$ | $\mathbb{R}_+$ |
| $NPC_y$ | net present cost of the system in period $y$ | $\mathbb{R}_+$ |

| **Variable (General)** | **Description** | **Domain** |
|---|---|---|
| $E_y$ | annual system carbon emissions | $\mathbb{R}_+$ |
| $E_y^{\mathrm{c}}$ | cumulative system carbon emissions over time until $y$ | $\mathbb{R}_+$ |
| $E_y^{\mathrm{o}}$ | annual system carbon emission overshoot | $\mathbb{R}_+$ |

### S2.2 Objective function

The objective of the optimization problem, $J$, is to minimize the net present cost, $NPC_y$, over the entire planning horizon $y \in \mathcal{Y}$. $NPC_y$ includes the annual capital expenditures, $CAPEX_y$, and annual operational expenditures, $OPEX_y$, and accounts for the interval between planning periods $\Delta^{\mathrm{y}}$. The last period of the planning horizon $Y = \max(y)$ is only counted as a single year since we assume that the optimization is only conducted until the end of the first year of the last planning period. The future cash flows are discounted with a constant discount rate of $r = 6\%$ (S1.4):

$$
\begin{aligned}
J &= \sum_{y\in\mathcal{Y}} NPC_y \qquad\qquad \text{(S1)}\\
&= \sum_{y\in\mathcal{Y}}^{Y-1} \sum_{\tilde{y}=0}^{\Delta^{\mathrm{y}}-1} \left(\frac{1}{1+r}\right)^{\Delta^{\mathrm{y}}(y-y_0)+\tilde{y}} \left(CAPEX_y + OPEX_y\right) + \\
&\left(\frac{1}{1+r}\right)^{\Delta^{\mathrm{y}}(Y-y_0)} \left(CAPEX_Y + OPEX_Y\right).
\end{aligned}
$$

$CAPEX_y$ accounts for the annual cash flows due to capacity investments in technologies. Each technology $h \in \mathcal{H}$ is either a conversion technology $i \in \mathcal{I} \subseteq \mathcal{H}$, a transport technology $j \in \mathcal{J} \subseteq \mathcal{H}$ or a storage technology $k \in \mathcal{K} \subseteq \mathcal{H}$. For the sake of simplicity, we index those variables and parameters that apply to all technology types with $h$. Conversion and storage technologies are installed and operated on nodes $n \in \mathcal{N}$, and transport technologies are installed and operated on edges $e \in \mathcal{E}$. We summarize nodes and edges to positions $p \in \mathcal{P} = \mathcal{N} \cup \mathcal{E}$.

The total investment cost for each conversion technology $i \in \mathcal{I}$ is calculated as the product of the unit cost of capital investment $\alpha_{i,y}$ and the capacity addition $\Delta S_{i,n,y}$ on each node $n \in \mathcal{N}$. Similarly, for each transport technology $j \in \mathcal{J}$, the total investment cost is the product of the unit cost of capital investment per distance $\alpha_{j,y}$, the capacity addition $\Delta S_{j,e,y}$ and the transport distance $\lambda_{j,e}$ of the corresponding edge $e \in \mathcal{E}$. Last, the total investment cost for each storage technology $k \in \mathcal{K}$ is the product of the unit cost of capital investment and the capacity addition for both the power-rated capacity ($\alpha_{k,y}$ and $\Delta S_{k,n,y}$) and the energy-rated capacity ($\alpha^{\mathrm{e}}_{k,y}$ and $\Delta S^{\mathrm{e}}_{k,n,y}$).

To annualize the investment, the total investment cost is multiplied by the annuity factor $f_h$ with the technology lifetime $l_h$:

$$
f_h = \frac{(1+r)^{l_h}\, r}{(1+r)^{l_h} - 1}. \qquad \text{(S2)}
$$

The annual cash flows accrue over $l_h$. For existing capacity additions $\Delta s^{\mathrm{ex}}_{h,p,y}$ that were installed before $y_0$, we assume that they cost the unit cost in the first investment period $\alpha_{h,y_0}$. The annual capital expenditure $A_{t,p,y}$ for each

technology $h \in \mathcal{H}$ in the corresponding position $p \in \mathcal{P}$ in period $y \in \mathcal{Y}$ is computed as:

$$A_{h,p,y} = f_h \left( \sum_{\tilde{y}=\max(y_0, y-\lceil l_h/\Delta^{\mathrm{y}} \rceil+1)}^{y} \alpha_{h,\tilde{y}} \Delta S_{h,p,\tilde{y}} + \sum_{\hat{y}=\psi(y-\lceil l_h/\Delta^{\mathrm{y}} \rceil+1)}^{\psi(y_0-1)} \alpha_{h,y_0} \Delta s^{\mathrm{ex}}_{h,p,\hat{y}} \right), \tag{S3}$$

where $\lceil \cdot \rceil$ is the ceiling function and $\psi(y)$ is a function that maps the planning period $y$ (0, 1, 2, ...) to an actual year (2022, 2024, 2026, ...). The ceiling function and min function determine whether a capacity that was previously installed is still commissioned. Capacity additions that have exceeded their lifetime do not contribute to $A_{h,p,y}$ or the total capacity. For the sake of conciseness, we omit to restate Eq. (S3) for energy-rated storage capacities.

$CAPEX_y$ then follows as:

$$CAPEX_y = \sum_{h \in \mathcal{H}} \sum_{p \in \mathcal{P}} A_{h,p,y} + \sum_{k \in \mathcal{K}} \sum_{n \in \mathcal{N}} A^{\mathrm{e}}_{k,n,y}. \tag{S4}$$

The annual operational expenditure $OPEX_y$ consists of four terms: i) variable operational and maintenance costs of the technologies, $OPEX^{\mathrm{v}}_y$, ii) fixed operational and maintenance costs of the technologies, $OPEX^{\mathrm{f}}_y$, iii) cost of importing carriers, $OPEX^{\mathrm{i}}_y$, and iv) the cost of carbon emissions, $OPEX^{\mathrm{c}}_y$:

$$OPEX_y = OPEX^{\mathrm{v}}_y + OPEX^{\mathrm{f}}_y + OPEX^{\mathrm{i}}_y + OPEX^{\mathrm{c}}_y. \tag{S5}$$

$OPEX^{\mathrm{v}}_y$ is the product of the specific variable operational expenditure $\beta_{h,y}$ and the reference flows for each technology, calculated for the entire year with the time step duration $\tau_t$ and summed over all technologies and positions. The reference flows for conversion technologies are $G^{\mathrm{r}}_{i,n,t,y}$, for transport technologies $F_{j,e,t,y}$, and for storage technologies $\underline{H}_{k,n,t,y}$ and $\overline{H}_{k,n,t,y}$:

$$OPEX^{\mathrm{v}}_y = \sum_{t \in \mathcal{T}} \tau_t \Bigg( \sum_{i \in \mathcal{I}} \sum_{n \in \mathcal{N}} \beta_{i,y} G^{\mathrm{r}}_{i,n,t,y} + \sum_{j \in \mathcal{J}} \sum_{e \in \mathcal{E}} \beta_{j,y} F_{j,e,t,y} + \sum_{k \in \mathcal{K}} \sum_{n \in \mathcal{N}} \beta_{k,y} \left( \underline{H}_{k,n,t,y} + \overline{H}_{k,n,t,y} \right) \Bigg). \tag{S6}$$

$OPEX_y^{\mathrm{f}}$ is the product of the specific fixed operational expenditure, $\gamma_{h,y}$, and the capacity, $S_{h,p,y}$, summed over all technologies and positions:

$$OPEX_y^{\mathrm{f}} = \sum_{h\in\mathcal{H}}\sum_{p\in\mathcal{P}}\gamma_{h,y}S_{h,p,y} + \sum_{k\in\mathcal{K}}\sum_{n\in\mathcal{N}}\gamma_{k,y}^{\mathrm{e}}S_{k,n,y}^{\mathrm{e}}. \tag{S7}$$

$OPEX_y^{\mathrm{i}}$ is composed of a term attributed to the imported quantity of all carriers $c \in \mathcal{C}$, $U_{c,n,t,y}$, with the import price, $u_{c,n,t,y}$, and one term for the shed demand of all carriers, $D_{c,n,t,y}$, with the demand shedding price, $\nu_c$:

$$OPEX_y^{\mathrm{i}} = \sum_{c\in\mathcal{C}}\sum_{n\in\mathcal{N}}\sum_{t\in\mathcal{T}}\tau_t\left(u_{c,n,t,y}U_{c,n,t,y} + \nu_c D_{c,n,t,y}\right). \tag{S8}$$

$OPEX_y^{\mathrm{c}}$ is composed of a term attributed to the annual carbon emissions $E_y$ with the carbon price, $\mu$, and a term attributed to the annual carbon emission overshoot $E_y^{\mathrm{o}}$ with the carbon overshoot price $\mu^{\mathrm{o}}$:

$$OPEX_y^{\mathrm{c}} = E_y\mu + E_y^{\mathrm{o}}\mu^{\mathrm{o}}. \tag{S9}$$

### S2.3 Energy balance and carbon emission constraints

The sources and sinks of a carrier must be in equilibrium for all carriers in all nodes and at all time steps $t \in \mathcal{T}$. The source terms for carrier $c$ in node $n$ are:

- the output flow $\overline{G}_{c,i,n,t,y}$ of all conversion technologies $i \in \mathcal{I}$ if $c \in \overline{\mathcal{C}}_i$.
- the transported flow $F_{j,e,t,y}$ into the node from edge $e \in \underline{\mathcal{E}}_n$ minus the losses $F_{j,e,t,y}^{\mathrm{l}}$ for all transport technologies $j \in \mathcal{J}$ if $c = c_j^{\mathrm{r}}$.
- the discharge flow $\overline{H}_{k,n,t,y}$ for all storage technologies $k \in \mathcal{K}$ if $c = c_k^{\mathrm{r}}$.
- the imported flow $U_{c,n,t,y}$.

The sinks of carrier $c$ on node $n$ are:

- the exogenous demand $d_{c,n,t,y}$ minus the shed demand $D_{c,n,t,y}$.
- the input flow $\underline{G}_{c,i,n,t,y}$ of all conversion technologies $i \in \mathcal{I}$ if $c \in \underline{\mathcal{C}}_i$.
- the transported flow $F_{j,e',t,y}$ out of the node through edge $e' \in \overline{\mathcal{E}}_n$ for all transport technologies $j \in \mathcal{J}$ if $c = c_j^{\mathrm{r}}$.
- the charge flow $\underline{H}_{k,n,t,y}$ for all storage technologies $k \in \mathcal{K}$ if $c = c_k^{\mathrm{r}}$.

The energy balance for carrier $c \in \mathcal{C}$ is then calculated as:

$$0 = -\left(d_{c,n,t,y} - D_{c,n,t,y}\right) \tag{S10a}$$

$$+\sum_{i\in\mathcal{I}}\left(\overline{G}_{c,i,n,t,y} - \underline{G}_{c,i,n,t,y}\right) \tag{S10b}$$

$$+\sum_{j\in\mathcal{J}}\left(\sum_{e\in\underline{\mathcal{E}}_n}\left(F_{j,e,t,y} - F_{j,e,t,y}^{\mathrm{l}}\right) - \sum_{e'\in\overline{\mathcal{E}}_n}F_{j,e',t,y}\right) \tag{S10c}$$

$$+\sum_{k\in\mathcal{K}}\left(\overline{H}_{k,n,t,y}-\underline{H}_{k,n,t,y}\right) \tag{S10d}$$

$$+U_{c,n,t,y}. \tag{S10e}$$

Note that Eqs. (S10c) and (S10d) are zero if $c \neq c^{\mathrm{r}}_j$ and $c \neq c^{\mathrm{r}}_k$, respectively. In this study, we assume that no energy carrier can be exported across the borders of the energy system.

The total annual carbon emissions, $E_y$, account for the operational emissions of importing the carriers $c \in \mathcal{C}$ (carbon intensity $\epsilon_c$) and for operating the technologies $h \in \mathcal{H}$ (carbon intensity $\epsilon_h$):

$$E_y = \sum_{t\in\mathcal{T}} \tau_t \Bigg( \sum_{n\in\mathcal{N}} \Bigg( \sum_{c\in\mathcal{C}} \epsilon_c U_{c,n,t,y} + \sum_{i\in\mathcal{I}} \epsilon_i G^{\mathrm{r}}_{i,n,t,y} + \sum_{k\in\mathcal{K}} \epsilon_k \left(\overline{H}_{k,n,t,y} + \underline{H}_{k,n,t,y}\right) \Bigg) + \sum_{e\in\mathcal{E}} \sum_{j\in\mathcal{J}} \epsilon_j F_{j,e,t,y} \Bigg). \tag{S11}$$

The dual variable of Eq. (S11) is the optimal carbon abatement cost. The annual carbon emission limit $e_y$ constraints $E_y$ in all $y \in \mathcal{Y}$:

$$E_y \leq e_y. \tag{S12}$$

Note that $e_y$ can be infinite, in which case the constraint is skipped. The cumulative carbon emissions, $E^{\mathrm{c}}_y$, are attributed to the end of the current year. For the first planning period $y = y_0$, $E^{\mathrm{c}}_y$ is calculated as:

$$E^{\mathrm{c}}_y = E_y. \tag{S13}$$

In the subsequent periods $y > y_0$, $E^{\mathrm{c}}_y$ is calculated as:

$$E^{\mathrm{c}}_y = E^{\mathrm{c}}_{y-1} + \left(\Delta^{\mathrm{y}} - 1\right) E_{y-1} + E_y. \tag{S14}$$

$E^{\mathrm{c}}_y$ is constrained by the carbon emission budget, $e^{\mathrm{b}}$, at the end of the planning period $y$. Since we count the last planning period $Y$ as a single year (compare Eq. (S1)), the budget constraint for all $y \in \mathcal{Y} \setminus \{Y = \max(y)\}$ is formulated as:

$$E^{\mathrm{c}}_y + \left(\Delta^{\mathrm{y}} - 1\right) E_y - E^{\mathrm{o}}_y \leq e^{\mathrm{b}}. \tag{S15}$$

$E^{\mathrm{o}}_y$ is the cumulative carbon emission overshoot, which allows exceeding the carbon emission budget $e^{\mathrm{b}}$; however, $E^{\mathrm{o}}_y$ is heavily penalized (Eq. (S9)).

Eq. (S15) is calculated for $y = Y$ as:

$$E^{\mathrm{c}}_{Y} - E^{\mathrm{o}}_{y} \leq e^{\mathrm{b}}. \tag{S16}$$

Fig. S37 visualizes the carbon emission constraints Eqs. (S12) to (S16).

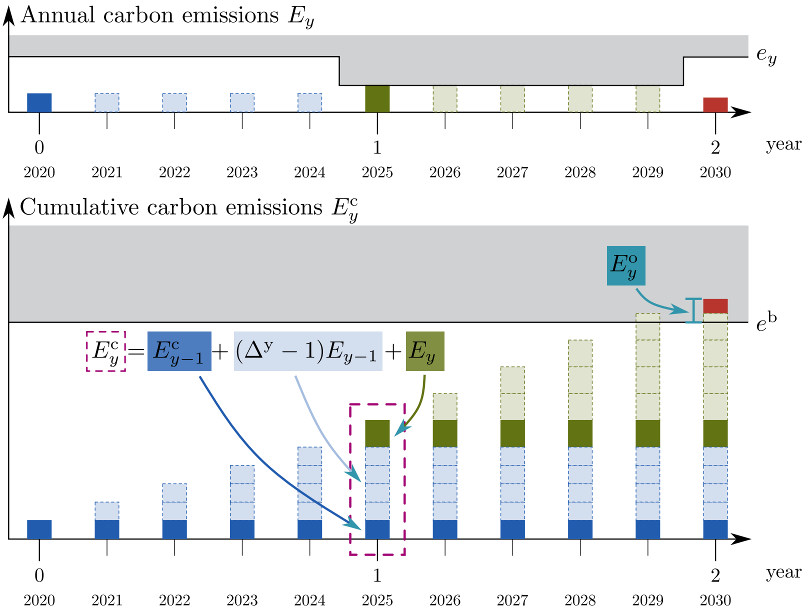


**Fig. S37** Schematic representation of cumulative carbon emission constraints (Eqs. (S12) to (S16)). Related to STAR Methods.

## S2.4 Operational constraints

The imported flow $U_{c,n,t,y}$ is constrained by the availability of carrier imports $a_{c,n,t,y}$ for all carriers $c \in \mathcal{C}$ in all nodes $n \in \mathcal{N}$ and time steps $t \in \mathcal{T}$:

$$0 \leq U_{c,n,t,y} \leq a_{c,n,t,y}. \tag{S17}$$

In this study, we prohibit the export of energy carriers across the energy system boundaries. The shed demand $D_{c,n,t,y}$ cannot exceed the demand $d_{c,n,t,y}$:

$$0 \leq D_{c,n,t,y} \leq d_{c,n,t,y}. \tag{S18}$$

The conversion factor $\eta_{i,c,t,y}$ is the ratio between the flow of carrier $c \in \mathcal{C}$ of conversion technology $i \in \mathcal{I}$ and the flow of the reference carrier $G^{\mathrm{r}}_{i,n,t,y}$. If the

input flow of carrier $c$ is considered ($c \in \underline{\mathcal{C}}_i$):

$$G^{\mathrm{r}}_{i,n,t,y}\eta_{i,c,t,y} = \underline{G}_{c,i,n,t,y}. \tag{S19}$$

If the output flow of carrier $c$ is considered ($c \in \overline{\mathcal{C}}_i$):

$$G^{\mathrm{r}}_{i,n,t,y}\eta_{i,c,t,y} = \overline{G}_{c,i,n,t,y}. \tag{S20}$$

The losses $F^{\mathrm{l}}_{j,e,t,y}$ through a transport technology $j \in \mathcal{J}$ on edge $e \in \mathcal{E}$ are the product of the loss coefficient $\rho_j$, the length of the edge $\lambda_{j,e}$ and the flow on the edge $F_{j,e,t,y}$:

$$F^{\mathrm{l}}_{j,e,t,y} = \rho_j \lambda_{j,e} F_{j,e,t,y}. \tag{S21}$$

The flow of the reference carrier $c^{\mathrm{r}}_h$ of all technologies $h \in \mathcal{H}$ is constrained by the maximum load $m_{h,p,t,y}$ and the capacity $S_{h,p,y}$. Examples for maximum load are renewable capacity factors (S3.4) or other reductions of the usable capacity (S3.7). For conversion technologies $i \in \mathcal{I}$, it follows:

$$0 \leq G^{\mathrm{r}}_{i,n,t,y} \leq m_{i,n,t,y} S_{i,n,y}. \tag{S22}$$

Analogously for transport technologies $j \in \mathcal{J}$:

$$0 \leq F^{\mathrm{r}}_{j,e,t,y} \leq m_{j,e,t,y} S_{j,e,y}. \tag{S23}$$

Since a storage technology does not charge ($\underline{H}_{k,n,t,y}$) and discharge ($\overline{H}_{k,n,t,y}$) at the same time, the sum of both flows is constrained by the maximum load:

$$0 \leq \underline{H}_{k,n,t,y} + \overline{H}_{k,n,t,y} \leq m_{k,n,t,y} S_{k,n,y}. \tag{S24}$$

Let $\sigma : \mathcal{T}^{\mathrm{k}} \rightarrow \mathcal{T}$ denote the unique mapping of a storage level time step $t^{\mathrm{k}}$ to a power-rated time step $t$, as obtained in the time series aggregation and representation (S2.6). The time-coupled equation for the storage level $L_{k,n,t^{\mathrm{k}},y}$ of storage technology $k$ at node $n$ is formulated for each storage level time step except the first $t^{\mathrm{k}} \in \mathcal{T}^{\mathrm{k}} \setminus \{0\}$ as:

$$\begin{aligned} L_{k,n,t^{\mathrm{k}},y} =& L_{k,n,t^{\mathrm{k}}-1,y} \left(1-\varphi_k\right)^{\tau^{\mathrm{k}}_{t^{\mathrm{k}}}} \\ &+ \left(\underline{\eta}_k \underline{H}_{k,n,\sigma(t^{\mathrm{k}}),y} - \frac{\overline{H}_{k,n,\sigma(t^{\mathrm{k}}),y}}{\overline{\eta}_k}\right) \sum_{\tilde{t}^{\mathrm{k}}=0}^{\tau^{\mathrm{k}}_{t^{\mathrm{k}}}-1} \left(1-\varphi_k\right)^{\tilde{t}^{\mathrm{k}}}, \end{aligned} \tag{S25}$$

with the self-discharge rate, $\varphi_k$, the charge and discharge efficiency, $\underline{\eta}_k$ and $\overline{\eta}_k$, respectively, and the duration of a storage level time step, $\tau^{\mathrm{k}}_{t^{\mathrm{k}}}$. Storage periodicity is enforced, by constraining the storage level in the first step of the

period ($t^{\mathrm{k}} = 0$) to be equal to the storage level in the last time step of the period ($t^{\mathrm{k}} = T^{\mathrm{k}}$), i.e., at the end of the year:

$$\begin{aligned} L_{k,n,0,y} =& L_{k,n,T^{\mathrm{k}},y} \left(1-\varphi_k\right)^{\tau^{\mathrm{k}}_{t^{\mathrm{k}}}} \\ &+ \left( \underline{\eta}_k \underline{H}_{k,n,\sigma(0),y} - \frac{\overline{H}_{k,n,\sigma(0),y}}{\overline{\eta}_k} \right) \sum_{\tilde{t}^{\mathrm{k}}=0}^{\tau^{\mathrm{k}}_{t^{\mathrm{k}}}-1} \left(1-\varphi_k\right)^{\tilde{t}^{\mathrm{k}}} . \end{aligned} \tag{S26}$$

We do not allow the carry-over of stored energy into the next year; hence, the storage levels are only coupled within the year. The non-negative $L_{k,n,t^{\mathrm{k}},y}$ is constrained by the energy-rated storage capacity $S^{\mathrm{e}}_{k,n,y}$:

$$0 \leq L_{k,n,t^{\mathrm{k}},y} \leq S^{\mathrm{e}}_{k,n,y}. \tag{S27}$$

$L_{k,n,t^{\mathrm{k}},y}$ is monotonous between $t^{\mathrm{k}}$ and $t^{\mathrm{k}}+1$. Hence, $L_{k,n,t^{\mathrm{k}},y}$ and $L_{k,n,t^{\mathrm{k}}+1,y}$ are the local extreme values and Eq. (S27) constrains the entire time interval between $t^{\mathrm{k}}$ and $t^{\mathrm{k}}+1$. We prove this in S2.7.

### S2.5 Investment constraints

The capacity, $S_{h,p,y}$, of a technology $h \in \mathcal{H}$ at a position $p \in \mathcal{P}$ in period $y$ is the sum of all previous capacity additions, $\Delta S_{h,p,y} \geq 0$, and existing capacity additions, $\Delta s^{\mathrm{ex}}_{h,p,y}$, that are still within their usable technical lifetime, $l_h$, (compare Eq. (S3)):

$$\begin{aligned} S_{h,p,y} =& \sum_{\tilde{y}=\max(y_0, y-\lceil l_h/\Delta^{\mathrm{y}}\rceil+1)}^{y} \Delta S_{h,p,\tilde{y}} \\ &+ \sum_{\hat{y}=\psi(\min(y_0-1, y-\lceil l_h/\Delta^{\mathrm{y}}\rceil+1))}^{\psi(y_0)} \Delta s^{\mathrm{ex}}_{h,p,\hat{y}}. \end{aligned} \tag{S28}$$

$S_{h,p,y}$ is constrained by the capacity limit, $s^{\max}_{h,p,y}$,:

$$0 \leq S_{h,p,y} \leq s^{\max}_{h,p,y}. \tag{S29}$$

In the case of constrained technology deployment, $\Delta S_{h,p,y}$ is constrained by the existing knowledge of how to install the technology, $K_{h,p,y}$, with the technology expansion rate, $\vartheta_h$. For node-based technologies, i.e., conversion and storage technologies, spillover effects from other nodes $\tilde{\mathcal{N}} = \mathcal{N} \setminus \{n\}$ can be utilized (knowledge spillover rate, $\omega$). To allow for an entry into a niche market, we add an unbounded market share, $\xi$, of the total capacity of all technologies with the same reference carrier in the previous year $y-1$:

$$\tilde{\mathcal{H}} = \left\{ \tilde{h} \in \mathcal{H} \mid c^{\mathrm{r}}_{\tilde{h}} = c^{\mathrm{r}}_{h} \right\} \tag{S30}$$

With the unbounded capacity addition, $\zeta_i$, it follows for the conversion technologies $i \in \mathcal{I}$:

$$\Delta S_{i,n,y} \leq \left((1+\vartheta_i)^{\Delta^{\mathrm{y}}} - 1\right) \left(K_{i,n,y} + \omega \sum_{\tilde{n}\in\tilde{\mathcal{N}}} K_{i,\tilde{n},y}\right) + \Delta^{\mathrm{y}} \left(\xi \sum_{\tilde{i}\in\tilde{\mathcal{I}}} S_{\tilde{i},n,y-1} + \zeta_i\right). \tag{S31}$$

If $y = y_0$, then $S_{\tilde{i},n,y-1}$ is the existing capacity in the current planning period. Analogously, for the storage technologies $k \in \mathcal{K}$:

$$\Delta S_{k,n,y} \leq \left((1+\vartheta_k)^{\Delta^{\mathrm{y}}} - 1\right) \left(K_{k,n,y} + \omega \sum_{\tilde{n}\in\tilde{\mathcal{N}}} K_{k,\tilde{n},y}\right) + \Delta^{\mathrm{y}} \left(\xi \sum_{\tilde{k}\in\tilde{\mathcal{K}}} S_{\tilde{k},n,y-1} + \zeta_k\right). \tag{S32}$$

We prohibit spillover effects for transport technologies $j \in \mathcal{J}$ from other edges as the planning of the transport infrastructure is heavily regulated by countries and the network operators:

$$\Delta S_{j,e,y} \leq \left((1+\vartheta_j)^{\Delta^{\mathrm{y}}} - 1\right) K_{j,e,y} + \Delta^{\mathrm{y}} \left(\xi \sum_{\tilde{j}\in\tilde{\mathcal{J}}} S_{\tilde{j},e,y-1} + \zeta_j\right). \tag{S33}$$

To avoid a feedback loop where country A profits from the knowledge in country B and simultaneously country B profits from the knowledge in country A, we constrain the capacity additions in all positions as follows:

$$\sum_{p\in\mathcal{P}} \Delta S_{h,p,y} \leq \sum_{p\in\mathcal{P}} \Bigg( \left((1+\vartheta_h)^{\Delta^{\mathrm{y}}} - 1\right) K_{h,p,y} \tag{S34}$$

$$+ \Delta^{\mathrm{y}} \left(\xi \sum_{\tilde{h}\in\tilde{\mathcal{H}}} S_{\tilde{h},p,y-1} + \zeta_h\right) \Bigg). \tag{S35}$$

$K_{h,p,y}$ is a function of the previous capacity additions, $\Delta S_{h,p,y}$ and $\Delta s^{\mathrm{ex}}_{h,p,y}$, as it represents the expertise and knowledge of the industry on how to install a certain amount of capacity. This knowledge is depreciated over time with the

knowledge depreciation rate $\delta$:

$$K_{h,p,y} = \sum_{\tilde{y}=y_0}^{y-1} (1-\delta)^{\Delta^{\mathrm{y}}(y-\tilde{y})} \Delta S_{h,p,\tilde{y}} + \sum_{\hat{y}=-\infty}^{\psi(y_0)} (1-\delta)^{(\Delta^{\mathrm{y}}(y-y_0)+(\psi(y_0)-\hat{y}))} \Delta s^{\mathrm{ex}}_{h,p,\hat{y}}. \tag{S36}$$

All investment constraints are formulated in the same way for the energy-rated storage capacities.

## S2.6 Time series aggregation and representation

To tackle computational complexity, we aggregate the time-dependent input data (the capacity factors of conversion technologies, the varying conversion factor of heat pumps, and electricity and heat demand) from hourly resolution to 100 representative time steps per year, using the time series aggregation package tsam 2.1.0 [9]. We utilize hierarchical clustering to ensure a deterministic sequence and the clusters are represented by their mean values. We avoid using k-means clustering as the current version of tsam does not support deterministic sequences for k-means [9], which would lead to different sequences among scenarios and model runs. The objective value and aggregated results are not significantly impacted by this choice.

The temporal representation of storage technologies is challenging because the storage constraints are time-coupled, thus the sequence of time steps must be preserved (Eq. (S25) [12]). To enable both the modeling of short- and medium-term storage, e.g., batteries, and long-term storage, e.g., natural gas and hydrogen storage, we develop a novel formulation, where the energy-rated storage variables are resolved on a different time sequence than the power-rated flow variables, presented in [8].

Fig. S38 schematically describes the time representation approach: Assume the representation of the exemplary full time index $\mathcal{T}^{\mathrm{full}} = [0, ..., 9]$ (blue line and time sequence) by four representative time steps $\mathcal{T} = [0, ..., 3]$. The time series aggregation yields a sequence of power-rated operational time steps (red line and time sequence). Any change in the aggregated time sequence for power-rated variables (red) yields an additional time step for the energy-rated storage variables (case II in Fig. S38). Two or more consecutive equal time steps (case I in Fig. S38) in the power-rated sequence (e.g., $[3, 3]$) are represented by the same storage time step (e.g., $[4, 4]$). The resulting sequence for energy-rated storage variables with the storage time steps $\mathcal{T}^{\mathrm{k}} = [0, ..., 6]$ is denoted in black in Fig. S38.

While this formulation enables both the short-term and long-term operation of storages, it increases the number of time steps $|\mathcal{T}^{\mathrm{k}}|$ and thus the number of variables. However, it reduces the number of time steps compared

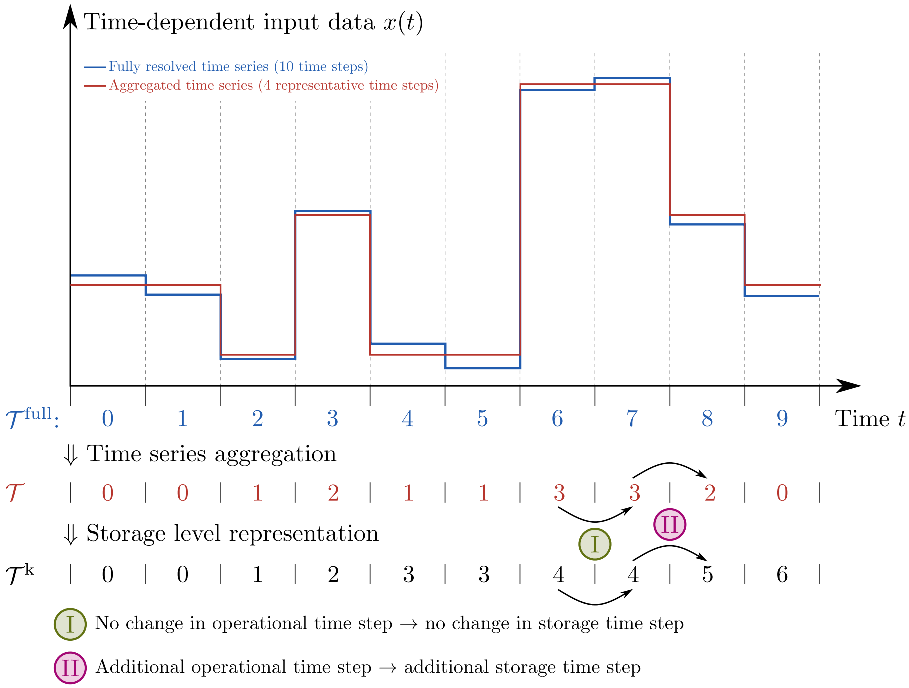


**Fig. S38** Time series representation from the fully resolved time series $\mathcal{T}^{\text{full}}$ to the aggregated operational time steps sequence $\mathcal{T}$ and the storage time steps sequence $\mathcal{T}^{\text{k}}$. Related to STAR Methods.

to the approach by Gabrielli et al. [12], which models the energy-rated storage variables on full time resolution (8760 time steps per year). Importantly, the reduction of a fully resolved storage level sequence to the representative storage time steps in Fig. S38 does not lead to any loss of information, since the storage behavior, i.e., the charging and discharging, is constant in the representative time steps.

## S2.7 Proof of storage level monotonicity

We prove that Eq. (S25) is monotonous on the entire time interval that is aggregated to a single storage time step $t^{\text{k}}$. Eq. (S27) provides upper and lower bounds for the storage level in each representative storage level time step $t^{\text{k}}$; however, it cannot constrain the hours between $t^{\text{k}}$ and $t^{\text{k}}+1$ [13]. By proving monotonicity, we show that the boundary values $L_{k,n,t^{\text{k}},y}$ and $L_{k,n,t^{\text{k}}+1,y}$ are the local extreme values and Eq. (S27) constrains the entire time interval between $t^{\text{k}}$ and $t^{\text{k}}+1$.

Consider Eq. (S25) for one storage time step $t^{\text{k}}$, during which $\underline{H}_{k,n,\sigma(t^{\text{k}}),y}$ and $\overline{H}_{k,n,\sigma(t^{\text{k}}),y}$ are constant. Neglecting all further indices without loss of generality, the storage level, $L(\hat{t})$, for the intermediate time steps $\hat{t} \in [1, \tau^{\text{k}}_{t^{\text{k}}}]$

follows as:

$$L(\hat{t}) = L_0 \kappa^{\hat{t}} + \Delta H \sum_{\tilde{t}=0}^{\hat{t}-1} \kappa^{\tilde{t}}, \tag{S37}$$

with $\kappa = 1 - \varphi$ and $\Delta H = \left(\eta \underline{H} - \frac{\overline{H}}{\eta}\right)$. $L_0$ is the storage level at the end of the previous storage time step $t^{\mathrm{k}} - 1$. Without self-discharge ($\varphi = 0 \Rightarrow \kappa = 1$), it follows:

$$L(\hat{t}) = L_0 + \Delta H \hat{t} \Rightarrow \frac{\mathrm{d}L(\hat{t})}{\mathrm{d}\hat{t}} = \Delta H. \tag{S38}$$

Since $\mathrm{d}L(\hat{t})/\mathrm{d}\hat{t}$ is independent of $\hat{t}$, Eq. (S37) is monotonous for $\varphi = 0$.

For $0 < \varphi < 1$, $\sum_{\tilde{t}=0}^{\hat{t}-1} \kappa^{\tilde{t}}$ is reformulated as the partial geometric series:

$$\sum_{\tilde{t}=0}^{\hat{t}-1} \kappa^{\tilde{t}} = \frac{1 - \kappa^{\hat{t}}}{1 - \kappa}. \tag{S39}$$

Eq. (S37) is reformulated to:

$$L(\hat{t}) = L_0 \kappa^{\hat{t}} + \Delta H \frac{1 - \kappa^{\hat{t}}}{1 - \kappa} = \frac{\Delta H}{1 - \kappa} + \left(L_0 - \frac{\Delta H}{1 - \kappa}\right) \kappa^{\hat{t}}. \tag{S40}$$

The derivative of Eq. (S40) follows as:

$$\frac{\mathrm{d}L(\hat{t})}{\mathrm{d}\hat{t}} = \underbrace{\left(L_0 - \frac{\Delta H}{1 - \kappa}\right) \ln(\kappa)}_{= \text{ constant } \forall \hat{t} \in [1, \tau^{\mathrm{k}}_{t^{\mathrm{k}}}]} \kappa^{\hat{t}}. \tag{S41}$$

With $\kappa^{\hat{t}} > 0$, it follows that Eq. (S37) is monotonous for $0 < \varphi < 1$. Thus, the storage level constraints Eq. (S27) apply to the entire time interval, even though not every hour is modeled.

# S3 Input data of case study

## S3.1 Scope of case study

We model the European energy system as a network representation, consisting of 28 nodes (countries) and edges between them (aggregated power lines, natural gas pipelines, and carbon pipelines).

1. Austria (AT)
2. Belgium (BE)
3. Bulgaria (BG)
4. Switzerland (CH)
5. Czech Republic (CZ)
6. Germany (DE)
7. Denmark (DK)
8. Estonia (EE)
9. Greece (EL)
10. Spain (ES)
11. Finland (FI)
12. France (FR)
13. Croatia (HR)
14. Hungary (HU)
15. Ireland (IE)
16. Italy (IT)
17. Lithuania (LT)
18. Luxembourg (LU)
19. Latvia (LV)
20. The Netherlands (NL)
21. Norway (NO)
22. Poland (PL)
23. Portugal (PT)
24. Romania (RO)
25. Sweden (SE)
26. Slovenia (SI)
27. Slovakia (SK)
28. United Kingdom (UK)

We choose a spatial resolution at the national level which for most countries corresponds to the control and bidding zones for transmission system operators in the electricity and gas sector. A finer spatial resolution is avoided due to computational limitations. We set the trade-off between temporal and spatial resolution in favor of the temporal resolution because of the importance of capturing the temporal variability of electricity and heat supply, especially in light of higher renewable integration and accurate modeling of energy storage. We acknowledge that a higher spatial resolution improves the representation of renewable generators and transport constraints [14].

We extract all hourly resolved input data for the year 2019, as it is the last year before the Covid19 pandemic.

Tables S3 and S4 summarize the conversion, transport, and storage technologies included in this study. We assume linear time-independent and location-independent conversion efficiencies, except for heat pumps. The capacities are mostly rated by the output carrier, i.e., capacities of electricity generation technologies are rated by their generated electric power, and capacities of heat generation technologies by their generated thermal power.

The time- and location-dependent conversion factors of heat pumps (for both onsite and district heat generation) are extracted from the When2Heat project for 2019 [22]. To account for the difference in conversion factor between the two types, and between idealized calculations and real-world measurements, we scale the time series with their average conversion factors from [16, 18].

**Table S3** Conversion technologies in optimization model with respective output and input carrier(s), conversion efficiency, and lifetime. The capacities of technologies are rated by their main output carrier, except for carbon storage. CCGT: combined-cycle gas turbine, DH: district heating. Related to STAR Methods.

| **Conversion technology** | **Output carrier** | **Second output carrier** | **Input carrier** | **Efficiency** | **Lifetime** |
|---|---|---|---|---|---|
| Natural gas turbine (CCGT) | Electricity | | Natural gas | 0.532 [15] | 30 [16–20] |
| CCGT with CCS | Electricity | Carbon | Natural gas | 0.488 [18, 19] | 31 [17–20] |
| Hard coal plant | Electricity | | Hard coal | 0.391 [15] | 46 [21] |
| Hard coal plant with CCS | Electricity | Carbon | Hard coal | 0.407 [18, 19] | 40 [17–19] |
| Lignite coal plant | Electricity | | Lignite | 0.379 [15] | 46 [21] |
| Nuclear power plant | Electricity | | Uranium | 0.339 [15] | 55 [17–20] |
| Oil power plant | Electricity | | Oil | 0.386 [15] | 25 [16, 17, 19] |
| Waste power plant | Electricity | | Waste | 0.284 [15] | 27 [16, 18–20] |
| Biomass power plant | Electricity | | Biomass | 0.378 [15] | 30 [16–20] |
| Biomass power plant with CCS | Electricity | Carbon | Biomass | 0.314 [18, 19] | 40 [17–19] |
| Onshore wind | Electricity | | | | 25 [16–20] |
| Offshore wind | Electricity | | | | 25 [16–20] |
| Solar PV | Electricity | | | | 30 [16–20] |
| Run-of-river hydropower | Electricity | | | | 50 [17–20] |
| Reservoir hydropower | Electricity | | | | 60 [17–20] |
| Natural gas boiler | Heat | | Natural gas | 0.995 [16] | 21 [16, 20] |
| Oil boiler | Heat | | Oil | 0.920 [16] | 20 [16, 20] |
| Biomass boiler | Heat | | Biomass | 0.835 [16] | 20 [16, 20] |
| Heat pump | Heat | | Electricity | variable [16, 22] | 19 [16, 20] |
| Electric boiler | Heat | | Electricity | 1.000 [16] | 30 [16, 20] |
| Natural gas boiler DH | District heat | | Natural gas | 0.968 [16, 18] | 25 [16, 18, 20] |
| Hard coal boiler DH | District heat | | Hard coal | 0.817 [18] | 30 [18, 20] |
| Oil boiler DH | District heat | | Oil | 0.956 [16, 18] | 25 [16, 18, 20] |
| Waste boiler DH | District heat | | Waste | 0.936 [16, 18] | 30 [16, 18] |
| Biomass boiler DH | District heat | | Biomass | 0.982 [16, 18] | 25 [16, 18, 20] |
| Heat pump DH | District heat | | Electricity | variable [16, 18, 22] | 25 [16, 18, 20] |
| Electric boiler DH | District heat | | Electricity | 0.985 [16, 18] | 20 [16, 18, 20] |
| District heating grid | Heat | | District heat | 0.860 [23] | 40 [23] |
| LNG terminal | Natural gas | | LNG | 1 | 30 [24] |
| Carbon storage | | | Carbon | 0.999 [25] | 40 [26] |
| Industrial gas consumer | Industrial gas | | Natural gas | 1 | 100 |

**Table S4** Transport and storage technologies in optimization model with reference carrier and lifetime. Relative loss of transport technologies is per distance and transported energy. For the storage technologies, we assume equal discharging and charging efficiencies and no self-discharge [27]. All parameter values without a reference are assumed. Related to STAR Methods.

| **Transport technology** | **Reference carrier** | **Lifetime** | **Relative loss [1/km]** |
|---|---|---|---|
| Power line | Electricity | 60 [28] | 0.00005 [28] |
| Natural gas pipeline | Natural gas | 60 [23] | 0.00005 |
| Carbon pipeline | Carbon | 50 [29] | 0.00005 |

| **Storage technology** | **Reference carrier** | **Lifetime** | **Round-trip efficiency** |
|---|---|---|---|
| Pumped hydro storage | Electricity | 55 [30] | 0.780 [30] |
| Battery | Electricity | 13 [30] | 0.860 [30] |
| Hydrogen storage | Electricity | 100 [31] | 0.400 [30] |
| Natural gas storage | Natural gas | 100 | 0.995 |

Modern conventional power plants show a higher conversion efficiency than those installed in the past. However, the existing fleet in Europe is dominated by older units, mainly from the 1970s to 1990s in the case of hard coal power plants [21]. To avoid overestimating the conversion efficiency of the existing fleet by assuming the efficiency of new-generation power plants, we calculate the observed, average conversion factor of conventional electricity generation in Europe from the Eurostat Complete Energy Balances [15].

Hard coal and lignite power plants often show longer technical lifetimes than the assumed economic lifetime, which leads to the premature decommissioning of existing and still operating coal power plants in the model. Hence, we assume a lifetime of 46 years, which is the average lifetime of coal power plants [21].

Note that we do not model the electricity, gas, and carbon network in detail but use a flow network representation with aggregated cross-border capacities. We simplify the complex structure of the European power, natural gas, and carbon network by assuming that the connections of transport technologies span between the centroids of the connected country nodes. Furthermore, we do not model the physical flows through power lines and pipelines in detail, but assume a simplified, linear commodity transport between nodes.

## S3.2 Existing capacity - electricity sector

The existing capacities of electricity generation technologies are obtained from the Bloomberg New Energy Finance (BNEF) platform [32]. Many other, open-source datasets, such as the ENTSO-E Transparency Platform [33], cannot be used as they do not report the construction years, which are essential to determine the decommissioning date at the end of the technical lifetime and the technology expansion limits. We find that the BNEF dataset shows better data quality than the Open Power System Data [34, 35] or the IRENA Renewable Capacity Statistics [36], especially in terms of spatial completeness and accuracy in construction years. The BNEF project database only reports larger-scale utility projects, which do not encompass all residential and commercial solar PV installations. Thus for solar PV, we use the BNEF solar PV database with all three solar PV types (residential, commercial, and utility). The data are validated with the total existing capacities from the ENTSO-E Transparency Platform [33]. Because of better data quality, we obtain the existing capacities for run-of-river hydropower and reservoir hydropower from the JRC Hydro-power database [37]. We report the country-aggregated data of all technologies in the Zenodo data repository (https://zenodo.org/records/11074289).

We assume that pumped hydro storage is the only electricity storage technology that shows substantial existing capacity. The existing capacity of pumped hydro storage is obtained from the JRC Hydro-power database [37]. It is observed that the energy-rated capacity is often significantly higher than reported in other databases [34, 36], especially for generator units that are used both as pumped hydro storages and as reservoir hydropower plants. Hence, we limit the energy-rated capacity by the maximum dischargeable energy. To this end, we multiply the power-rated discharge capacity with the maximum discharge time of pumped hydro storages (16h [38]), divided by the discharge efficiency [30].

The existing capacity of the aggregated power lines is extracted as the net transfer capacity (NTC) from the ENTSO-E Transparency Platform [33] for 2022. For those edges between countries for which no NTC is reported, we set the existing capacity to the maximum cross-border flow in 2022 from [33].

The modeled gas supply infrastructure is described in [8].

## S3.3 Capacity limit - electricity sector

The country-specific capacity limits for onshore wind, offshore wind, and solar PV are extracted from the ENSPRESO database [39]. The capacity of hydropower plants is limited by the existing capacity, i.e., we follow the common assumption that existing capacities of hydropower plants can be rebuilt once decommissioned but not expanded [40]. Some researchers believe that the economically feasible potential of hydropower in Europe is already exhausted [41, 42]. In particular, the long permitting process and local opposition to hydropower projects suggest little additional capacity [43]. In Europe, the IEA projects the untapped economic potential to be 29% [6]. To test the robustness of the findings against this assumption, we perform a sensitivity analysis in S1.7.

The investment in nuclear power is peculiar due to its high lead times, around 10 years, which increases the investment risk and decreases the probability of deployment during the energy transition [44]. However, we neglect lead times in this study since the myopic optimizer would not invest in technologies with lead times longer than the foresight horizon. Thus, we tackle the installation characteristics of nuclear by limiting the capacity to the existing capacity today and assume that European countries will only i) phase out nuclear power, ii) extend the lifetime of existing nuclear power plants, or iii) build small capacities of small modular reactors, as projected by the Nuclear Energy Agency [45].

The expansion of the power line network is limited by the candidate units of the 10-year network development plan (TYNDP) of the ENTSO-E and ENTSOG [46]. The candidate units are aggregated to the corresponding cross-border connection and added to the existing net-transfer capacities.

### S3.4 Capacity factors - electricity sector

The hourly capacity factors for solar PV, and onshore and offshore wind are obtained from Renewables.ninja, resolved on NUTS0 level [47, 48]. Nuclear power plants show a strong seasonality in their capacity factors with significant unavailability due to maintenance and refueling during summer. We calculate the hourly capacity factor for the European nuclear capacity stock as the average capacity factor for each hour of the historical values from 2018 until 2021 [33]. We consider the representation of the nuclear capacity factor time series subordinate to the accurate representation of the demand time series of electricity and heat and capacity factor time series of renewable generators. Hence, we exclude the nuclear capacity factors from the time series aggregation to avoid influencing the accurate clustering of the demand time series and renewable capacity factors. After conducting the time series aggregation with mean representation [9], we analogously aggregate the nuclear capacity factor by representing each time step as the mean value of each corresponding cluster.

## S3.5 Existing capacity - heating sector

It is challenging to retrieve data on the existing capacities of heating technologies. The Heat Roadmap Europe 4 (HRE) project [49] provides values for the existing heating capacities, however, this information is limited to 14 European countries and the data is only available for 2015 and a broad aggregation of technology types. Other databases such as [50] include more countries, but do not report construction years or provide more recent data.

Hence, we model the heating capacities based on the generated heat from 1990 until 2020, as reported in the complete energy balance by Eurostat [15]. We assume that the capacity share, $r_{i,n}$, of a heating technology $i \in \mathcal{I}$ in country $n \in \mathcal{N}$ is proportional to its share in historic heat generation, and the sum of all capacities can supply the peak heat demand in a country. The yearly difference between the calculated capacities yields the capacity additions of each year. We account for previous capacity additions by assuming that the existing capacities in 1990 (the first year of reporting in Eurostat) were accumulated by constant previous capacity additions. The yearly difference in total existing capacity represents net-capacity additions but does not account for the compensation of decommissioned units after their technical lifetime. Therefore, we calculate the decommissioned capacities in each year and add it to the capacity additions.

We distinguish between onsite (individual) and offsite (district heating) technologies and extract the heat generated onsite from the final consumption energy balance in other sectors (residential and commercial) and the heat generated offsite from the gross heat production energy balance [15]. We allocate peat, peat products, and nuclear heat to hard coal boilers, and manufactured gases to gas boilers. Nuclear heat is different from coal-based heat; however, nuclear heat only accounts for 0.17% of heat generation (exclusively offsite). Biomass boilers entail both solid and gaseous biomass. The final consumption energy balance is given in quantities of the input carriers, while the gross heat production is given in quantities of generated heat. Thus, we multiply the final consumption energy balance with the conversion efficiencies from Table S3 to obtain the generated heat in the residential and commercial sectors. We scale the gross heat production energy balance to match the derived heat consumed in the residential and commercial sectors.

Electricity is the only energy carrier that is widely consumed for other services than heating and cooling in the residential and commercial sectors. Thus, we subtract the share of residential electricity consumption that is not used for heating and cooling, obtained from Eurostat's disaggregated final energy consumption in households energy balance (NRG_D_HHQ) [51]. Two heat generation technologies consume electricity, namely, heat pumps and electric boilers. The ambient heat (heat pumps) energy carrier in the complete energy balances [15] is not consistent with other databases [49, 52]. Thus, we utilize the IDEES 2015 database [52] to obtain the ratio between electric boilers (conventional electric heating) and heat pumps (advanced electric heating) for 2015. We assume that Norway (NO) has the same ratio between electric

boilers and heat pumps as Sweden (SE). Furthermore, we assume that Switzerland (CH) has the same technology share for all technologies as Austria (AT). We acknowledge that the shares of heat pumps and electric boilers may have shifted since 2015; however, no better data are available. In general, the presented approach yields total capacities in line with those available in the HRE4 project [49]. We report the country-aggregated data of all technologies in the data repository.

### S3.6 Capacity limit - heating sector

The economic and technical feasibility of district heating (DH) depends on the density of the designated area of deployment [53]. Hence, there are many rural areas in Europe where the heat supply through a DH grid is highly unlikely. We calculate the capacity limits of DH by utilizing the feasible share of DH obtained from the HRE4 project [49]. To extend the findings of the 14 countries in HRE4 to all 28 countries in this study, we correlate the DH potential with the share of the population living in urban (u), suburban (s), and rural (r) areas [54, 55]:

$$p_n^{\mathrm{DH}} = f^{\mathrm{u}} s_n^{\mathrm{u}} + f^{\mathrm{s}} s_n^{\mathrm{s}} + f^{\mathrm{r}} s_n^{\mathrm{r}}, \tag{S42}$$

with the DH potential $p_n^{\mathrm{DH}}$ for all countries $n \in \mathcal{N}$, the three population shares $s_n^{\mathrm{u}}$, $s_n^{\mathrm{s}}$, and $s_n^{\mathrm{r}}$ for all countries $n \in \mathcal{N}$, and the three area correlation factors $f^{\mathrm{u}}$, $f^{\mathrm{s}}$, and $f^{\mathrm{r}}$. Table S5 shows the population shares and the DH potential from HRE4. A linear regression yields area correlation factors of $f^{\mathrm{u}} = 0.937$, $f^{\mathrm{s}} = 0.489$, and $f^{\mathrm{r}} = 0.282$. Thus, 93.7% of urban areas could be supplied by district heating, whereas only 28.2% of rural areas could be connected to a DH grid. The projected DH potentials from Table S5 (see Fig. S39) are then multiplied by the countries' peak heat demand to obtain the capacity limit of district heating. The capacity of other heat generation technologies is not constrained.

**Table S5** District heating (DH) potential in Europe. Potentials for 14 countries obtained from HRE4 (first block of countries [49]) and correlated with the urban, suburban, and rural share of the population [54, 55]. The DH potential is extended for the remaining 14 countries of this case study (second block of countries). The area correlation factors for urban, suburban, and rural areas are $f^{\mathrm{u}} = 0.937$, $f^{\mathrm{s}} = 0.489$, and $f^{\mathrm{r}} = 0.282$, respectively. Related to STAR Methods.

| Country | Share urban [54, 55] | Share suburban [54, 55] | Share rural [54, 55] | DH potential HRE4 [49] | DH potential correlation | DH capacity limit [GW] |
|---|---|---|---|---|---|---|
| AT | 0.32 | 0.28 | 0.40 | 0.55 | 0.55 | 14.13 |
| BE | 0.54 | 0.38 | 0.08 | 0.55 | 0.71 | 26.98 |
| CZ | 0.25 | 0.53 | 0.21 | 0.69 | 0.56 | 17.00 |
| DE | 0.44 | 0.41 | 0.16 | 0.79 | 0.65 | 157.49 |
| ES | 0.64 | 0.33 | 0.03 | 0.81 | 0.77 | 29.77 |
| FI | 0.31 | 0.30 | 0.39 | 0.53 | 0.55 | 10.44 |
| FR | 0.35 | 0.37 | 0.28 | 0.60 | 0.59 | 92.32 |
| HU | 0.18 | 0.64 | 0.18 | 0.43 | 0.53 | 15.00 |
| IT | 0.48 | 0.42 | 0.10 | 0.82 | 0.68 | 105.93 |
| NL | 0.74 | 0.25 | 0.01 | 0.77 | 0.82 | 37.30 |
| PL | 0.25 | 0.39 | 0.36 | 0.45 | 0.53 | 43.21 |
| RO | 0.12 | 0.34 | 0.54 | 0.44 | 0.43 | 5.69 |
| SE | 0.40 | 0.51 | 0.09 | 0.59 | 0.65 | 14.88 |
| UK | 0.39 | 0.44 | 0.17 | 0.73 | 0.63 | 97.70 |
| BG | 0.19 | 0.68 | 0.13 | - | 0.55 | 5.66 |
| CH | 0.46 | 0.51 | 0.03 | - | 0.69 | 23.96 |
| DK | 0.23 | 0.49 | 0.28 | - | 0.53 | 11.61 |
| EE | 0.56 | 0.00 | 0.44 | - | 0.65 | 2.67 |
| EL | 0.47 | 0.23 | 0.30 | - | 0.64 | 16.18 |
| HR | 0.20 | 0.37 | 0.43 | - | 0.49 | 4.75 |
| IE | 0.29 | 0.15 | 0.57 | - | 0.50 | 5.36 |
| LT | 0.29 | 0.63 | 0.08 | - | 0.60 | 3.60 |
| LU | 0.00 | 1.00 | 0.00 | - | 0.49 | 1.31 |
| LV | 0.32 | 0.46 | 0.22 | - | 0.59 | 2.91 |
| NO | 0.13 | 0.66 | 0.21 | - | 0.50 | 9.92 |
| PT | 0.47 | 0.22 | 0.31 | - | 0.64 | 1.93 |
| SI | 0.00 | 0.42 | 0.58 | - | 0.37 | 1.68 |
| SK | 0.13 | 0.50 | 0.37 | - | 0.47 | 3.99 |

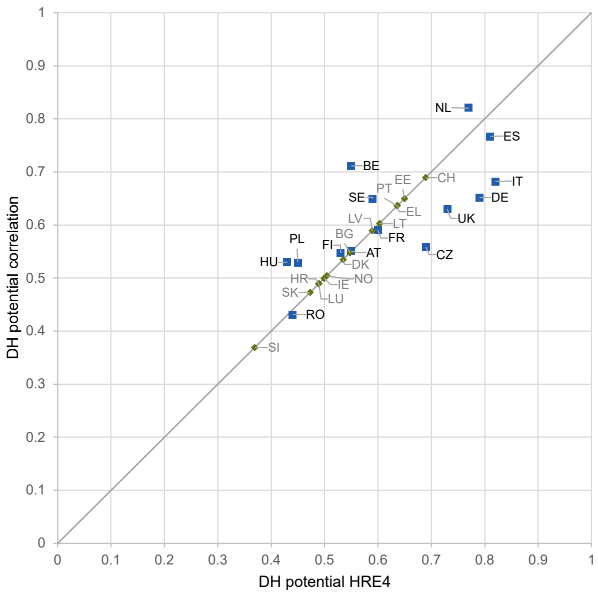


**Fig. S39** District heating (DH) potential, reported by HRE4 [49] on the x-axis and calculated in this study on the y-axis (Table S5). Countries for which the values are reported by HRE4 are in blue; missing countries are in green (data points for missing countries are located on the identity line because no values from HRE4 exist). Related to STAR Methods.

## S3.7 Supply substitution - heating sector

In contrast to electricity, the heat supply cannot be easily substituted by another fuel even if idle capacity is available. In fact, most heat is generated onsite with only one energy carrier (monovalent operation). Furthermore, the heat cannot be shared with other buildings but must be consumed onsite where it is produced. However, we cannot distinguish between individual buildings in this nationally aggregated case study. Thus, we apply a capacity factor to all heat generation technologies so that the usable capacity of the heat generation technologies is seemingly reduced in times of lower heat demand. By doing so, we can prevent the substitution of heat supply between buildings without modeling each building individually.

As an example, on a late summer day with a heat demand of a quarter of the peak demand, we reduce the usable capacity of the onsite heat generation technologies to 25%. Hence, even though not the entire nominal capacity is utilized at that moment, the capacity cannot be used to substitute the heat generation in another building but can only supply the heat demand of the building in which it is installed. We neglect that some buildings are supplied by two onsite heat sources, such as a gas boiler and a heat pump.

In district heating (DH) grids, the use of multiple fuels is more common than in onsite heat generation. However, especially small DH grids in suburban and rural areas are often operated by a single fuel source. Due to the lack of relevant data, we assume that 100% of the heat supply can be substituted in urban DH grids, 50% in suburban DH grids, and 0% in rural DH grids. We calculate the share of substitutable district heat by utilizing the urban, suburban, and rural share of the supplied district heat for each country, multiplied with the share of substitutable district heat for each location type (urban, suburban, rural; Table S5).

Fig. S40 shows a schematic representation of the substitution problem for two buildings with onsite heat generation technologies and two buildings connected to a DH grid each, one supplied by one technology and the other one supplied by multiple technologies. The DH substations, i.e., the connection points to the buildings, are equal to onsite heating technologies in that they cannot substitute heat supply.

Since the time series of these capacity factors are not a description of a physical property but a modeling tool to prohibit the supply substitution, we consider them subordinate to actual physical time series, such as demands or capacity factors of renewable generators. Therefore, we exclude the capacity factors of heating technologies from the time series aggregation to avoid influencing the accurate representation of the demand time series of electricity and heat, the conversion factor of heat pumps, and the capacity factors of renewable generators. After conducting the time series aggregation with a hierarchical clustering algorithm and mean representation, we analogously aggregate the capacity factors of the heating technologies by representing each time step as

the mean value of each corresponding cluster. We show in S1.10 that the supply substitution of onsite heat technologies has a considerable impact on the results; however, the share of substitutable DH capacities does not.

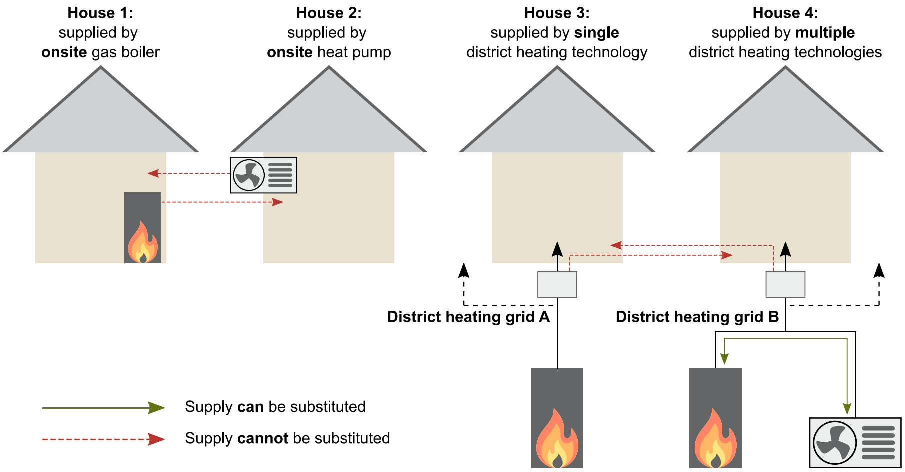


**Fig. S40** Heat supply substitution problem. Onsite heat generation technologies and district heating substations (Houses 1-4) cannot supply heat to other buildings. Only a district heating grid with multiple heat generation technologies can substitute district heat from other technologies in the same district heat grid. Houses 3 and 4 are connected to different district heating grids A and B. Related to STAR Methods.

## S3.8 Energy carriers - import availability, prices, carbon intensity

The calculation of hourly electricity, heat, and industrial gas demand is described in [8]. This study only investigates the electricity and heating sector and does not utilize any projection of how the electricity demand might evolve as a result of higher electrification in other sectors, e.g., transportation or industry, as this also influences the allocation of carbon emissions to the various sectors, and hence the remaining carbon budget.

Following the Russian invasion of Ukraine in February 2022, Russian natural gas imports were almost reduced to zero [8]. It is not likely that Russian gas imports will return, and thus we assume that Russian gas will remain unavailable for the entire time horizon. The availability of non-Russian natural gas and liquefied natural gas (LNG) imports are detailed in [8]. Biomass is projected to play an increasingly important role in many hard-to-abate sectors [56], which constrains the availability of biomass in the electricity and heating sector. Thus, we assume that the biomass availability will remain equal to the consumption in 2021, as reported by Eurostat [15]. The imports of all other fuels are unconstrained.

Table S6 lists the carbon intensities of the fuels [57]. We neglect upstream emissions, as they are not part of the national reporting to the UNFCCC [57]. The carbon intensity of hard coal is calculated as the arithmetic mean of anthracite, coking coal, and other bituminous coal, weighted by their respective transformation input in Europe in 2021 [15]. The carbon intensity of lignite is calculated as the arithmetic mean of lignite and sub-bituminous coal, weighted by their respective transformation input in Europe in 2021 [15]. Table S7 lists the assumed fuel prices of imported energy carriers.

**Table S6** Carbon intensity of fuel input [57]. Related to STAR Methods.

| Carrier | Carbon intensity [$tCO_2$/MWh] |
|---|---|
| Hard coal | 0.341 |
| Lignite | 0.364 |
| Oil | 0.267 |
| Natural gas | 0.202 |
| LNG | 0.202 |
| Uranium | 0.000 |
| Waste | 0.330 |
| Biomass[1] | 0.403 |

[1] Imported biomass is assumed to be carbon neutral, but the carbon content is considered for biomass plants with carbon capture and storage (CCS).

**Table S7** Fuel prices of energy carriers that can be imported in 2021 EUR/MWh. We assume country-independent prices. Waste is treated as an unavoidable byproduct and thus does not have a price. Annual values are available for fuel prices obtained from [58], but only selection is shown here. Values from 2016 to 2021 are used for hindcasting. Related to STAR Methods.

| Carrier | 2016 | 2022 | 2025 | 2030 | 2035 | 2040 | 2045 | 2050 | Source |
|---|---|---|---|---|---|---|---|---|---|
| Hard coal | 10.69 | 31.17 | 10.63 | 6.75 | 6.30 | 6.08 | 6.08 | 6.07 | [58] |
| Oil | 25.06 | 36.21 | 29.33 | 29.09 | 29.09 | 29.09 | 29.09 | 29.09 | [58] |
| Natural gas | 13.65 | 32.43 | 14.18 | 16.07 | 16.64 | 17.11 | 17.62 | 18.15 | [58] |
| LNG | 13.65 | 32.43 | 14.18 | 16.07 | 16.64 | 17.11 | 17.62 | 18.15 | [58] |
| Uranium | 1.69 | 1.69 | 1.69 | 1.69 | 1.69 | 1.69 | 1.69 | 1.69 | [19] |
| Lignite | 3.96 | 3.96 | 3.96 | 3.96 | 3.96 | 3.96 | 3.96 | 3.96 | [19] |
| Biomass | 26.65 | 27.58 | 28.05 | 28.83 | 30.14 | 31.45 | 32.75 | 34.06 | [59] |

### S3.9 Carbon supply chain

We model the carbon supply chain to consist of three elements (Tables S3 and S4):

1. Electricity generation plants retrofitted with carbon capture technologies, namely hard coal power plants with carbon capture, natural gas turbines with carbon capture, and biomass power plants with carbon capture (referred to as bioenergy with carbon capture and storage, or BECCS).
2. Carbon pipelines to model carbon flows between the centroids of the country nodes.
3. Carbon storage, modeled as a conversion technology that consumes sequestered carbon with a carbon intensity of -1 $tCO_2/tCO_2$.

We assume no existing capacity and no retrofitting of existing natural gas pipelines or storage to transport and store carbon. The capacity limit for the carbon storage location is extracted from [60] and reported in Table S8. The reduced capacity limit only includes projects that are specifically designated or might be designated for carbon capture and storage (CCS) in the electricity sector, such as BECCS. In S1.8, we investigate the impact of the carbon storage capacity limit.

**Table S8** Capacity limit of carbon storage in $MtCO_2$ per year [Mtpa] [60]. Reduced capacity limit only includes projects that might be designated for CCS in the electricity sector. Related to STAR Methods.

| Country | Capacity limit [Mtpa] | Reduced capacity limit [Mtpa] |
|---|---|---|
| BE | 17.8 | 0.0 |
| DE | 3.9 | 0.0 |
| DK | 27.0 | 3.0 |
| EL | 6.0 | 6.0 |
| FI | 0.4 | 0.0 |
| FR | 12.4 | 0.0 |
| HR | 0.2 | 0.0 |
| IE | 2.5 | 2.5 |
| IT | 4.0 | 4.0 |
| NL | 18.1 | 12.5 |
| NO | 29.3 | 25.0 |
| SE | 3.1 | 0.0 |
| UK | 20.8 | 7.4 |

Carbon pipelines and storage are the only technologies with an unbounded capacity addition $\zeta_h = 10$ $tCO_2$/hour (0.0876 Mtpa) because there is no existing market of carbon technologies yet (see Eqs. (S31) and (S33)). The unbounded capacity addition is chosen to be in the range of initial carbon storage projects in Europe [61].

### S3.10 Cost parameters

The assumed cost parameters of the electricity and heat generation technologies are obtained from [16–20, 62], are listed in Excel Table S9, and presented in Figs. S41 to S43. The cost parameters of the storage technologies (except natural gas storages) are obtained from [30, 31] and listed in Excel Table S9. Because of the strong differences in the assumptions across sources, we select the average value across sources (first average for each source and then average across sources) and investigate the sensitivity of the solution to the cost assumption in S1.1. Due to the lack of data for individual hard coal boilers, we assume the average cost parameters of individual biomass boilers due to the technologies' similar structure and operation. All cost parameters are converted to 2021 Euro. The cost parameters of all other technologies are listed in Excel Table S9. All missing parameters are assumed to be zero.

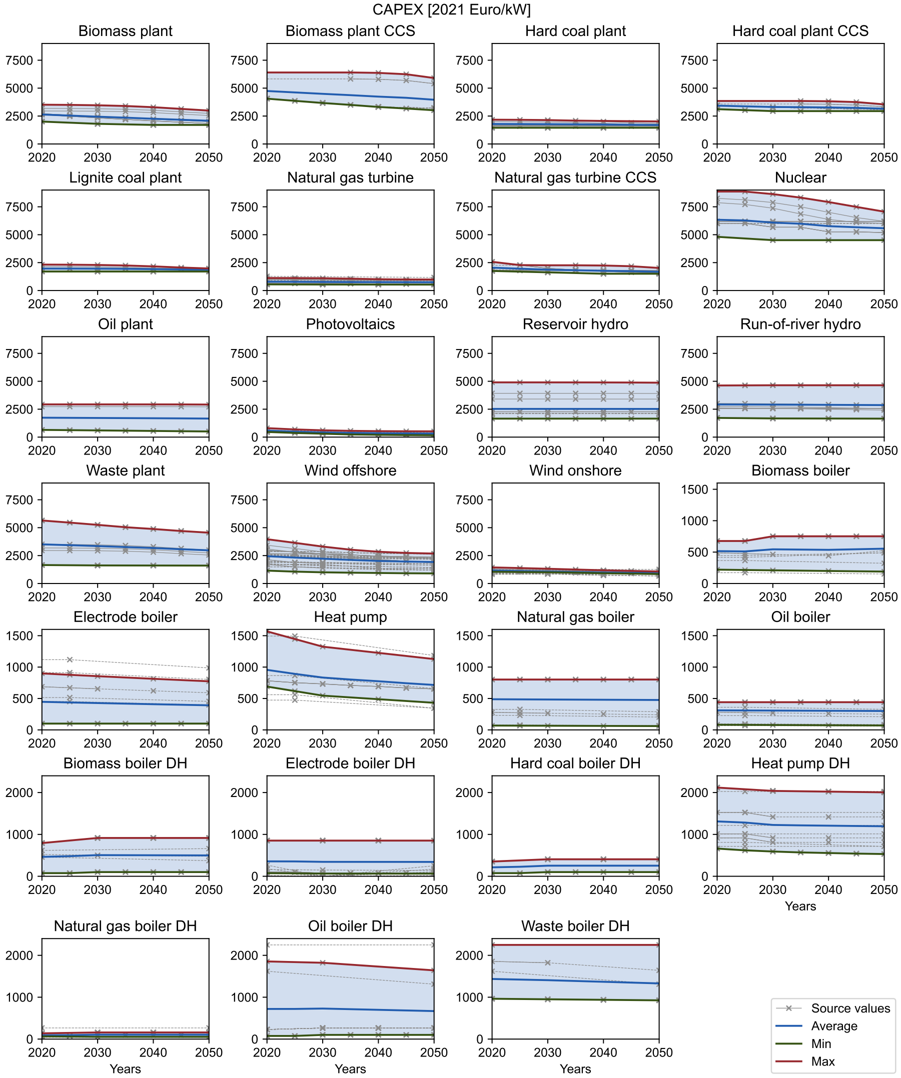


**Fig. S41** Evolution of capital expenditure (CAPEX) for conversion technologies from several sources from 2020 to 2050 in 2021 EUR/kW (see Table S9). The average value is in blue, minimum in green, and maximum in red. The reported values are averaged for each source (grey lines) and averaged across sources. The dashed lines are the interpolation between values that are more than 5 years apart. DH: district heating. Related to STAR Methods.

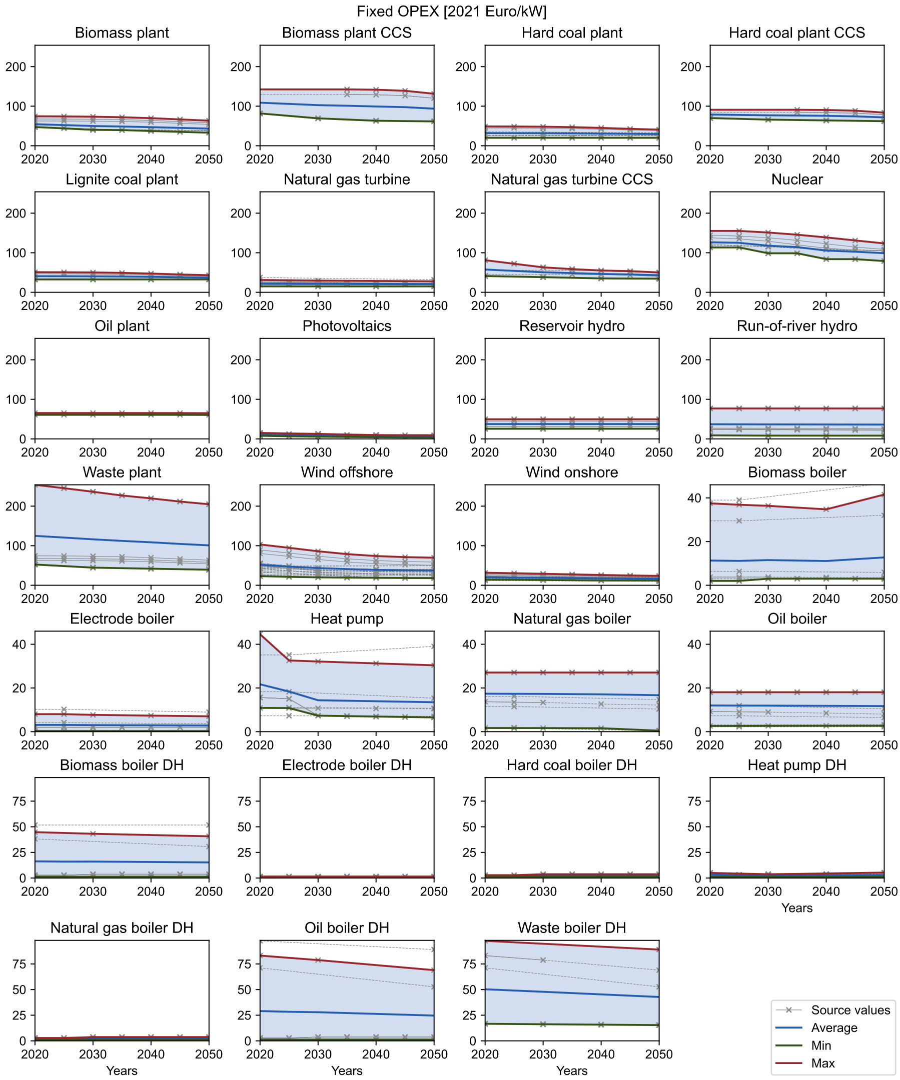


**Fig. S42** Evolution of fixed operational expenditure (fixed OPEX) for conversion technologies from several sources from 2020 to 2050 in 2021 EUR/kW (see Table S9). The average value is in blue, minimum in green, and maximum in red. The reported values are averaged for each source (grey lines) and averaged across sources. The dashed lines are the interpolation between values that are more than 5 years apart. DH: district heating. Related to STAR Methods.

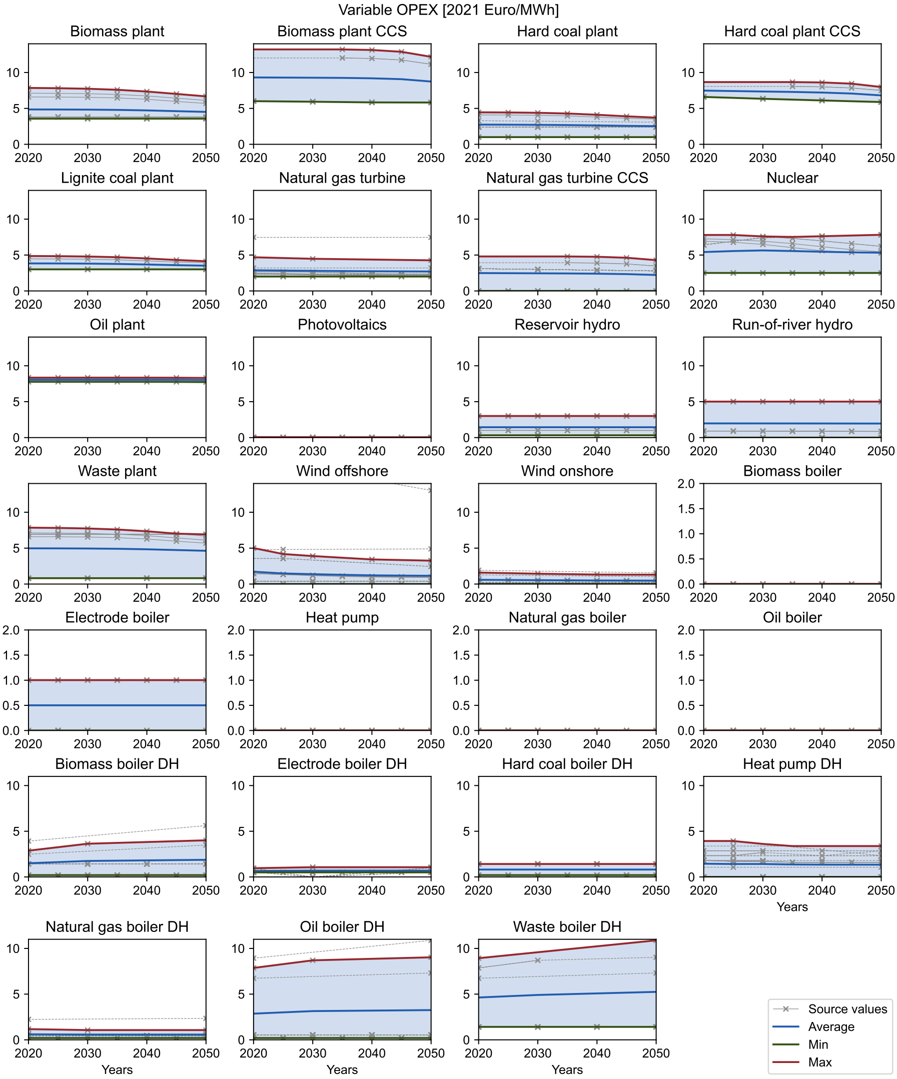


**Fig. S43** Evolution of variable operational expenditure (variable OPEX) for conversion technologies from several sources from 2020 to 2050 in 2021 EUR/MWh (see Table S9). The average value is in blue, minimum in green, and maximum in red. The reported values are averaged for each source (grey lines) and averaged across sources. The dashed lines are the interpolation between values that are more than 5 years apart. DH: district heating. Related to STAR Methods.

## S3.11 Technology expansion rates

In this study, we use historically observed expansion rates of low-carbon technologies as an estimate of how technologies might be expanded in the future. Fig. S44 shows the general three-step procedure of how the technology expansion rates are obtained, based on Leibowicz et al. [63]:

1. Blue markers: Obtain all past capacity additions of all relevant technologies in the electricity sector for all 28 European countries, (S3.2).
2. Red markers: Construct the capacity expansion frontier, i.e., the frontier of the highest capacity expansions in the past in the 28 European countries.
3. Green markers: Fit the technology expansion rate of solar PV, onshore wind, and offshore wind, the knowledge spillover rate, and the unbounded market share to approximate the frontier.

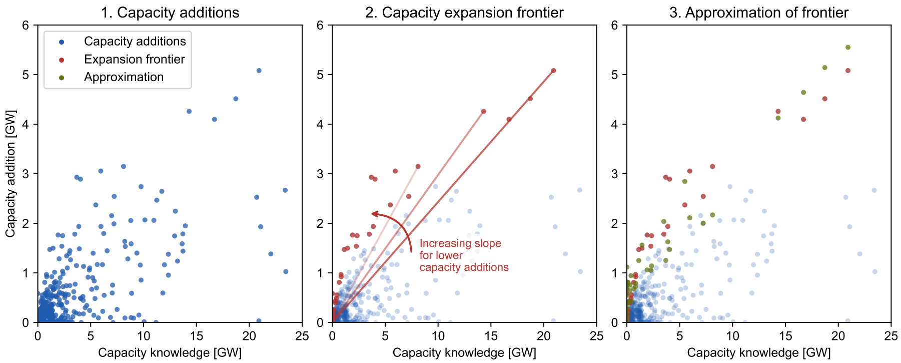


**Fig. S44** Step-by-step calculation of the technology expansion rates: 1. Obtaining past capacity additions and calculating corresponding capacity knowledge stock; 2. Constructing the capacity expansion frontier; 3. Approximating the frontier with our model of technology expansion. Example here for onshore wind; the same procedure is followed for solar PV and offshore wind. Related to STAR Methods.

Historic evidence suggests that the industrial expertise of the installation of energy technologies is depreciated as industrial knowledge fades over time [63]. For each technology $i \in \mathcal{I}$, country $n \in \mathcal{N}$, and year $y \in \mathcal{Y}$, the capacity knowledge stock, $K_{i,n,y}$, is calculated as [63]:

$$K_{i,n,y} = \sum_{\tilde{y}=-\infty}^{y-1} (1-\delta)^{(y-\tilde{y})} \, \Delta S_{i,n,\tilde{y}}, \tag{S43}$$

with the capacity addition, $\Delta S_{i,n,y}$, and the knowledge depreciation rate, $\delta = 0.1$ [63]. We include all capacity additions from the past ($\tilde{y} = -\infty$), with the first capacity addition reported in 1981 (onshore wind) until 2023 [64]. Note that we calculate the knowledge stock of historic capacity additions identically to how the knowledge stock is calculated in the optimization model (Eq. (S36)).

The maximum possible rate at which capacity was expanded in the past is represented by the capacity expansion frontier (second panel of Fig. S44). The frontier describes the highest observed capacity additions in the past, $\Delta S^{\mathrm{f}}_{i,n,y}$, relative to the existing capacity knowledge stock at the time. All points below the frontier indicate capacity additions that were lower than what was possible, given the industrial expertise and landscape.

The frontier in Fig. S44 shows a steep slope at low capacity knowledge stocks and then a lower slope at higher capacity knowledge stocks. This observation supports the existence of spillover and market entry effects [65, 66]. Once a technology is more established, i.e., has substantial capacity knowledge, the past capacity additions determine the future expansion.

We construct the frontier by sorting the capacity additions in descending order and adding those points to the frontier for which the slope between knowledge stock and capacity addition is increasing (second panel of Fig. S44). Hence, the next smaller capacity addition becomes part of the frontier if the relative addition with respect to the knowledge stock is increasing. To reduce the impact of outliers, we include all points that are within the top 5 highest slopes. By doing so, we include more points in the frontier which improves the subsequent frontier approximation. Five points proved to be a good number to include enough points without fraying the frontier.

Finally, the capacity expansion frontiers for the renewable technologies $\mathcal{I}^{\mathrm{ren}}$ solar PV, onshore wind, and offshore wind are approximated (third panel of Fig. S44) to yield the historical expansion rates, $\vartheta_i$, the knowledge spillover rate between nodes, $\omega$, and the unbounded market share, $\xi$. We deploy the same model formulation as in the optimization problem to ensure that the obtained parameter values are appropriate to be used in the optimization model (Eq. (S31)). The modeled capacity additions in the frontier, $\Delta S^{\mathrm{f},*}_{i,n,y}$, of technology $i$ in country $n$ and year $y$ are described as:

$$\Delta S^{\mathrm{f},*}_{i,n,y} = \vartheta_i \left( K_{i,n,y} + \omega \sum_{\tilde{n} \in \tilde{\mathcal{N}}} K_{i,\tilde{n},y} \right) + \xi \sum_{\tilde{i} \in \tilde{\mathcal{I}}} S_{\tilde{i},n,y-1}, \tag{S44}$$

with all other nodes $\tilde{\mathcal{N}} = \mathcal{N} \setminus \{n\}$ and all technologies with the reference carrier electricity $\tilde{\mathcal{I}}$ (Eq. (S30)). The sign in Eq. (S31) is changed from $\leq$ to $=$ in Eq. (S44) since we only consider the capacity additions in the frontier, thus the highest possible capacity addition. We use the same technical lifetime as in the optimization model to calculate the existing capacities of each technology. Note that we include all electricity generation technologies in the existing capacity $\sum_{\tilde{i} \in \tilde{\mathcal{I}}} S_{\tilde{i},n,y-1}$ to describe the total electricity generation capacity in the previous year.

The objective function to obtain the best approximation of $\vartheta_i$, $\omega$, and $\xi$ is formulated as:

$$\min_{\vartheta_i,\omega,\xi} \sum_{i\in\mathcal{I}^{\text{ren}}} \sum_{n\in\mathcal{N}} \sum_{y\in\mathcal{Y}} \left(\Delta S^{\text{f},*}_{i,n,y} - \Delta S^{\text{f}}_{i,n,y}\right)^2. \tag{S45}$$

The obtained expansion parameters and the $R^2$ value are:

$$\begin{aligned}
\vartheta_{\text{PV}} &= 0.289\\
\vartheta_{\text{onshore}} &= 0.131\\
\vartheta_{\text{offshore}} &= 0.100\\
\omega &= 0.070\\
\xi &= 0.021\\
\\
R^2 &= 0.754
\end{aligned}$$

## S4 Calculation of stranded assets and total annual technology cost

An asset is considered stranded if it is decommissioned after $(y-\tilde{y})$ years of operation but has not yet reached the end of its lifetime, $l_h$, with $(y-\tilde{y}) < l_h$ [67]. The stranded asset, $A^{\mathrm{s}}_{i,n,y,\tilde{y}}$, of capacities built in year $\tilde{y}$ is often calculated as the remaining linear fraction of the initial capital investment $\alpha_{i,\tilde{y}}\Delta S_{i,n,\tilde{y}}$ [68]:

$$A^{\mathrm{s}}_{i,n,y,\tilde{y}} = \alpha_{i,\tilde{y}}\Delta S_{i,n,\tilde{y}}\frac{l_i-(y-\tilde{y})}{l_i}. \tag{S46}$$

In general, the stranded capital is calculated for single assets [67, 68], not cumulative national capacities as is the case in our study. Hence, we calculate what share of the total usable capacity is not utilized, with the utilization rate, $\Xi_{i,n,y}$:

$$\Xi_{i,n,y} = \frac{\sum_{t\in\mathcal{T}} G^{\mathrm{r}}_{i,n,t,y}}{\sum_{t\in\mathcal{T}} m_{i,n,t,y}S_{i,n,y}}. \tag{S47}$$

The total stranded capacity, $S^{\mathrm{s}}_{i,n,y}$, in year $y$ is then:

$$S^{\mathrm{s}}_{i,n,y} = S_{i,n,y}(1-\Xi_{i,n,y}). \tag{S48}$$

We assume that the oldest capacities are the first to be decommissioned, thus, we sum up the oldest capacity additions to $S^{\mathrm{s}}_{i,n,y}$ and calculate the respective stranded assets (Eq. (S46)). By assuming the oldest capacities to be decommissioned first, our estimate of the stranded capital may be a lower bound of the actual stranded capital.

The total annual cost of a technology is calculated as the annual capital and operational expenditure plus all upstream and downstream costs. The upstream costs contain fuel costs and account for losses in the transport and storage technologies. The costs of other technologies and energy carriers in the upstream and downstream costs are weighted by the fraction of the consumed or produced energy carrier by the technology.

## S5 Comment on the use of rolling horizon and myopic foresight to reduce computational burden

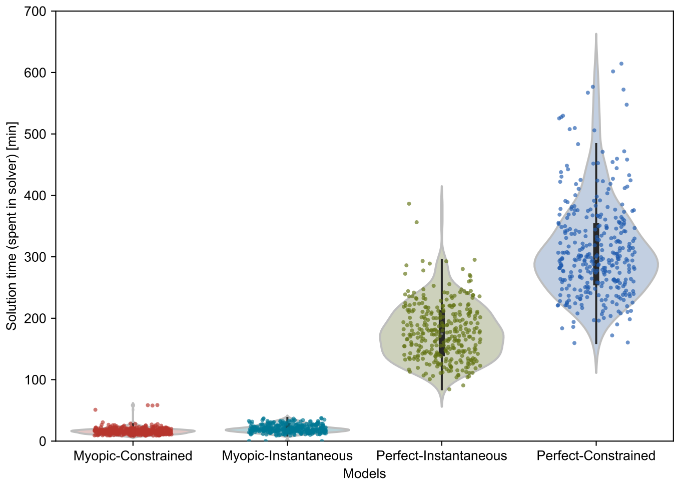


**Fig. S45** Solution time (spent in solver) for each model category: Myopic-Constrained (red, n = 442), Myopic-Instantaneous (turquoise, n = 345), Perfect-Instantaneous (green, n = 329), Perfect-Constrained (blue, n = 338). Note that the model runs used for this visualization include preliminary optimizations and are not entirely equal in the input data or the results. The model setup is equal for all runs in each category, i.e., the number of operational time steps, the number of years, and the scope of the energy system. Only for visualization purposes of solution time. All model categories show comparable coefficients of variation (Myopic-Constrained: 0.345, Myopic-Instantaneous: 0.315, Perfect-Instantaneous: 0.255, Perfect-Constrained: 0.250). Data are represented as individual points and as mean +/- one quartile. Related to STAR Methods.

The solution complexity of state-of-the-art active-set (simplex) and interior-point (barrier) solvers does not increase linearly but generally polynomially or, in the worst case, exponentially with the problem size [69, 70]. Thus, rolling horizon optimizations with myopic foresight are often used to reduce the computational burden of the solution process by partitioning a single large problem into many small subproblems [71–73].

In our study, the same impact on the solution time is observed between myopic foresight and perfect foresight optimizations (Fig. S45). The myopic foresight optimizations spend, on average, 16 min (Myopic-Constrained) and 21 min (Myopic-Instantaneous) in the solver to complete the 15 optimizations with single-step foresight. Perfect-Instantaneous, on the other hand, shows an increased computational burden of around 180 min (elevenfold in comparison to Myopic-Constrained). Even more pronounced, Perfect-Constrained faces a significant increase in computational complexity because of the year-coupling in the technology expansion constraints, which increases the average computation time to 320 min.

We argue in this study that the introduction of myopic foresight changes the general paradigm of decision-making, which is fundamental to the investigated research question and described energy system. This study and many other studies in the literature underline the impact of myopic foresight on the transition pathway in energy system optimization models [2, 17, 74–76]. While it is understandably desirable to reduce the computational burden, we strongly recommend not to use myopic foresight as a shortcut to reduce the solution time without making sure that this does not alter the general paradigm of decision-making. The modeling decision to use myopic foresight or perfect foresight should be motivated by the description of decision-making, not the desire to reduce computation time.

# References


[1] IPCC: SYNTHESIS REPORT OF THE IPCC SIXTH ASSESSMENT REPORT (AR6) - Summary for Policymakers. Technical report, IPCC (2023). Publication Title: Ipcc. https://www.ipcc.ch/report/ar6/syr/ Accessed 2023-03-21

[2] Fuso Nerini, F., Keppo, I., Strachan, N.: Myopic decision making in energy system decarbonisation pathways. A UK case study. Energy Strategy Reviews **17**, 19–26 (2017). https://doi.org/10.1016/j.esr.2017.06.001

[3] Kranzl, L., Hartner, M., Müller, A., Resch, G., Fritz, S., Fleiter, T., Herbst, A., Rehfeldt, M., Manz, P., Zubaryeva, A., Vilchez, G.: Hotmaps 2030/2050 scenarios for the heating and cooling sectors (2018). https://www.hotmaps-project.eu/hotmaps-2030-2050-scenarios-for-the-heating-and-cooling-sectors/ Accessed 2024-04-08

[4] European Environment Agency: Electric vehicle energy demand as a fraction of total electricity demand per country in 2050 (2016). https://www.eea.europa.eu/data-and-maps/daviz/ev-share-of-total-electricity/embed-chart?chart=chart_1 Accessed 2024-04-08

[5] Malhotra, A., Schmidt, T.S.: Accelerating Low-Carbon Innovation. Joule **4**(11), 2259–2267 (2020). https://doi.org/10.1016/j.joule.2020.09.004. Accessed 2023-08-25

[6] International Energy Agency: Hydropower Special Market Report: Analysis and forecast to 2030. Technical report, OECD (July 2021). https://doi.org/10.1787/07a7bac8-en. https://www.oecd-ilibrary.org/energy/hydropower-special-market-report_07a7bac8-en Accessed 2023-11-21

[7] Rennert, K., Errickson, F., Prest, B.C., Rennels, L., Newell, R.G., Pizer, W., Kingdon, C., Wingenroth, J., Cooke, R., Parthum, B., Smith, D., Cromar, K., Diaz, D., Moore, F.C., Müller, U.K., Plevin, R.J., Raftery, A.E., Ševčíková, H., Sheets, H., Stock, J.H., Tan, T., Watson, M., Wong, T.E., Anthoff, D.: Comprehensive evidence implies a higher social cost of CO2. Nature 2022 610:7933 **610**(7933), 687–692 (2022). https://doi.org/10.1038/s41586-022-05224-9. Publisher: Nature Publishing Group. Accessed 2023-01-18

[8] Mannhardt, J., Gabrielli, P., Sansavini, G.: Collaborative and selfish mitigation strategies to tackle energy scarcity: The case of the European gas crisis. iScience **26**(5), 106750 (2023). https://doi.org/10.1016/j.isci.2023.106750. Publisher: Elsevier

[9] Hoffmann, M., Priesmann, J., Nolting, L., Praktiknjo, A., Kotzur, L.,

Stolten, D.: Typical periods or typical time steps? A multi-model analysis to determine the optimal temporal aggregation for energy system models. Applied Energy **304**, 117825 (2021). https://doi.org/10.1016/J.APENERGY.2021.117825. Publisher: Elsevier

[10] Hofmann, F.: Linopy: Linear optimization with n-dimensional labeled variables. Journal of Open Source Software **8**(84), 4823 (2023). https://doi.org/10.21105/joss.04823. Accessed 2023-11-06

[11] Gurobi: Gurobi Optimizer Reference Manual 9.5 (2022). https://www.gurobi.com/documentation/9.5/

[12] Gabrielli, P., Gazzani, M., Martelli, E., Mazzotti, M.: Optimal design of multi-energy systems with seasonal storage. Applied Energy **219**, 408–424 (2018). https://doi.org/10.1016/J.APENERGY.2017.07.142. Publisher: Elsevier

[13] Blanke, T., Schmidt, K.S., Göttsche, J., Döring, B., Frisch, J., van Treeck, C.: Time series aggregation for energy system design: review and extension of modelling seasonal storages. Energy Informatics **5**(1), 17 (2022). https://doi.org/10.1186/s42162-022-00208-5. Accessed 2023-11-20

[14] Aryanpur, V., O'Gallachoir, B., Dai, H., Chen, W., Glynn, J.: A review of spatial resolution and regionalisation in national-scale energy systems optimisation models. Energy Strategy Reviews **37**, 100702 (2021). https://doi.org/10.1016/j.esr.2021.100702. Accessed 2023-07-21

[15] Eurostat: Complete energy balance nrg_bal_c (2022). https://ec.europa.eu/eurostat/databrowser/view/NRG_BAL_C/default/table?lang=en&category=nrg.nrg_quant.nrg_quanta.nrg_bal

[16] Danish Energy Agency: Technology Data for Generation of Electricity and District Heating. Technical report, Danish Energy Agency (June 2022). https://ens.dk/en/our-services/projections-and-models/technology-data/technology-data-generation-electricity-and Accessed 2022-11-06

[17] Löffler, K., Burandt, T., Hainsch, K., Oei, P.Y.: Modeling the low-carbon transition of the European energy system - A quantitative assessment of the stranded assets problem. Energy Strategy Reviews **26**, 100422 (2019). https://doi.org/10.1016/J.ESR.2019.100422. Publisher: Elsevier

[18] European Commission: EU Reference Scenario 2020. Technical report, European Commission (2020). https://energy.ec.europa.eu/data-and-analysis/energy-modelling/eu-reference-scenario-2020_en Accessed 2023-07-11

[19] Mantzos, L., Wiesenthal, T., Neuwahl, F., Rózsai, M.: The POTEnCIA Central scenario: an EU energy outlook to 2050. JRC Science for Policy Report, 346 (2019). https://doi.org/10.2760/32835. ISBN: 978-92-76-12010-0 Publisher: European Commission, Joint Research Centre. Accessed 2022-11-06

[20] Breyer, C., Bogdanov, D., Ram, M., Khalili, S., Vartiainen, E., Moser, D., Román Medina, E., Masson, G., Aghahosseini, A., Mensah, T.N.O., Lopez, G., Schmela, M., Rossi, R., Hemetsberger, W., Jäger-Waldau, A.: Reflecting the energy transition from a European perspective and in the global context—Relevance of solar photovoltaics benchmarking two ambitious scenarios. Progress in Photovoltaics: Research and Applications (2022). https://doi.org/10.1002/PIP.3659. Publisher: John Wiley and Sons Ltd

[21] Cui, R.Y., Hultman, N., Edwards, M.R., He, L., Sen, A., Surana, K., McJeon, H., Iyer, G., Patel, P., Yu, S., Nace, T., Shearer, C.: Quantifying operational lifetimes for coal power plants under the Paris goals. Nature Communications 2019 10:1 **10**(1), 1–9 (2019). https://doi.org/10.1038/s41467-019-12618-3. Publisher: Nature Publishing Group. Accessed 2023-04-21

[22] Ruhnau, O., Hirth, L., Praktiknjo, A.: Update and extension of the When2Heat dataset. Scientific Data **6**(1) (2022). https://doi.org/10.1038/S41597-019-0199-Y. Publisher: Kiel, Hamburg: ZBW – Leibniz Information Centre for Economics. Accessed 2022-11-07

[23] Danish Energy Agency: Technology Data for Transport of Energy (2021). https://ens.dk/en/our-services/projections-and-models/technology-data/technology-data-transport-energy Accessed 2023-07-13

[24] Brauers, H., Braunger, I., Jewell, J.: Liquefied natural gas expansion plans in Germany: The risk of gas lock-in under energy transitions. Energy Research and Social Science **76**, 102059 (2021). https://doi.org/10.1016/J.ERSS.2021.102059. Publisher: Elsevier Ltd. Accessed 2022-04-26

[25] Equinor: Northern Lights – Reports. Technical report (2019). https://norlights.com/what-we-do/reports/ Accessed 2023-07-19

[26] Global CCS Institute: The costs of CO2 storage: post-demonstration CCS in the EU. Technical report (2011). https://www.globalccsinstitute.com/resources/publications-reports-research/the-costs-of-co2-storage-post-demonstration-ccs-in-the-eu/ Accessed 2023-07-13

[27] World Energy Council: Energy Storage Monitor. Technical report, World Energy Council (2019)

[28] Tröndle, T., Lilliestam, J., Marelli, S., Pfenninger, S.: Trade-Offs between Geographic Scale, Cost, and Infrastructure Requirements for Fully Renewable Electricity in Europe. Joule **4**(9), 1929–1948 (2020). https://doi.org/10.1016/J.JOULE.2020.07.018. Publisher: Cell Press

[29] Danish Energy Agency: Technology Data for Carbon Capture, Transport and Storage. Technical report (November 2021). https://ens.dk/en/our-services/projections-and-models/technology-data/technology-data-carbon-capture-transport-and Accessed 2023-07-13

[30] Schmidt, O., Melchior, S., Hawkes, A., Staffell, I.: Projecting the Future Levelized Cost of Electricity Storage Technologies. Joule **3**(1), 81–100 (2019). https://doi.org/10.1016/J.JOULE.2018.12.008. Publisher: Cell Press

[31] Victoria, M., Zeyen, E., Brown, T.: Speed of technological transformations required in Europe to achieve different climate goals. Joule **6**(5), 1066–1086 (2022). https://doi.org/10.1016/j.joule.2022.04.016. Publisher: Cell Press

[32] Bloomberg: BloombergNEF (2023). https://about.bnef.com/ Accessed 2023-07-17

[33] ENTSO-E: ENTSO-E Transparency Platform (2022). https://transparency.entsoe.eu/ Accessed 2022-11-15

[34] Open Power System Data: Data Package Renewable power plants. Version 2020-08-25 (2020). https://doi.org/10.25832/renewable_power_plants/2020-08-25. https://data.open-power-system-data.org/renewable_power_plants/2020-08-25 Accessed 2022-11-15

[35] Open Power System Data: Data Package Conventional power plants. Version 2020-10-01 (2020). https://doi.org/10.25832/conventional_power_plants/2020-10-01. https://data.open-power-system-data.org/conventional_power_plants/2020-10-01 Accessed 2022-11-15

[36] IRENA: Renewable Capacity Statistics 2022. Technical report, IRENA (April 2022). https://www.irena.org/publications/2022/Apr/Renewable-Capacity-Statistics-2022 Accessed 2023-07-17

[37] European Commission, Joint Research Center: JRC Hydro-power database (2019). https://data.europa.eu/data/datasets/52b00441-d3e0-44e0-8281-fda86a63546d Accessed 2022-11-15

[38] Zablocki, A.: Fact Sheet Energy Storage (2019). Technical report, EESI (February 2019). https://www.eesi.org/papers/view/energy-storage-2019 Accessed 2022-11-15

[39] Ruiz, P., Nijs, W., Tarvydas, D., Sgobbi, A., Zucker, A., Pilli, R., Jonsson, R., Camia, A., Thiel, C., Hoyer-Klick, C., Dalla Longa, F., Kober, T., Badger, J., Volker, P., Elbersen, B.S., Brosowski, A., Thrän, D.: ENSPRESO - an open, EU-28 wide, transparent and coherent database of wind, solar and biomass energy potentials. Energy Strategy Reviews **26**, 100379 (2019). https://doi.org/10.1016/J.ESR.2019.100379. Publisher: Elsevier

[40] Trondle, T.: Supply-side options to reduce land requirements of fully renewable electricity in Europe. PLOS ONE **15**(8), 0236958 (2020). https://doi.org/10.1371/JOURNAL.PONE.0236958. Publisher: Public Library of Science ISBN: 1111111111. Accessed 2022-11-15

[41] Gernaat, D.E.H.J., Bogaart, P.W., Vuuren, D.P.v., Biemans, H., Niessink, R.: High-resolution assessment of global technical and economic hydropower potential. Nature Energy **2**(10), 821–828 (2017). https://doi.org/10.1038/s41560-017-0006-y. Number: 10 Publisher: Nature Publishing Group. Accessed 2023-11-21

[42] Bogaart, P.: The potential for sustainable hydropower. Nature Water **1**(1), 22–23 (2023). https://doi.org/10.1038/s44221-022-00018-9. Number: 1 Publisher: Nature Publishing Group. Accessed 2023-11-21

[43] Quaranta, E., Georgakaki, A., Letout, S., Kuokkanen, A., Mountraki, A., Ince, E., Shtjefni, D., Joanny, O.G., Eulaerts, O., Grabowska, M.: Hydropower and Pumped Hydropower Storage in the European Union – 2022 Status Report on Technology Development, Trends, Value Chains and Markets. Technical report, Clean Energy Technology Observatory (November 2022). https://doi.org/10.2760/256255. ISBN: 9789276581031 ISSN: 1831-9424. https://publications.jrc.ec.europa.eu/repository/handle/JRC130587 Accessed 2023-11-20

[44] Portugal-Pereira, J., Ferreira, P., Cunha, J., Szklo, A., Schaeffer, R., Araújo, M.: Better late than never, but never late is better: Risk assessment of nuclear power construction projects. Energy Policy **120**, 158–166 (2018). https://doi.org/10.1016/j.enpol.2018.05.041. Publisher: Elsevier. Accessed 2023-05-22

[45] Nuclear Energy Agency: Nuclear Energy Data 2021. Technical report, OECD, Boulogne-Billancourt (2021). https://www.oecd-nea.org/jcms/pl_69894/nuclear-energy-data-2021?details=true Accessed 2023-07-21

[46] ENTSOG: Press Release - ENTSOG publishes its Yearly Supply Outlook 2022 / 2023 in response to disruption of Russian gas supply. Technical report, ENTSOG, Brussels (2022). Issue: July 2022. https://www.entsog.eu/press-releases Accessed 2022-06-10

[47] Staffell, I., Pfenninger, S.: Using bias-corrected reanalysis to simulate

current and future wind power output. Energy **114**, 1224–1239 (2016). https://doi.org/10.1016/J.ENERGY.2016.08.068. Publisher: Pergamon

[48] Pfenninger, S., Staffell, I.: Long-term patterns of European PV output using 30 years of validated hourly reanalysis and satellite data. Energy **114**, 1251–1265 (2016). https://doi.org/10.1016/J.ENERGY.2016.08.060. Publisher: Pergamon

[49] Paardekooper, S., Lund, R.S., Mathiesen, B.V., Chang, M., Petersen, U.R., Grundahl, L., David, A., Dahlbaek, J., Kapetanakis, I.A., Lund, H., Bertelsen, N., Hansen, K., Drysdale, D.W., Persson, U.: Heat Roadmap Europe 4: Quantifying the Impact of Low-Carbon Heating and Cooling Roadmaps. Technical report, Aalborg Universitetsforlag (2018). https://vbn.aau.dk/en/publications/heat-roadmap-europe-4-quantifying-the-impact-of-low-carbon-heatin Accessed 2022-11-07

[50] Fraunhofer Institute for Solar Energy Systems: Mapping and analyses of the current and future (2020 - 2030) heating/cooling fuel deployment (fossil/renewables). Technical report, European Commission (2016). https://energy.ec.europa.eu/publications/mapping-and-analyses-current-and-future-2020-2030-heatingcooling-fuel-deployment en Accessed 2023-07-19

[51] Eurostat: Disaggregated final energy consumption in households - quantities nrg_d_hhq (2022). https://ec.europa.eu/eurostat/databrowser/view/nrg_d_hhq/default/table?lang=en Accessed 2022-11-07

[52] Mantzos, L., Matei, N.A., Mulholland, E., Rózsai, M., Tamba, M., Wiesenthal, T.: JRC-IDEES 2015: Integrated Database of the European Energy Sector. Technical report, European Commission, Joint Research Centre (JRC) (2018). ISBN: 978-92-79-73465-6. https://data.jrc.ec.europa.eu/dataset/jrc-10110-10001 Accessed 2022-11-07

[53] Werner, S.: International review of district heating and cooling. Energy **137**, 617–631 (2017). https://doi.org/10.1016/j.energy.2017.04.045. Accessed 2023-07-13

[54] Eurostat: Population on 1 January by broad age group, sex and other typologies (urt_pjanaggr3) (2023). https://ec.europa.eu/eurostat/databrowser/view/URT_PJANAGGR3__custom_6637457/default/table?lang=en Accessed 2023-07-13

[55] Department for Environment, Food & Rural Affairs: Statistical Digest of Rural England (2022). https://www.gov.uk/government/statistics/statistical-digest-of-rural-england Accessed 2023-07-13

[56] Gabrielli, P., Rosa, L., Gazzani, M., Meys, R., Bardow, A., Mazzotti, M., Sansavini, G.: Net-zero emissions chemical industry in a world of limited resources. One Earth **6**(6), 682–704 (2023). https://doi.org/10.1016/j.oneear.2023.05.006. Accessed 2023-07-21

[57] IPCC: 2006 IPCC Guidelines for National Greenhouse Gas Inventories — IPCC. Technical report, IPCC (2006). https://www.ipcc.ch/report/2006-ipcc-guidelines-for-national-greenhouse-gas-inventories/ Accessed 2023-04-20

[58] Bloomberg: 2H 2022 Levelized Cost of Electricity Update. Section: Report (2022). https://about.bnef.com/blog/2h-2022-levelized-cost-of-electricity-update/ Accessed 2023-07-20

[59] Heat Roadmap Europe: Project Reports – Heat Roadmap Europe. Technical report (2017). https://heatroadmap.eu/project-reports/ Accessed 2023-07-20

[60] IOGP Europe: Map of CCUS Projects in Europe (2022). https://iogpeurope.org/resource/map-of-eu-ccus-projects/ Accessed 2023-07-06

[61] Global CCS Institute: Global Status of CCS 2022 (2023). https://www.globalccsinstitute.com/resources/global-status-of-ccs-2022/ Accessed 2023-07-06

[62] Entsog, Entso-e: TYNDP 2022 Scenario Report. Technical report (2022). https://2022.entsos-tyndp-scenarios.eu/ Accessed 2023-05-08

[63] Leibowicz, B.D., Krey, V., Grubler, A.: Representing spatial technology diffusion in an energy system optimization model. Technological Forecasting and Social Change **103**, 350–363 (2016). https://doi.org/10.1016/j.techfore.2015.06.001. Publisher: Elsevier Inc.

[64] BloombergNEF: New Energy Outlook 2022. Technical report, BloombergNEF (2022). https://about.bnef.com/new-energy-outlook/ Accessed 2023-05-08

[65] Grübler, A., Nakićenović, N., Victor, D.G.: Dynamics of energy technologies and global change. Energy Policy **27**(5), 247–280 (1999). https://doi.org/10.1016/S0301-4215(98)00067-6. Publisher: Elsevier

[66] Shahnazi, R., Dehghan Shabani, Z.: Do renewable energy production spillovers matter in the EU? Renewable Energy **150**, 786–796 (2020). https://doi.org/10.1016/J.RENENE.2019.12.123

[67] Semieniuk, G., Holden, P.B., Mercure, J.F., Salas, P., Pollitt, H., Jobson, K., Vercoulen, P., Chewpreecha, U., Edwards, N.R.,

Viñuales, J.E.: Stranded fossil-fuel assets translate to major losses for investors in advanced economies. Nature Climate Change **12**(6), 532–538 (2022). https://doi.org/10.1038/s41558-022-01356-y. ISBN: 4155802201356. Accessed 2023-07-03

[68] Edwards, M.R., Cui, R., Bindl, M., Hultman, N., Mathur, K., McJeon, H., Iyer, G., Song, J., Zhao, A.: Quantifying the regional stranded asset risks from new coal plants under 1.5 °C. Environmental Research Letters **17**(2), 024029 (2022). https://doi.org/10.1088/1748-9326/AC4EC2. Publisher: IOP Publishing. Accessed 2023-07-03

[69] Spielman, D.A., Teng, S.-H.: Smoothed Analysis of Algorithms: Why the Simplex Algorithm Usually Takes Polynomial Time. arXiv. arXiv:cs/0111050 (2003). https://doi.org/10.48550/arXiv.cs/0111050. http://arxiv.org/abs/cs/0111050 Accessed 2023-07-26

[70] Wright, S.J.: 3. Complexity Theory. In: Primal-Dual Interior-Point Methods. Other Titles in Applied Mathematics, pp. 49–63. Society for Industrial and Applied Mathematics, ??? (1997). https://doi.org/10.1137/1.9781611971453.ch3. https://epubs.siam.org/doi/10.1137/1.9781611971453.ch3 Accessed 2023-07-26

[71] Baumgärtner, N., Deutz, S., Reinert, C., Nolzen, N., Kuepper, L.E., Hennen, M., Hollermann, D.E., Bardow, A.: Life-Cycle Assessment of Sector-Coupled National Energy Systems: Environmental Impacts of Electricity, Heat, and Transportation in Germany Till 2050. Frontiers in Energy Research **9**, 27 (2021). https://doi.org/10.3389/fenrg.2021.621502. Publisher: Frontiers Media S.A.

[72] Babrowski, S., Heffels, T., Jochem, P., Fichtner, W.: Reducing computing time of energy system models by a myopic approach: A case study based on the PERSEUS-NET model. Energy Systems **5**(1), 65–83 (2014). https://doi.org/10.1007/s12667-013-0085-1

[73] Thomsen, J., Saad Hussein, N., Dolderer, A., Kost, C.: Effect of the Foresight Horizon on Computation Time and Results Using a Regional Energy Systems Optimization Model. Energies (2021). https://doi.org/10.3390/en14020495

[74] Heuberger, C.F., Staffell, I., Shah, N., Mac Dowell, N.: Impact of myopic decision-making and disruptive events in power systems planning. Nature Energy **3**(8), 634–640 (2018). https://doi.org/10.1038/s41560-018-0159-3. Publisher: Nature Publishing Group. Accessed 2022-01-04

[75] Keppo, I., Strubegger, M.: Short term decisions for long term problems - The effect of foresight on model based energy systems analysis. Energy

**35**(5), 2033–2042 (2010). https://doi.org/10.1016/J.ENERGY.2010.01.019. Publisher: Elsevier Ltd

[76] Gerbaulet, C., von Hirschhausen, C., Kemfert, C., Lorenz, C., Oei, P.Y.: European electricity sector decarbonization under different levels of foresight. Renewable Energy **141**, 973–987 (2019). https://doi.org/10.1016/J.RENENE.2019.02.099. Publisher: Pergamon
